# What Should Be Discussed When Considering a Vaginal Birth? A Delphi Consensus Study

**DOI:** 10.1111/1471-0528.70071

**Published:** 2025-11-18

**Authors:** Andrew Demetri, Anna Davies, Danya Bakhbakhi, Alexandra Hunt, Sharea Ijaz, Sheelagh McGuinness, Gemma Beasor, Gemma Clayton, Vicky Bradley, Eve Bunni, Carol Kingdon, Andrew Sharp, Christy Burden, Asma Khalil, Louise Kenny, Abi Merriel, the Options Collaborative Group, Deborah Lawlor, Gordon Smith, Jane Norman, Jon Heron, Dame Tina Lavender, David Lissauer, Emma McGoldrick, Simon Grant, Sherif Abdel‐Fattah, Laura Bonnet, Mairead Black, Samuel Finnikin, Amie Wilson, Alexandra Freeman, Pete Blair, Katherine Birchenall, Joanne Johnson, Abigail Johnson, Chloe de Souza, Aine Dempsey, Gabriella Snook

**Affiliations:** ^1^ University of Bristol Bristol UK; ^2^ Health Data Science University of Liverpool Liverpool UK; ^3^ Patient Representative Bristol UK; ^4^ Department of Women's and Children's Health University of Liverpool Liverpool UK; ^5^ Liverpool Women's NHS Foundation Trust Liverpool UK; ^6^ City St George's University of London London UK; ^7^ Faculty of Health and Life Sciences University of Liverpool Liverpool UK

**Keywords:** consensus, core information set, Delphi technique, informed consent, stakeholders, vaginal birth, women, women's health

## Abstract

**Objective:**

Spontaneous vaginal births are often the presumed choice, representing 45% of UK births. However, information about benefits and risks is inconsistently given, impacting decision‐making and experience. A Core Information Set (CIS) is an agreed set of information points discussed prior to a decision. We aimed to develop a CIS for vaginal birth.

**Design:**

A Delphi study was used to create the CIS. Information points were identified from a literature search, patient leaflets, interviews, and a survey. These informed a two‐round Delphi survey, where stakeholders rated item importance. Items rated critically important by ≥ 80% of parents or professionals, and of limited importance by < 15%, progressed to consensus meetings, where 20 parents and professionals discussed retained items. The final CIS was populated with an engagement group ensuring accessibility.

**Setting:**

The study took place in the UK, with participants recruited online.

**Population:**

Pregnant and postnatal women, birth partners, healthcare professionals, medicolegal professionals, and representatives from relevant organizations.

**Main Outcome:**

A CIS for vaginal birth.

**Results:**

77 information items were identified. In round 1 (631 participants) of the Delphi Survey, 84.5% were from the patient group and 15.5% from the professional group; in round 2 (228 participants), 74.3% were from the patient group and 25.7% from the professional group. 29 items met the criteria for consensus discussion. The final CIS includes 19 information points addressing: labour process, pain relief, labour complications, procedures or interventions during labour, experiences after birth, outcomes for the baby and labour environment.

**Conclusions:**

This CIS can facilitate discussions and support informed decision‐making about vaginal birth.

## 1 Introduction

Less than half of births in the UK are spontaneous vaginal births [1]. For many women, unless a caesarean birth (CB) is indicated, a vaginal birth is the presumed choice [2]. This assumed preference may have contributed to antenatal information about vaginal birth being inconsistent and insufficient [3]. Having greater knowledge about birth may influence women's decisions and improve experiences [4]. There is interest in developing decision aids to support birth choices [5, 6, 7, 8]. Supporting women to make informed choices is championed by the National Institute for Health and Care Excellence (NICE) [9], birth advocacy groups [10], and enshrined in law [11].

The physiological process of vaginal birth has benefits for mother and baby, including shorter hospital stays, decreased risk of maternal wound infection, and increased breastfeeding rates [12, 13, 14, 15]. Risks include shoulder dystocia, pelvic floor and birth trauma, or neonatal morbidity, which can impact a woman's, or baby's, long‐term health [16, 17]. Knowing these risks and benefits may influence decision making. The paucity of high‐quality antenatal information can have detrimental effects on pregnancy and labour experiences [18]. It increases anxieties due to fear of the unknown and leads to a disconnect between expectation and reality [16, 19, 20], contributing to reduced birth satisfaction, and post‐traumatic stress disorder [19, 20, 21].

Access to consistent, high‐quality antenatal information is imperative. Whilst women are not asked to consent to vaginal birth, the General Medical Council (GMC) document on decision making and consent requires that women are given appropriate information [22]. Information provision about CB and instrumental birth has improved, but there has been limited progress with information about vaginal birth [23, 24].

Core Information Sets (CIS) aim to improve the consistency and quality of information, whilst not overwhelming patients. A CIS is developed systematically, achieving consensus between patients and healthcare professionals about the key information that should be discussed prior to a treatment or clinical decision [25, 26].

We aimed to define a CIS for vaginal birth, to provide consistent, patient‐centred information to ensure women are well‐informed about vaginal birth [24].

## 2 Details of Ethical Approval

Approval was granted on 27th April 2022 by the University of Bristol Research Ethics Committee (Ref: 10530).

## 3 Methods

This study was registered with the Core Outcome Measures Effectiveness in Tests initiative (https://comet‐initiative.org/Studies/Details/2069), and adheres to their recommended protocol and development standards [27] and the COS‐Standards for Reporting guidance (Table S1) [28, 29]. The methodology was adapted from core outcome sets and CIS [30, 31, 32]. Reporting is in line with the DELPHISTAR standardised reporting recommendations (Table S2) [33]. The Delphi panel constitution, feedback iteration, and management of attrition are described in Stages 3 and 4. Stability of responses between rounds was assessed by comparing distributions of item ratings and participant demographics, following DELPHISTAR reporting recommendations. The protocol has been previously published [34]. The five‐stage process is detailed below.

### 3.1 Stage 1: Development of Long‐List

A ‘long‐list’ of all information points about vaginal birth was collated from a systematic review, patient information leaflets, interviews with antenatal and postnatal women, and a stakeholder survey.

#### 3.1.1 Scoping Review

A pragmatic literature search was conducted by an information specialist (SD) in September 2022, to identify outcomes and information points about vaginal birth. The search was limited to English language systematic reviews (2020–2022) and Cochrane reviews (2017–2022). These timeframes were applied due to the large volume of eligible papers, with data saturation likely within these periods. Studies were not excluded for methodological quality or risk of bias, as this was not relevant to information‐point extraction.

Patient information leaflets on vaginal birth were sourced from the Royal College of Obstetricians and Gynaecologists (RCOG) [35], Tommy's Pregnancy Information [36], and NHS Trusts [37]. Leaflets on instrumental birth and vaginal birth after caesarean (VBAC) were excluded as these are related to specific clinical decisions rather than to vaginal birth generally.

Two researchers (AD, SI) screened titles, abstracts, and full texts using Covidence software [38]. Information items were extracted from papers by multiple reviewers (AD, GS, ADe, SI). Extracted items included any measured outcome, risk, or complication reported. A pre‐piloted extraction form (Appendix S1) was used to extract items, and a similar form (Appendix S2) for patient leaflets.

#### 3.1.2 Interviews

Semi‐structured qualitative interviews were conducted with pregnant (≥ 12 weeks) and postnatal women, regardless of the mode of birth. Participants were recruited online via social media. Three researchers (AD, ADa, AM) conducted interviews, using a topic guide to explore participants' views on important information to share with expectant mothers (Appendix S3), recorded on an encrypted audio recorder. Interviews were transcribed and analyzed using thematic analysis [39]. Interviews were coded to identify information points about vaginal birth. Analysis was conducted in parallel with the interviews, ensuring that when data saturation was achieved, interviews concluded.

#### 3.1.3 Stakeholder Survey

An online survey to capture information points from stakeholder groups was undertaken. Stakeholders were all UK‐based, and included antenatal and postnatal women, birth partners, healthcare professionals who work alongside women in labour and postnatally, representatives from groups with an interest in women's birthing rights, and medicolegal experts with an interest in reproductive health. The survey was conducted online using REDCap software [40]. Participants' roles and demographics were collected before they were asked to list the information items they believed to be crucial for women to be informed of when considering a vaginal birth. Survey data were thematically analysed, and responses were coded to identify key information items. Codes were grouped to create themes and key information points were then identified from each of these [39].

### 3.2 Stage 2: Developing the Long List of Information Points

Using the exhaustive ‘long‐list’ from stage 1, the information items were grouped thematically into subcategories to support presentation. The core research team (AD, AM, ADa) met to establish how items should be worded in the survey, and to develop definitions for items to support understanding. Duplicate items were removed. We had planned to involve the whole research team in this process, but due to time constraints this was carried out by three members of the team.

The long‐list was presented as an online survey using REDCap [40]. It was piloted with four pregnant and postnatal women using Think‐Aloud interviews [41]. Changes were made to enhance the usability of the survey. The involvement of pregnant and postnatal women in this stage helped shape the final long‐list for the Delphi survey rounds.

### 3.3 Stage 3: The Delphi Survey

Survey participants were recruited through social media. Stakeholders included antenatal/postnatal women, birth partners, healthcare professionals (obstetricians, midwives, midwifery care assistants, anaesthetists, general practitioners and physiotherapists), representatives of birth‐rights groups, medicolegal experts and researchers with an interest in reproductive health.

The survey was presented in a two‐round modified Delphi process on REDCap [40]. Each round stayed open for approximately 8 weeks. Demographic data were collected at the start of the survey, including which stakeholder group they belonged to, age, area of residence, and level of education. All participants were asked about their parity and modes of birth. Stakeholders were subdivided into the parent/non‐professional group (antenatal and postnatal women, birth partners, representatives from interested groups) and professional group (healthcare professionals, medicolegal experts, researchers). Professionals were asked for information on their job role, work location and experience. Professionals who were pregnant, had a baby, or were partners of someone who was pregnant or had a baby, were included in the professional group.

In round 1, survey participants were asked to rate the importance of each individual item for inclusion in a CIS on a 9‐point Likert scale (1–3 limited importance; 4–6 important but not critical; 7–9 critical). This categorisation follows COMET Initiative recommendations for consensus studies. While scores of 7, 8 and 9 may reflect different levels of strength of opinion, grouping them ensures comparability with other Delphi studies and simplifies the consensus process. A priori exclusion criteria were applied to the removal of items between rounds: where ≥ 80% of one of the stakeholder groups (patients/professionals) voted the item as being of limited importance, and < 15% from either group believed it to be critically important, the item was removed before round 2.

In round 2, the Delphi survey with the retained items from the first round was redistributed to round 1 participants. Round 2 included information on: the participant's own score, the median score, and histograms representing the distribution of scores for parents and professionals for each point. Participants were asked to vote again using the same 9‐point scale.

Information items which were rated as critically important by ≥ 80% of participants, either parents or professionals independently, with < 15% from either group classifying as limited importance, were carried forward to the consensus meetings.

### 3.4 Stage 4: Consensus Meetings

An online consensus meeting was planned using Zoom video conferencing software [42]. Due to the number of items, two meetings were held. We purposively sampled Delphi participants (both parents and professionals) to achieve a mix of roles.

The consensus meeting participants were shown the median scores for both the parent and professional groups, as well as a graphical illustration of the distribution of scores for these groups. Participants were asked to discuss their views on each individual item, and whether it was vital to discuss with all women planning a vaginal birth. A nominal group technique was used for discussions, whereby participants were presented with the data, given some time to consider the item, and then encouraged to share ideas in a group discussion [43]. Participants were then asked to anonymously vote on items to ‘include’, ‘do not include’ or ‘unsure’. Voting was conducted using the in‐built voting system on Zoom, which does not tell participants who has voted for each option. Voting did not allow us to decipher how individuals (and thus the different groups) voted on each item. Consensus to include or exclude an item was defined a priori as ≥ 80% of participants agreeing to include it. Where consensus was not reached, further discussion and re‐voting took place, until a consensus was reached.

### 3.5 Stage 5: Populating the Vaginal Birth Core Information Set

The agreed CIS items were then populated with information from National Institute for Health and Care Excellence's guidelines, Royal College of Obstetrics and Gynaecologists' Green Top Guidelines and systematic reviews with a preference for Cochrane reviews due to their rigorous nature. Women and health professionals participating in an engagement group refined the content.

## 4 Results

### 4.1 Stage 1

The scoping review included 145 relevant papers and 29 patient information leaflets. Seventeen interviews with antenatal and postnatal women were undertaken, and 136 participated in the online stakeholder survey. 426 information items were identified.

### 4.2 Stage 2

Items identified in stage 1 were organised into a Delphi survey of 11 categories comprising 77 information items. Categories were: birth environment, labour process, pain relief, possible complications, possible intrapartum and postnatal procedures/interventions, experience after birth (short, medium, and long term), outcomes for the baby, and wider effects of birth (Appendix S4). Following piloting, the number of information items was reduced to avoid duplication, and wording and functionality of the survey were refined, until the survey was deemed usable and ready for circulation. This process produced 74 items for round 1 of the Delphi survey.

### 4.3 Stage 3

#### 4.3.1 Delphi Round 1

Demographic characteristics of the parent and professional participants group are detailed in Table 1. Pregnancy‐related demographics are shown in Table S3.

**TABLE 1:** Demographic characteristics of participants in round 1 and 2 of the Delphi process.

Patient group participants	Round 1 (*n* = 533)	Round 2 (*n* = 214)
*Role (able to select more than 1)*	*N (%)*	*N (%)*
Pregnant at time of completing survey	144 (27.0)	42 (19.6)
Previously been pregnant	335 (62.9)	179 (83.6)
Partner of someone who had been or is pregnant	81 (15.2)	34 (15.9)
Member of interested organisation or charity	6 (1.1)	6 (2.8)
*Sex*	*N (%)*	*N (%)*
Female	483 (90.6)	197 (92.1)
Male	50 (9.4)	17 (7.9)
Prefer not to say	0 (0.0)	0 (0.0)
Other	0 (0.0)	0 (0.0)
*Ethnicity*	*N (%)*	*N (%)*
White British	362 (67.9)	164 (76.6)
White other	61 (11.4)	13 (6.1)
Mixed/Multiple	32 (6.0)	17 (7.9)
Asian/Asian British	46 (8.6)	16 (7.5)
Black/African/Caribbean/Black British	23 (4.3)	4 (1.9)
Other	2 (0.4)	0 (0.0)
Prefer not to say	7 (1.3)	0 (0.0)
*Age*	*N (%)*	*N (%)*
Under 21	3 (0.6)	1 (0.5)
21–30	264 (49.5)	113 (52.8)
31–40	222 (41.7)	73 (34.1)
41–50	33 (6.2)	18 (8.4)
51–60	8 (1.5)	6 (2.8)
61–70	3 (0.6)	3 (1.4)
Prefer not to say	0 (0.0)	0 (0.0)
*Education level*	*N (%)*	*N (%)*
Pre‐GCSC	16 (3.0)	1 (0.5)
GCSE	45 (8.4)	10 (4.7)
A levels	76 (14.3)	16 (7.5)
Post grad degree	158 (29.6)	94 (43.9)
Bachelors/equivalent	236 (44.3)	93 (43.5)
Prefer not to say	2 (0.4)	0 (0.0)
Other	0 (0.0)	0 (0.0)
*Area of residence*	*N (%)*	*N (%)*
East of England	84 (15.8)	26 (12.1)
London	74 (13.9)	25 (11.7)
Midlands	38 (7.1)	16 (7.5)
North East England and Yorkshire	61 (11.4)	22 (10.3)
North West	99 (18.6)	42 (19.6)
Northern Ireland	33 (6.2)	8 (3.7)
Scotland	42 (7.9)	11 (5.1)
South East	35 (6.6)	18 (8.4)
South West	38 (7.1)	32 (15.0)
Wales	17 (3.2)	10 (4.7)
Other	12 (2.3)	4 (1.9)

Stakeholder group participants	Round 1 (*n* = 98)	Round 2 (*n* = 74)
*Stakeholder profession*	*N* (%)	*N* (%)
Midwives	33 (33.7)	25 (33.8)
Midwifery care assistants	5 (5.1)	2 (2.7)
O&G doctors	38 (38.8)	28 (37.8)
Anaesthetists	5 (5.1)	5 (6.8)
General practitioners	5 (5.1)	5 (6.8)
Medico‐legal experts	1 (1.0)	1 (1.4)
Researchers	5 (5.1)	2 (2.7)
Others	6 (6.1)	6 (8.1)
*Years held in position*	*N (%)*	*N (%)*
< 5 years	24 (24.5)	18 (24.3)
5–10 years	26 (26.5)	20 (27.0)
11–20 years	28 (28.6)	19 (25.7)
> 20 years	20 (20.4)	17 (23.0)

631 participants took part in round 1. We were not able to calculate the response rate for the survey due to the methods of recruitment. 533 (84.5%) were parents, birth partners or members of charity organisations, and 98 (15.5%) were professionals. 335 (62.9%) non‐professional participants had given birth to at least one baby, and 144 (27.0%) were pregnant. 92 (17.3%) participants from this group were partners of someone who had been or was pregnant, and a further six (0.02%) were from groups or charities with an interest in pregnancy and birth. Participants represented all regions of the United Kingdom. 362 (67.9%), identified as white British, and 61 (11.4%) identified as white other. 102 (19.1%) identified as being of non‐white ethnicity.

Of those that had previously given birth, 262 (78.2%) of these had experienced a spontaneous vaginal birth, 50 (14.9%) an assisted vaginal birth (forceps or ventouse), and 68 (20.3%) had experienced an emergency (11.3%) or elective (9.0%) caesarean.

From the professionals' group, 33 (33.7%) were midwives, and five were midwifery care assistants (5.1%). 38 (38.8%) were obstetric doctors, of which 25 (65.8%) were consultants or specialty doctors, and 13 (34.2%) were trainees or clinical fellows. Ten (10.2%) professionals were anaesthetists and general practitioners. One participant was a medicolegal expert and five were researchers.

None of the information items in round 1 (scoring shown in Table S4) met the predefined criteria for exclusion, and therefore all items were carried forward to round 2.

Analysis of the free text comments resulted in three new items relating to family planning, comparisons with other modes of birth, and after care immediately following vaginal birth.

#### 4.3.2 Delphi Round 2

Of the 631 completing round 1, 526 provided an active email for round 2. Of these, 288 completed round 2, representing an attrition rate of 45.2%. 214 (74.3%) participants were patients. Professionals made up a larger proportion in round 2 (R2:25.7%) than round 1 (R1:15.5%). Among parous women, mode of birth proportions were broadly similar across rounds. Vaginal birth remained most common (R1:78.2% vs. R2:84.9%). Assisted vaginal births were slightly higher in round 2 (R1: 14.9% vs. R2:17.9%), while emergency (R1:11.3% vs. R2:8.9%) and elective (R1:9.0% vs. R2: 6.1%) caesareans were lower in round 2. Thus, attrition did not substantially alter the distribution of birth experiences.

29 items met the criteria to be included in the consensus meetings. Table S5 summarises the survey results for all items in round 2, with those rated as critical (7–9) for inclusion in the CIS by > 80% of participants from either group highlighted.

### 4.4 Stage 4

Two online consensus meetings were undertaken to support attendance and ensure adequate time for discussion. 20 participants attended both meetings. The participants were nine parent representatives (six pregnant women who previously had a baby, three members of interested charities or organisations), three researchers, and eight from the professionals' group (five obstetricians, three midwives). Attendance was comparable across meetings, with a similar mix of parent and professional participants, facilitating balanced discussions. As half the participants were from the patient group, as aimed for in the protocol, equal weighting was given to all votes.

Of the 29 items carried forward, 12 met the pre‐specified inclusion criteria for the CIS without needing discussion in the consensus meeting, as > 90% of participants from one or both groups had scored them as critical in the second Delphi round. These information points are highlighted in blue in Table S5, and the final items are shown in Table S6.

Before the meetings, the study team reviewed closely related items and proposed mergers. These were presented to consensus meeting participants and agreed upon through discussion and anonymous Zoom voting (‘yes’, ‘no’ or ‘unsure’) [42]. For example, “Pelvic floor injury” and “Bladder or bowel symptoms” became “Pelvic floor injury and potential issues with this area following birth.” “Choice of birth location” and “Transfer of birth location during labour” merged into “Choice of where to give birth.” “The process of speeding up labour” was incorporated into “How the stages of labour are defined, and the expected progress.” Two items merged with two already meeting automatic inclusion thresholds (“How the stages of labour are defined…” and “How a baby's wellbeing is checked”); discussions addressed both merging and inclusion, resulting in the automatic inclusion of both. A full list of merged items, including the process and rationale, is outlined in Table S7.

After combining items, the participants discussed and voted. If no consensus was reached, further discussions preceded another vote. Voting results appear in Table S8. Both meetings used identical item lists, formats, and thresholds. Overlapping items were re‐presented in the second meeting for confirmation. No inconsistencies emerged between meetings.

**FIGURE 1:**
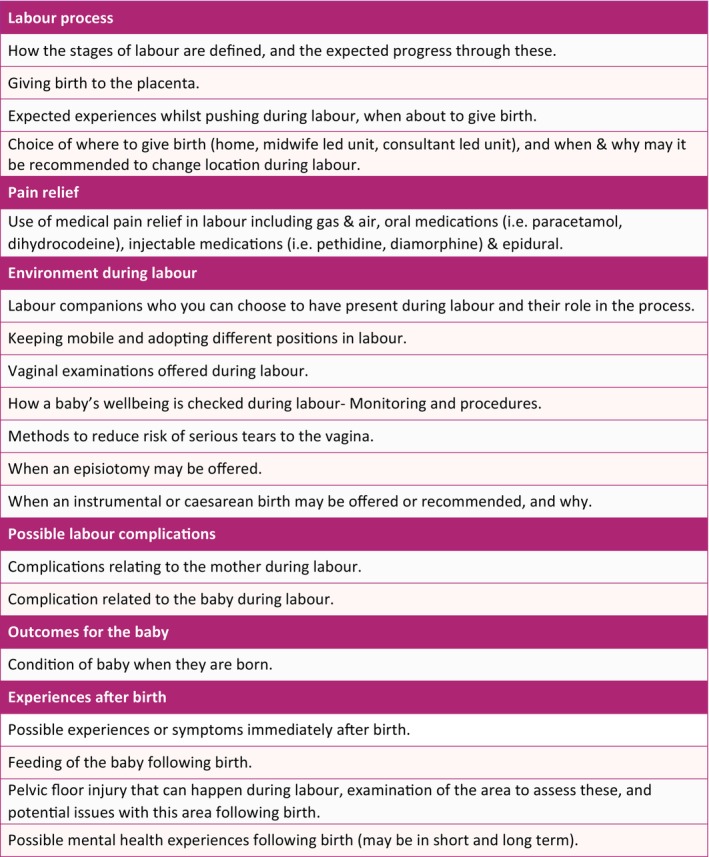
Vaginal birth core information set (in seven domains).

**FIGURE 2:**
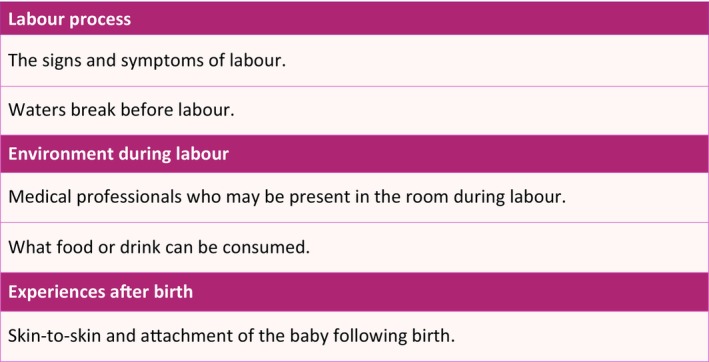
Vaginal birth supplementary list of information items.

From the discussed items the meeting participants derived two distinct groups of CIS items. The first of these was items related to decision making about whether to have a vaginal birth, which was determined to be the key purpose of this core information set. The second was important information *after* having decided to have a vaginal birth, to ensure they are as prepared as possible. Following the voting, 19 items were included in the final vaginal birth CIS, which were categorised into seven domains (Figure 1 and Table S9 for more detail). The consensus group wanted an additional supplementary list of 5 items for sharing once a vaginal birth was planned (Figure 2 and Table S10 for more detail).

A total of 74 items entered round 1 of the Delphi survey, all of which proceeded to round 2. Following scoring, 29 met the criteria for discussion at consensus meetings, where overlapping items were also merged. The final CIS comprised 19 items.

### 4.5 Stage 5

Seven Patient and Public Involvement and Engagement (PPIE) groups were held with 22 participants to populate and refine the content (Appendices S5 and S6). The PPIE groups involved midwives, parents, researchers, members from interested organisations, charity workers, a statistician and meetings with risk communication experts. Diagrams displaying anatomical details were requested along with additional detail available in a longer version of the CIS.

## 5 Discussion

### 5.1 Main Findings

The final CIS contains 19 key information points, grouped into seven domains, to support discussions and informed decision‐making about vaginal birth. These domains are labour process, pain relief, procedures or interventions, potential complications, postnatal experiences, outcomes for baby, and environment during labour, as well as potential risks to mother and/or baby. An additional 5 information points are included for after vaginal birth is opted for. Discussing these domains will ensure that health professionals have addressed essential information, agreed upon by parents and professionals, to promote a better understanding of childbirth.

Few items in round 1 exceeded 70% agreement within the patient group, reflecting the wide range of women's priorities and experiences. Differences between patients and professionals were anticipated, and the Delphi process with consensus meetings brought these perspectives together, ensuring the final CIS captured what both groups regarded as essential.

The 19 information points are more than other CIS's contain [25, 26]. Our aim was to produce up to 15 points [34] but a flexible approach was adopted as childbirth is a broad subject [34].

Much of this information is available within patient literature, though not always in an accessible form. For instance, patient leaflets cover the stages of labour and what to expect [44], pain relief options [45, 46, 47], and procedures like CB, instrumental birth, and episiotomy [48]. NICE guidelines discuss potential complications and risks of different birth modes [49], while postnatal care literature addresses recovery and emotional changes [50]. Baby outcomes and long‐term effects [51], and the labour environment are explored in various resources [52].

Now that the most valuable and critical‐to‐know information has been identified and compiled as a single CIS, it is important to develop methods to best communicate this information effectively in everyday practice [26, 53]. This could include using the information to standardise conversations women have with healthcare professionals about their mode of birth [54], creating visual aids to demonstrate risks [55], and informing, updating or linking into existing patient information leaflets and online resources. In partnership with an engagement group, we have populated the vaginal birth core information set (Appendices S5 and S6), to ensure it aligns with the communication needs of those who will use it. The resource incorporates the context‐specific and evidence‐based statistics for each item, whilst utilising language and visual aids which women find accessible. This can be used as a starting point for discussions about mode of birth. However, it is a minimum set of information and should not preclude discussion of other information, tailored to individuals' needs based on their own history, risk factors and desire for information [56, 57].

This study created a core information set to improve communication and decision‐making about vaginal birth. The study was based in the UK, but it can be used as a guide to the information that should be discussed about vaginal birth in any setting, as long as the content of the CIS is tailored to local information. Low and middle‐income settings may be able to use the identified core information set but populate it using careful translation, regional statistics and cultural contextualisation to ensure its relevance.

**FIGURE 3:**
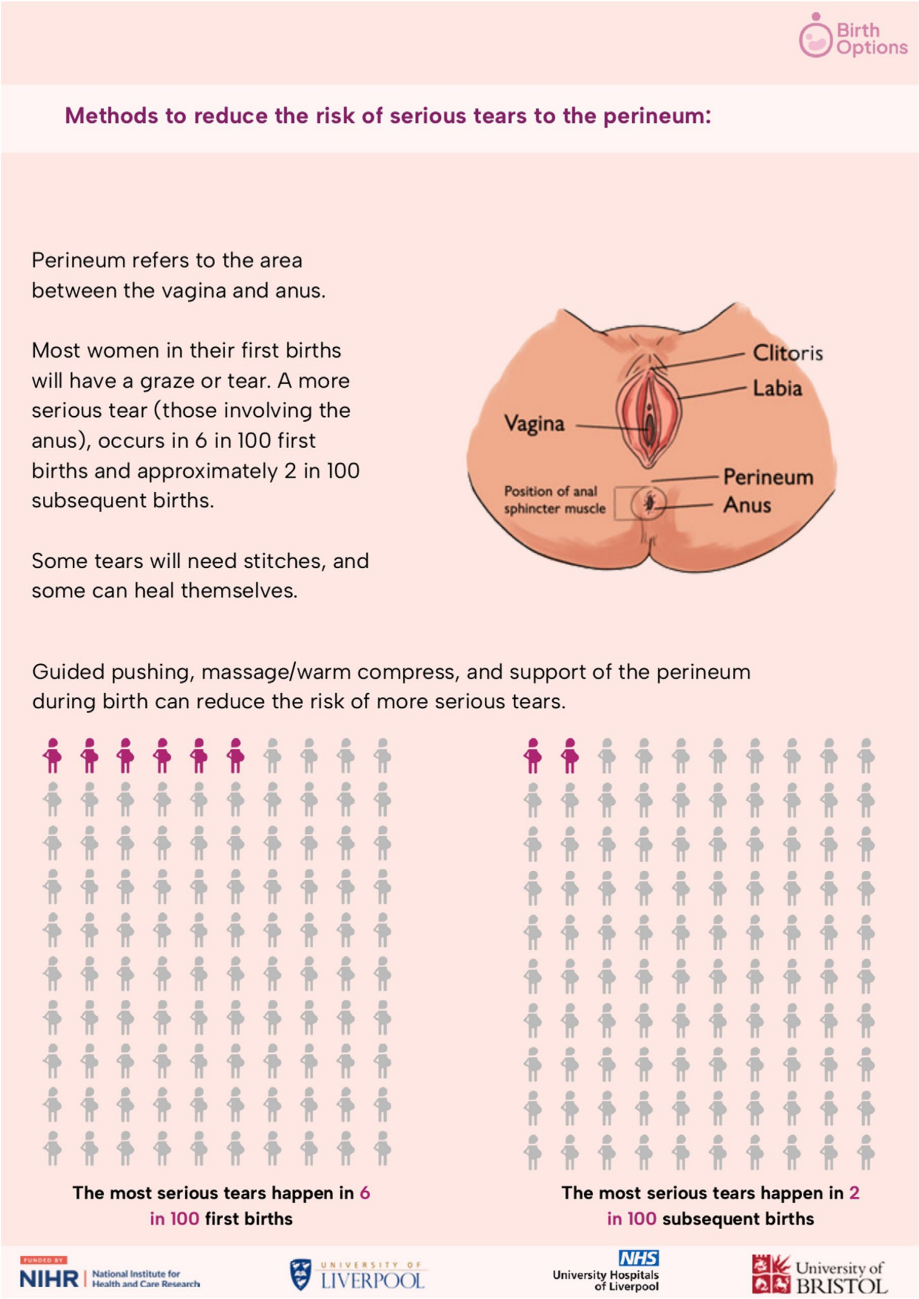
Example infographic from vaginal core information set summary document.

Delivering all 19 information points within current antenatal care structures may be challenging. Discussions may need to span several community midwife appointments, supported by written or digital resources, with obstetricians in high‐risk clinics tailoring content to individual priorities. We have provided the CIS documents (in summary (Appendix S5) and a more detailed form (Appendix S6)) which can be shared with women; we are also developing a website to share this information (www.birthoptions.co.uk). An example diagram from the summary document is shown in Figure 3.

### 5.2 Strengths and Limitations

The iterative process involved more than 600 stakeholders. Importantly, the process has had majority participation by pregnant women, or those who have given birth in the past, ensuring representation of the views of the end‐users. The online process allowed for national participation, reflected positively in the varied geographical locations of participants. Additionally, there was good diversity among the participants, with 19.2% in the first round and 17.3% in the second round of the survey identifying as non‐White ethnicity, showing engagement of diverse groups [58]. Recruitment via social media facilitated wide national reach and inclusion of diverse voices, though using these methods may favor digitally engaged participants with stronger views. Thus, priorities identified may not fully reflect those less active online or harder to reach. However, the large and varied sample, including patients and professionals, helps mitigate this risk.

We used a systematic and comprehensive approach to identifying potential items and a rigorous Delphi survey process. We achieved good engagement of patients and professionals in two rounds. We aimed to balance having a small but representative group of participants for the CIS consensus meetings to ensure manageable discussions. However, the number of participants was commensurate with other Delphi consensus meetings [25, 26, 32]. Extending the meeting across two dates allowed for the facilitation of more meaningful engagement.

The Delphi survey had an attrition rate of 45.2% between round one and two. This was higher than our aim of 20% [59]. Delphi survey attrition rates vary, from 20% to 90% [60]. Therefore an attrition rate of up to 50% is reasonable. The attrition was greatest in the patient group; however, there remained a high majority of patients compared to professionals. Demographics within the patient group remained similar between rounds. This mitigated the risk of underrepresentation of these voices in the second round.

This work addressed information points regarding spontaneous vaginal birth, not comparisons with other modes of birth. Our aim was to create a minimum, standardised information set for vaginal birth. Parallel CIS's for induction of labour and caesarean birth are completed to enable direct comparison [61, 62]. We chose not to include individual maternal risk factors (e.g., hypertension, fetal growth restriction, diabetes, high BMI), as they are variable and context‐specific. The CIS instead offers universally relevant information, to be supplemented by personalised discussions based on medical history and risk profile.

The a priori definitions for the inclusion of items for the consensus meeting and the final core information set allowed for a replicable and objective process.

## 6 Conclusion

This vaginal birth CIS can be used as a minimum list of points to support discussions between healthcare professionals and women planning a vaginal birth. It should be tailored to individual circumstances, needs, and expectations. This CIS can improve the information received by women to support their decision‐making by reducing variation in the key elements discussed.

## Author Contributions

ADemetri and A.M. conceived the study, obtained the funding, drafted the protocol, carried out the study and wrote the first version of the manuscript. ADavies helped with drafting the protocol and carrying out the study. D.B., C.B., and A.S. helped with formulating the methods for the study. S.I., S.M. and G.C. assisted with conceiving and carrying out stage 1 of the study. A.H. aided in conceiving and carrying out stage 2 of the study. G.B., G.C., A.K. and L.K. helped with study conception and feasibility. V.B., E.B., C.K. populated the Core Information Set. The Options Study Collaborative Group Members were contributors to the direction of the project and the final manuscript. All authors read and approved the final manuscript.

## Ethics Statement

A favourable ethical opinion for this study was granted on 27th April 2022 by the University of Bristol Research Ethics Committee (Ref: 10530).

## Consent

Consent was sought and obtained from participants in all parts of the study, including for the stakeholder survey, interviews, Delphi surveys and consensus meetings.

## Conflicts of Interest

The authors declare no conflicts of interest.

## Supporting information


**Appendix S1:** Data extraction form for studies.

**Appendix S2:** Data extraction form for patient information leaflets.

**Appendix S3:** Interview topic guide.

**Appendix S4:** Long‐list of information items.

**Appendix S5:** Vaginal birth core information set summary document.

**Appendix S6:** Vaginal birth core information set detailed document.

**Table S1:** Core Outcome Set‐STAndardised Protocol Items (COS‐STAP) Checklist.

**Table S2:** DELPHISTAR checklist.

**Table S3:** Pregnancy related demographics.

**Table S4:** Delphi survey round 1 scoring.

**Table S5:** Summary information item scoring for survey round 2 and inclusion or exclusion in consensus meeting.

**Table S6:** Information items included automatically in the core information set following the second Delphi survey round.

**Table S7:** Information on merging of information items.

**Table S8:** Voting on information items during consensus meetings and outcomes.

**Table S9:** Vaginal birth core information set.

**Table S10:** Supplementary list of information items with descriptions.

## Data Availability

All data collected for the study is under controlled access. Any access requests for data will be referred to the study committee for review on a case‐by‐case basis.
