# Supplementary material for: What Should Be Discussed When Considering a Vaginal Birth? A Delphi Consensus Study
Source: BJOG. 2025 Nov 18;133(3):520–31. doi: 10.1111/1471-0528.70071 (PMC12770075; doi:10.1111/1471-0528.70071)
Supplement: Supplementary file 1 — Appendix S1: Data extraction form for studies. [file BJO-133-520-s014.doc]

**S3.** Data extraction form for studies

**Identifiers**

| Review title or ID |  |
| --- | --- |
| Study ID *(surname of first author and year first full report of study was published e.g. Smith 2001)* |  |
| Report ID |  |
| Report ID of other reports of this study |  |
| Notes | |

# General information

| Date form completed *(dd/mm/yyyy)* |  |
| --- | --- |
| Name/ID of person extracting data |  |
| Reference citation |  |
| Study author contact details |  |
| Publication type  *(e.g. full report, abstract, letter)* |  |
| Notes: | |

# Study characteristics

| Type of study |  |
| --- | --- |
| Study setting |  |
| Study population |  |
| Details of intervention (if applicable) |  |
| Study methodology |  |

Information/Outcome

| Outcomes measured (verbatim) |  |
| --- | --- |
| Outcomes definitions (if stated) |  |
| Relevant outcome measure tool (if applicable); Is the tool validated for cultural context |  |
| Were parents and member of the public involved in outcome selection? |  |

.
