# Supplementary material for: What Should Be Discussed When Considering a Vaginal Birth? A Delphi Consensus Study
Source: BJOG. 2025 Nov 18;133(3):520–31. doi: 10.1111/1471-0528.70071 (PMC12770075; doi:10.1111/1471-0528.70071)
Supplement: Supplementary file 2 — Appendix S2: Data extraction form for patient information leaflets. [file BJO-133-520-s003.doc]

**S4.** Data extraction form for patient information leaflets

**Identifiers**

| Review title or ID |  |
| --- | --- |
| Leaflet ID *(name of leaflet, publisher and year first published published e.g. Waterbirth, RCOG, 2001)* |  |
| Notes | |

# General information

| Date form completed *(dd/mm/yyyy)* |  |
| --- | --- |
| Name/ID of person extracting data |  |
| Leaflet name |  |
| Source of leaflet (institute, trust etc.) |  |
| Notes: | |

# Leaflet information

| Leaflet subject area |  |
| --- | --- |
| Information setting (i.e. local, national) |  |

Information/Outcome

| Risks, complications, outcomes discussed (verbatim) |  |
| --- | --- |
| Risk/outcome definitions (if stated) |  |
