# Supplementary material for: What Should Be Discussed When Considering a Vaginal Birth? A Delphi Consensus Study
Source: BJOG. 2025 Nov 18;133(3):520–31. doi: 10.1111/1471-0528.70071 (PMC12770075; doi:10.1111/1471-0528.70071)
Supplement: Supplementary file 3 — Appendix S3: Interview topic guide. [file BJO-133-520-s013.docx]

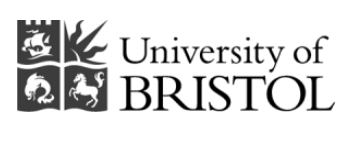
S3. Interview topic guide

Interview topic guide for antenatal/postnatal women

Introduction to interview:

The aim of this interview is to understand your thoughts on what information you think is important to be discussed when you are pregnant and planning to have a vaginal birth. We will ask about your plans for your labour or experiences you have had. We would also like to hear about your thoughts on what information is important in order to make sure we can have the right discussions with women who are having a baby.

Topic area 1: What are you currently planning for your labour and birth?

Prompts: Location, how, pain relief, the room, the people present

Topic area 2: Tell me about your labour experience/birth? [IF POSTNATAL]

Prompts: Location, analgesia, people in room, birth plan

Topic area 3: Have you received any information about labour or vaginal birth antenatally?

Prompts: Resources used, antenatal classes, websites.

Topic area 4: Where would you look for information on vaginal birth?

Topic area 5: Have you heard of any potential risks of vaginal delivery?

Prompts: Risks during, risks after, anecdotes/stories heard

Topic area 6: Was there anything about vaginal birth that you wish you’d been told before you had your baby? [IF POSTNATAL]

Prompts: Misleading information prior, surprising aspects to delivery

Topic area 7: What information do you think is most important to know before having a vaginal birth?

Prompts: risks during, risks afterwards, long term effects, people who are in attendance

Topic area 8: Do you think women should be consented for a vaginal birth?

Prompts: should there be a form, what should be on it, who would the discussion be with

Topic area 9: Did you experience anything in your labour which you think is important to know about prior to having a baby?

Prompts: complications, waiting times, pain, long-term effects

Topic area 10: What are the key information points to know about a vaginal birth, in your opinion?
