# Supplementary material for: What Should Be Discussed When Considering a Vaginal Birth? A Delphi Consensus Study
Source: BJOG. 2025 Nov 18;133(3):520–31. doi: 10.1111/1471-0528.70071 (PMC12770075; doi:10.1111/1471-0528.70071)
Supplement: Supplementary file 4 — Appendix S4: Long‐list of information items. [file BJO-133-520-s004.docx]

S6. Long list of information of information items used in Delphi Survey

How important is it for ***all*** women planning or considering a vaginal birth to know about...?

Section 1: Environment during labour

1. Keeping mobile and adopting different positions in labour
2. Information about being in water during labour and birth
3. Medical professionals who may be present in the room during labour
4. Labour companions who you can choose to have present during labour and their role in the process
5. Atmosphere and environment during labour
6. Items that may be needed when in labour
7. Eating and drinking in labour- What food or drink can be consumed; When can it be consumed or not?.
8. Birth locations: Choice of where to give birth (home, midwife led unit, consultant led unit)
9. Transfer of location during labour

Section 2: Labour process

1. The different stages of labour and birth
2. The signs and symptoms of labour
3. Expected progress during labour
4. What happens when waters break before labour
5. The effect a baby's position (which way the baby is facing) can have on labour and experience
6. Fluid intake during labour and urinating during labour
7. Giving birth to the placenta
8. Expected experiences whilst pushing during labour, when about to give birth
9. Expected experiences immediately following birth

Section 3: Pain relief during labour

1. Use of non-medical pain relief during labour
2. Use of simple medical pain relief during labour
3. Use of epidural during labour

Section 4: Possible labour complications

1. Moderate but common complications relating to the mother during labour
2. Moderate but common complications relating to the baby during labour
3. Severe but common complications related to vaginal bleeding during labour
4. Severe but uncommon complications relating to the baby during labour
5. Severe but rare complications for mother and baby during labour
6. Serious illness for mother that may result in a long-term hospital stay and/or possible long-term consequences (severe but very rare)
7. Maternal death (very rare)

Section 5: Possible procedures or interventions during labour

1. Vaginal examinations offered during labour
2. How a baby's wellbeing is monitored during labour
3. Procedures to investigate baby's wellbeing during labour when there are concerns with the monitoring
4. The process of speeding up labour (augmentation of labour)
5. When an assisted vaginal birth may be offered or recommended
6. When an episiotomy may be offered
7. When a caesarean section may be offered
8. When intravenous antibiotics may be recommended during labour
9. When a blood sample or drip (intravenous line) may be needed
10. Umbilical cord cutting
11. Methods to reduce risk of serious tears to the vagina

Section 6: Postnatal procedures

1. Examination of the vagina and the rectum following birth
2. Repair of tears with stitches
3. Manual removal of placenta
4. What is done when bleeding after birth is more than the usual
5. Transfer to different area of care due to concerns for mother's health

Section 7: Experiences immediately after birth (within hours)

1. Symptoms that may be experienced following birth
2. Pelvic floor injury that can happen during labour
3. Length of stay in unit or hospital following birth

Section 8: Experience in the days or weeks following birth

1. Retained tissue or placenta following birth
2. Issues with the perineum following birth
3. Maternal infection requiring antibiotics following birth
4. Bowel or bladder symptoms following birth
5. Serious maternal health conditions following birth that require medical treatment
6. Possible mental health experiences following vaginal birth

Section 9: Long-term experiences (months or years) following birth

1. Long term back pain
2. Future pregnancies and birth experiences
3. Pelvic floor and genital tract issues
4. Changes related to sexual health function after birth
5. Long term effects of childbirth on mental health
6. Effects of childbirth on social health

Section 10: Outcomes for the baby

1. Skin to skin following birth
2. Feeding of the baby following birth
3. Attachment of the baby following birth to the mother
4. Impact on baby's immune system
5. Condition of baby when they are born
6. Transmission of bloodborne viruses to baby
7. Length of hospital stay for the baby
8. Admission of baby to special care or neonatal intensive care unit (SCBU, NICU)
9. Birth trauma to baby during birth
10. Serious conditions that may affect baby in the short or long term
11. Physical impact on life of baby

Section 11: Wider effects of birth

1. Birth partner wellbeing following birth
2. Financial cost to family following birth
3. Hospital conditions can affect labour experience
4. Financial cost to health service

Section 12: Additional items added after first round of Delphi (based on participant suggestions)

1. Family planning following birth
2. Benefits of a vaginal birth compared to other modes of birth
3. After care in the immediate hours following a vaginal birth
