# Supplementary material for: What Should Be Discussed When Considering a Vaginal Birth? A Delphi Consensus Study
Source: BJOG. 2025 Nov 18;133(3):520–31. doi: 10.1111/1471-0528.70071 (PMC12770075; doi:10.1111/1471-0528.70071)
Supplement: Supplementary file 5 — Appendix S5: Vaginal birth core information set summary document. [file BJO-133-520-s008.pdf]

Birth Options

Vaginal Birth Core Information Set

What is a core information set?

A core information set is the information everyone needs before making a decision about their care. They do not replace personalised discussions. The Birth Options core information sets have been made for families and healthcare professionals to use to provide information to support decisions about birth. Women, birthing people, partners, midwives and doctors have decided which information is most important.

This information is intended as a guide and uses evidence from national guidelines, national statistics and research studies. It includes some more general information that explains usual practice. It has the best available information at the time that it was made (2025).

This is the Options Core Information set for vaginal birth

It includes information about **spontaneous vaginal birth**

It does not include extensive information about **induction of labour, instrumental vaginal birth, or caesarean birth.**

Other Core Information Sets are available for Induction of Labour, Elective Caesarean birth and Emergency Caesarean birth.

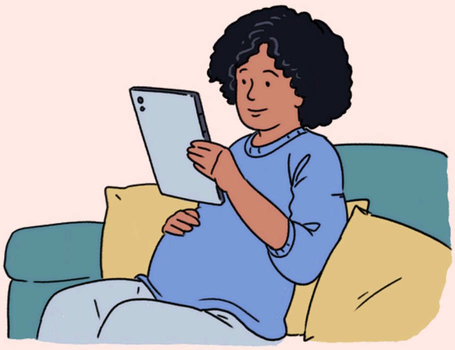

www.birthoptions.co.uk

FUNDED BY NIHR | National Institute for Health and Care Research

UNIVERSITY OF LIVERPOOL

NHS University Hospitals of Liverpool

University of BRISTOL

Birth Options

Vaginal Birth Core Information Set

The labour process

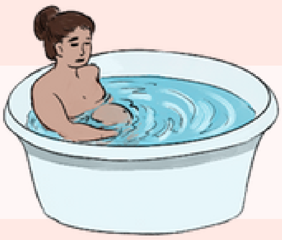

How the stages of labour are defined and the expected progress through these:

There are three stages of labour; each have different lengths for different people and pregnancies.

Stage 1 (Where the cervix opens up to 10cm) involves early contractions leading to regular ones, typically lasting around 8 hours for first-time mothers and about 5 hours for those who have given birth before.

Stage 2 (where you push and have your baby) can have a passive phase (where you are 10cm dilated but have not started to push yet) followed by an active phase, where pushing leads to the baby's birth. This lasts up to 5 hours for first-time mothers and up to 3 hours for those who have given birth before but can be much quicker.

Stage 3 (where you birth the placenta) involves the birth of the placenta, which usually occurs within 30 to 60 minutes.

Giving birth to the placenta

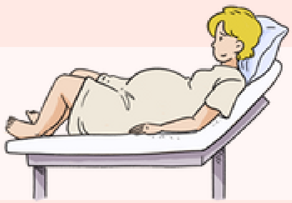

There are two approaches to birthing the placenta: active and physiological.

Active involves an injection to help birth the placenta, cutting the cord after the baby gets its blood from the cord, and then gently pulling the cord.

Physiological is without medications, waiting for the cord to stop beating before cutting, and pushing to birth the placenta.

FUNDED BY NIHR | National Institute for Health and Care Research
University of Liverpool
NHS University Hospitals of Liverpool
University of Bristol

Birth Options

Sometimes the placenta doesn't come without help, in this case medical intervention or an operation may be needed.

Active management lowers the risk of bleeding after birth but is more likely to lead to side effects like feeling sick, vomiting, and headache.

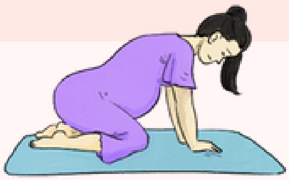

Expected experiences whilst pushing during labour, when about to give birth:

Sensations during pushing:

When you're pushing during labour, you might feel a lot of pressure downwards, like when you're trying to have a bowel movement. It is normal to feel sensations like passing gas or an intense stinging feeling, especially as the baby's head comes out.

Types of pushing:

There are different ways to push during labour:

- **Spontaneous**: pushing based on instincts.
- **Directed**: this is where your midwife encourages you to take a deep breath at the start of a contraction, hold it and push; taking further breaths when necessary.

Directed pushing can help shorten the pushing stage of labour, especially for first-time births, reducing the chance of needing a caesarean birth or instrumental vaginal birth.

Choice of where to give birth (home, midwife led unit, doctor led unit), and when & why may it be recommended to change location during labour:

Women give birth in different places: at home, in a midwife-led unit, a midwife-led unit in a hospital with a doctor/consultant-led unit, or in a consultant-led unit. Midwives, student midwives, and other healthcare students may be present in all settings.

FUNDED BY NIHR | National Institute for Health and Care Research
UNIVERSITY OF LIVERPOOL
NHS University Hospitals of Liverpool
University of BRISTOL

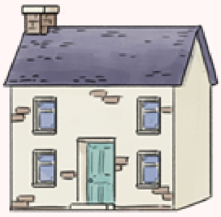

Home

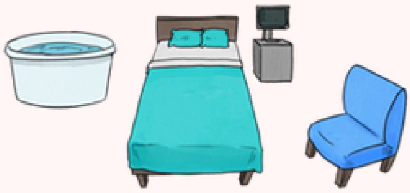

Midwife-led unit

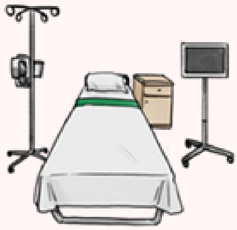

Consultant-led unit

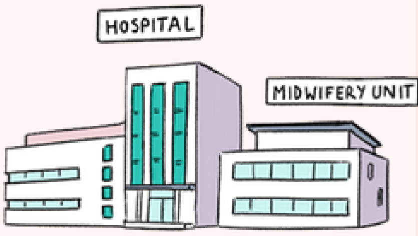

Alongside unit

Most care during labour is given by midwives, but certain things like epidurals and caesarean births are only available in consultant-led settings.

Recommendations for birth setting may be based on risk factors or previous complications. For low-risk pregnancies, the safety of all settings is similar. Please discuss your individual circumstances with your care-givers.

Giving birth at home or in a midwifery unit may reduce the likelihood of interventions like episiotomy or caesarean birth.

There is a chance that you may need to transfer from your chosen place of birth to a consultant-led unit. The rates of transfer into a consultant-led setting vary:

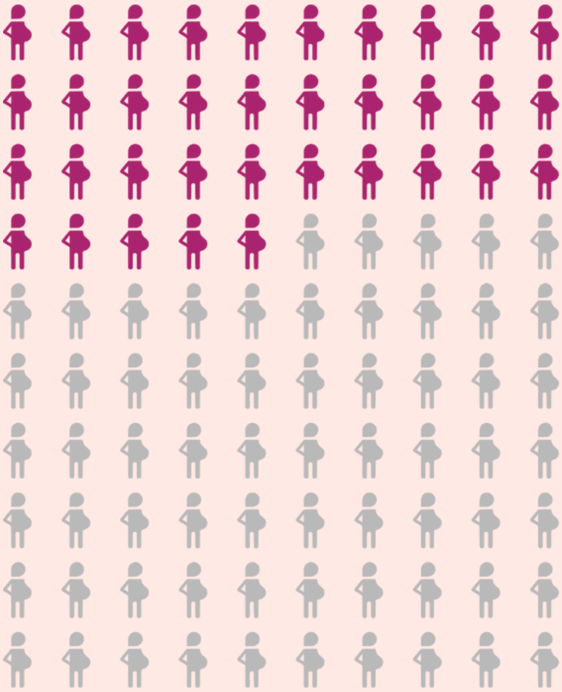

35 in 100

For first births with low-risk pregnancies, there's a 35-45 in 100 rate of transfer into a consultant-led unit.

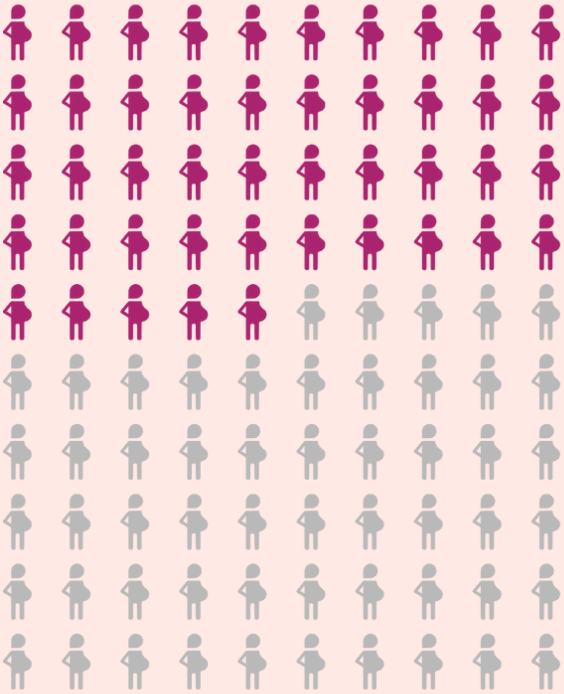

45 in 100

The time it takes to transfer from each birth setting will also vary.

FUNDED BY NIHR | National Institute for Health and Care Research
University of Liverpool
NHS University Hospitals of Liverpool
University of Bristol

For subsequent births with low-risk pregnancies, there's a 9–13 in 100 rate of transfer into a consultant-led unit.

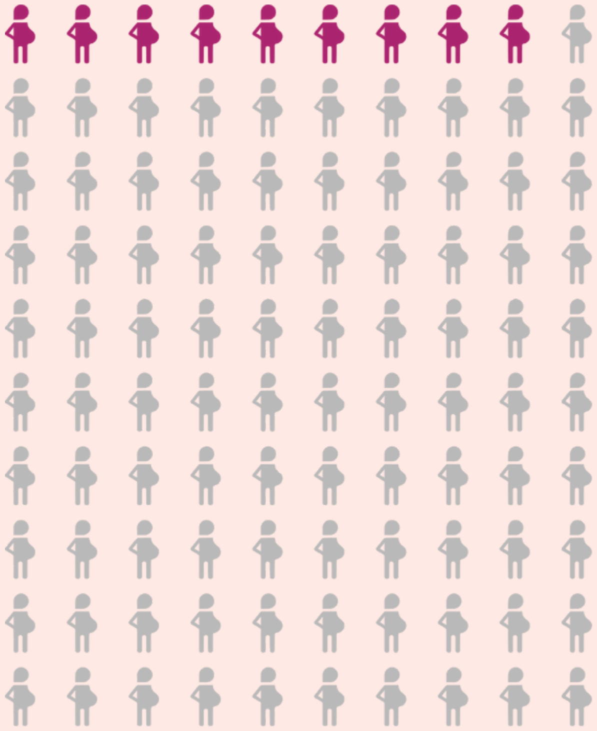

9 in 100

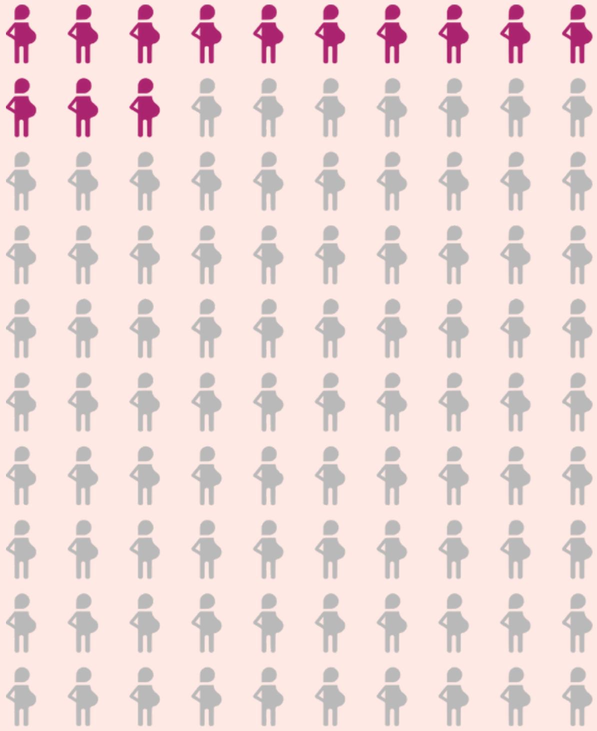

13 in 100

FUNDED BY NIHR | National Institute for Health and Care Research
UNIVERSITY OF LIVERPOOL
NHS Liverpool Women's NHS Foundation Trust
University of BRISTOL

Birth Options

Pain relief

Use of medical pain relief in labour including gas & air, oral medications (i.e. paracetamol, dihydrocodeine), injectable medications (i.e. pethidine, diamorphine) & epidural

Paracetamol and Dihydrocodeine tablets provide relief for hours but may take some time to work.

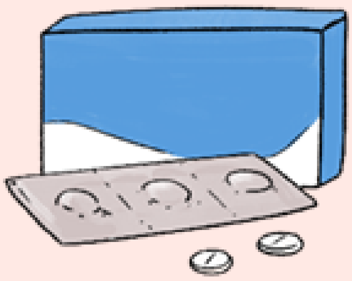

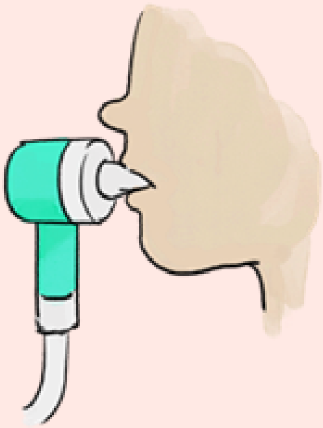

Gas and Air (Entonox) is a quick and effective pain relief option available in all settings, though it may cause light-headedness and sickness. The effects also wear off quickly.

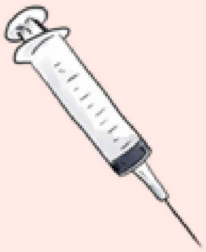

Injected medications like diamorphine and pethidine take 20-30 minutes to work, may have side effects for both the mother and baby, including drowsiness and difficulty breastfeeding.

Epidurals, administered through the back, offer effective pain relief, do not slow down cervical dilatation, but may prolong pushing time and increase the likelihood of instrumental birth.

Remifentanil, administered through a vein through a cannula, reduces the need for epidurals and instrumental birth but may increase the baby's risk of respiratory distress.

Additional Supportive Medication:

Anti-sickness medication can be provided to manage sickness or vomiting

Non-medical Pain Relief Methods are used by many women.

FUNDED BY NIHR | National Institute for Health and Care Research
UNIVERSITY OF LIVERPOOL
NHS University Hospitals of Liverpool
University of BRISTOL

Environment during labour

Labour companions who you can choose to have present during labour and their role in the process.

- Having a birth partner can mean you have a better birth experience.

- How many birth partners you have with you can vary between hospitals and areas in the hospital.

- You can choose any adult you wish to be your birth partner.

Keeping mobile and adopting different positions during labour

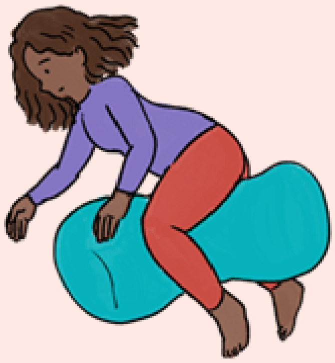

You can choose positions that make you feel comfortable and are right for you. Lying on your side may increase the chance of a spontaneous vaginal birth.

Your choice of position may also be affected by factors such as having an epidural.

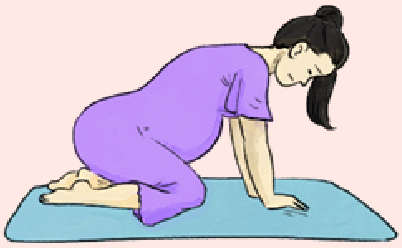

Sometimes you may be asked to lie in a position to help monitor your baby or to make sure your baby is getting enough oxygen.

Birth Options

Possible procedures or interventions during labour

Vaginal examinations offered during labour:

Vaginal examinations are offered to check how open the cervix is (neck of the womb), see if the waters are broken, break waters and to see if the baby is coping with labour. You can choose whether to have these examinations and can request a chaperone.

How often vaginal examinations are offered will depend on each person. Examinations may occur every 4 hours during the first stage of labour and more frequently during the second stage to monitor the baby's position and progress.

Post-birth, an examination will be offered if the placenta does not come out by itself. An examination will be offered after birth to check for tears and that your anus (back passage) is intact.

How baby's wellbeing is checked during labour monitoring and procedures:

During labour, intermittent or continuous monitoring can be used.

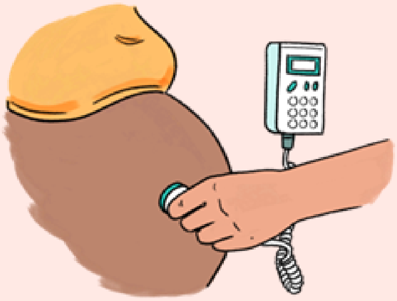

Intermittent monitoring means your midwife checks your baby's heartbeat with a handheld doppler.

Continuous electronic fetal monitoring watches your baby's heartbeat and contractions

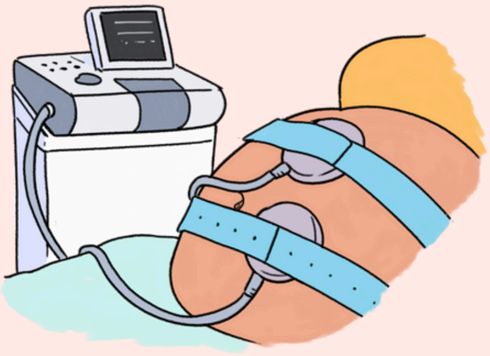

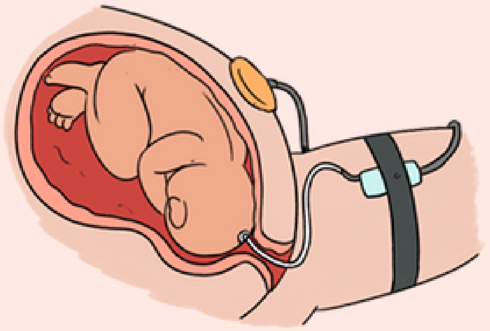

A fetal Scalp Electrode is a monitoring device placed on the baby's head for continuous monitoring through your abdomen or with a clip (fetal scalp electrode) on your baby's head.

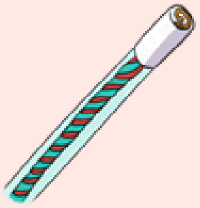

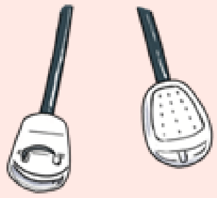

Different types of fetal scalp electrode

Both intermittent and continuous monitoring have similar outcomes. Continuous monitoring may reduce seizures in babies, though it's linked to more caesarean and instrumental births.

If during continuous monitoring, the baby's heartbeat looks normal, it is a good indication that the baby is getting enough oxygen. If it looks abnormal, there is a chance that the baby could be in distress, but we cannot be certain about this.

Therefore, we may recommend further tests to check on baby's wellbeing.

- A vaginal examination and gently stimulating the baby's head
- A small blood sample from the baby's head

If these tests are reassuring, labour can continue. If they are not reassuring, you may be offered an urgent birth (either by caesarean or instrumental birth).

FUNDED BY NIHR | National Institute for Health and Care Research
UNIVERSITY OF LIVERPOOL
NHS University Hospitals of Liverpool
University of BRISTOL

Birth Options

Methods to reduce the risk of serious tears to the perineum:

Perineum refers to the area between the vagina and anus.

Most women in their first births will have a graze or tear. A more serious tear (those involving the anus), occurs in 6 in 100 first births and approximately 2 in 100 subsequent births.

Some tears will need stitches, and some can heal themselves.

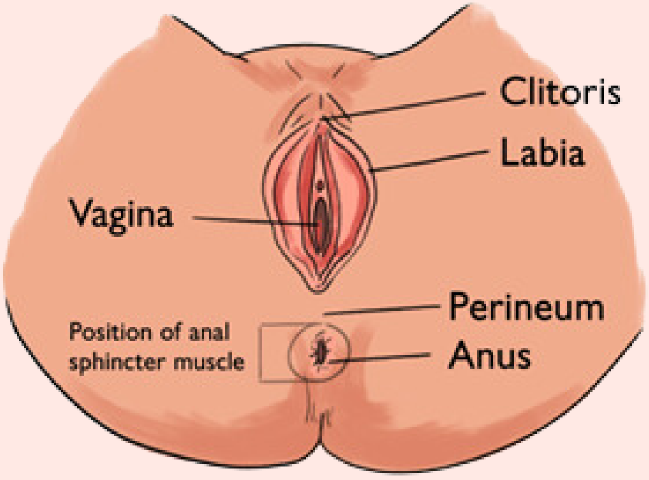

Guided pushing, massage/warm compress, and support of the perineum during birth can reduce the risk of more serious tears.

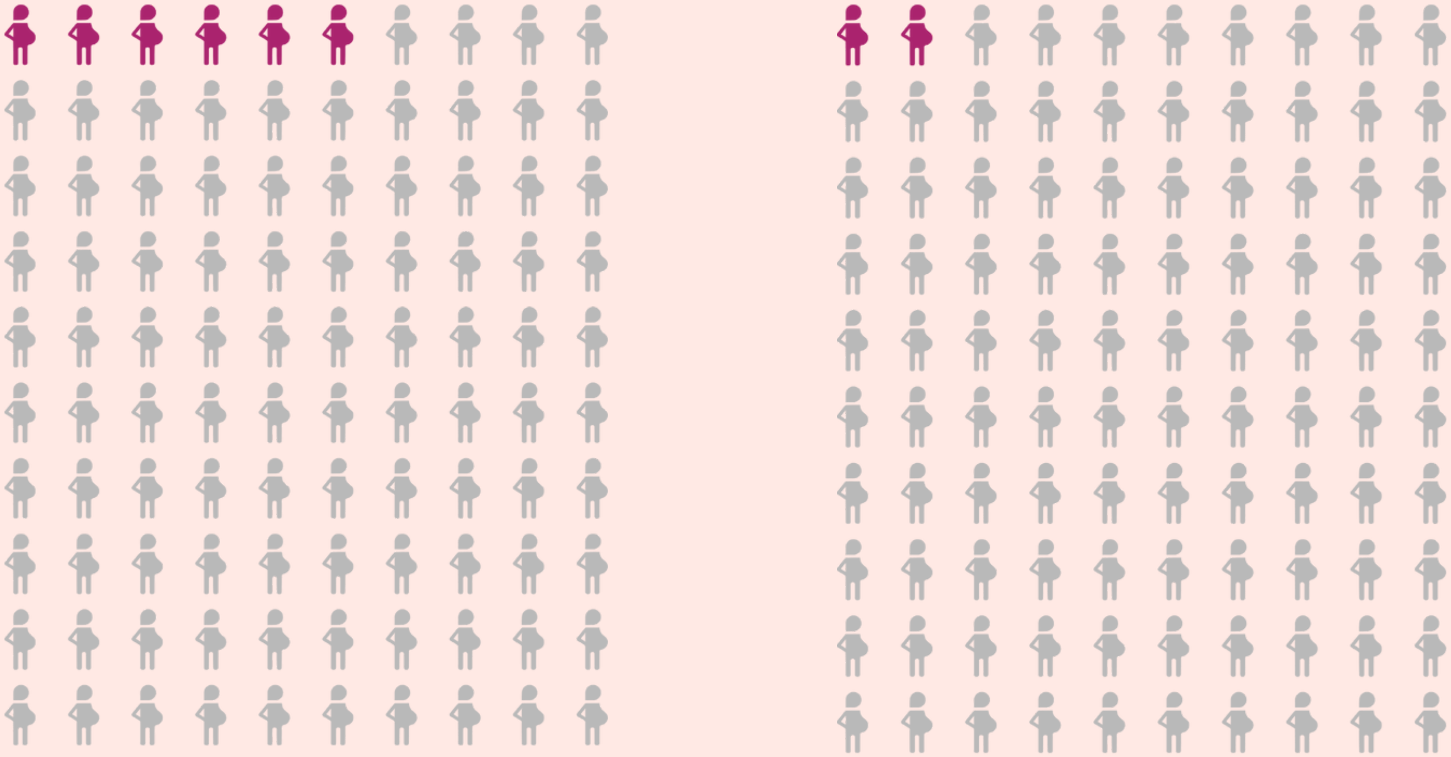

The most serious tears happen in 6 in 100 first births

The most serious tears happen in 2 in 100 subsequent births

FUNDED BY NIHR | National Institute for Health and Care Research
University of Liverpool
NHS University Hospitals of Liverpool
University of Bristol

When Episiotomy may be offered:

Episiotomy is a small cut made at the vaginal opening to assist birth
It is not routine but is typically recommended if there is a risk of a more serious perineal tear or in urgent situations like an instrumental vaginal birth.

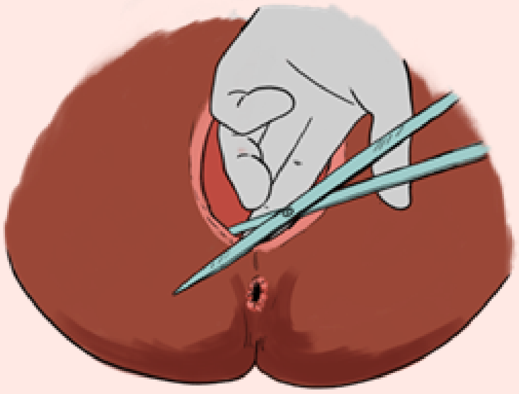

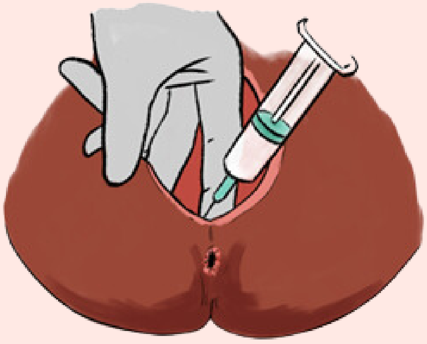

It aims to reduce the severity of perineal tears or assist birth.
Local anaesthetic (a numbing agent) is injected into the area, and you will require stitches after birth.

When an Instrumental or caesarean birth may be offered or recommended and why:

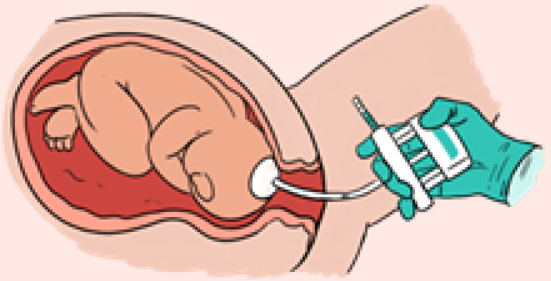

Around **18 in 100** women experience instrumental vaginal birth during their first birth, and **5 in 100** during subsequent births

Reasons include delay during birth, maternal request, or concerns about the baby.

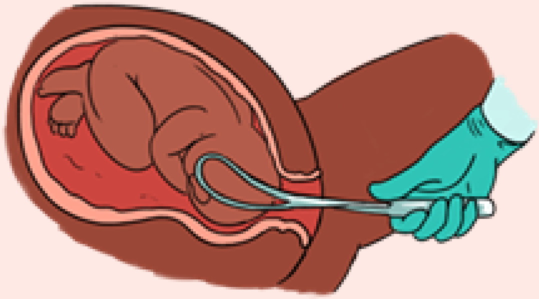

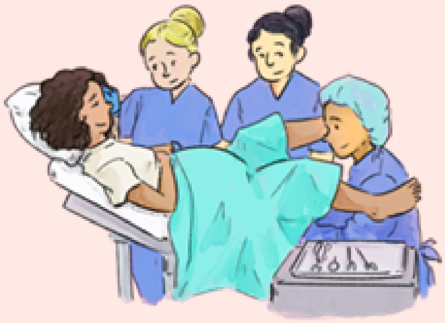

FUNDED BY NIHR National Institute for Health and Care Research
University of Liverpool
NHS University Hospitals of Liverpool
University of Bristol

Birth Options

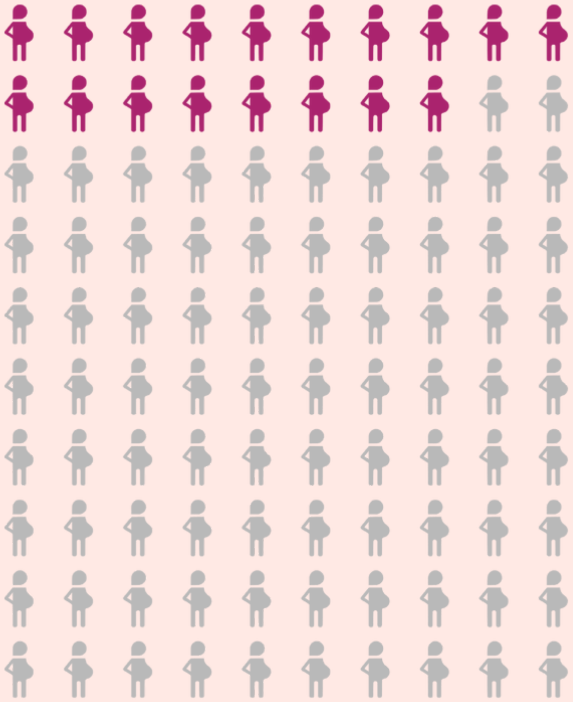

18 in 100 women had an instrumental vaginal birth for their first birth

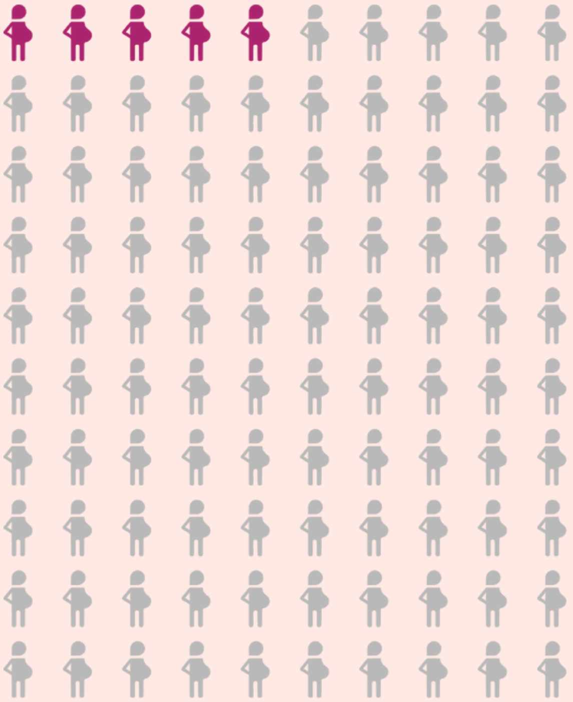

5 in 100 women had an instrumental vaginal birth in a subsequent birth

If an instrumental birth is not possible or does not work, a caesarean birth may be offered. Caesarean birth involves the baby being born through a cut to the abdomen and uterus (womb). This is typically performed in a surgical theatre with anaesthesia (numbing agent) administered to prevent pain in the lower part of your body.

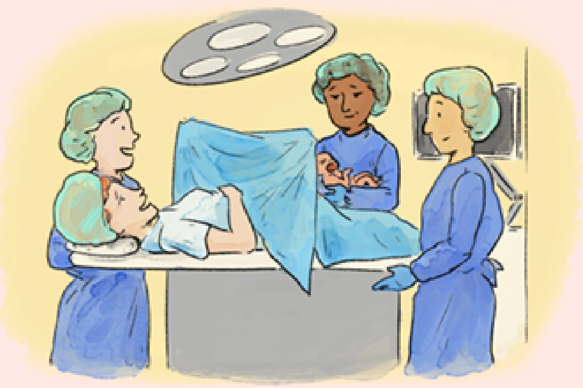

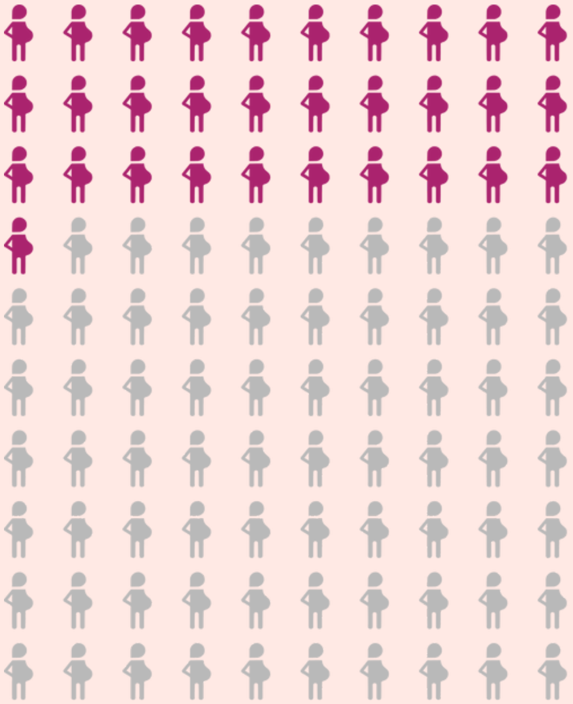

31 in 100 women had an urgent caesarean birth for their first birth

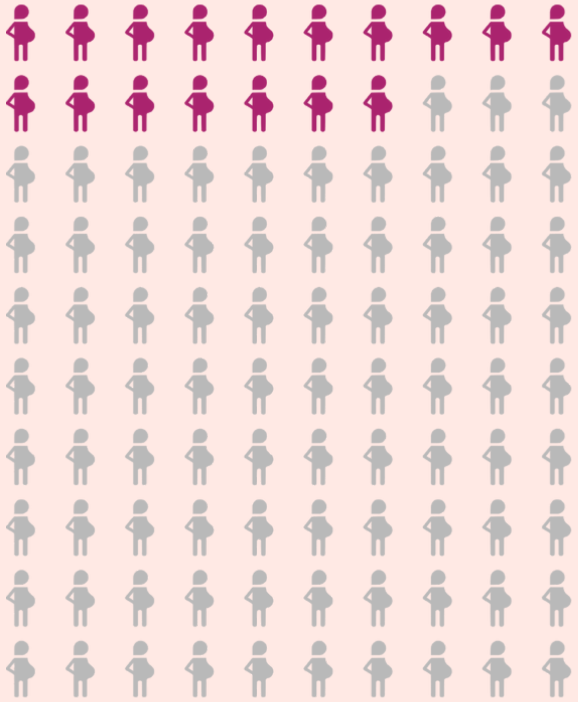

17 in 100 women had an urgent caesarean birth for a subsequent birth

31 in 100 women in 2023-2024 had an urgent caesarean birth in their first pregnancy and **17 in 100** women in subsequent pregnancies.

FUNDED BY NIHR | National Institute for Health and Care Research
University of Liverpool
NHS University Hospitals of Liverpool
University of Bristol

Possible labour complications

Complications relating to the mother during labour:

High maternal temperature, happens in 10 in 100 women, it may require blood tests, swabs, antibiotics, and fluids.

Raised blood pressure may require blood tests, urine samples, and medication.

Uterine hyperstimulation, where the uterus (womb) contracts too frequently. It can cause your baby to not get enough oxygen. We can treat this by stopping any medication that is used to help your labour to progress, and/or giving you a medication to relax your uterus but an urgent birth may still be needed.

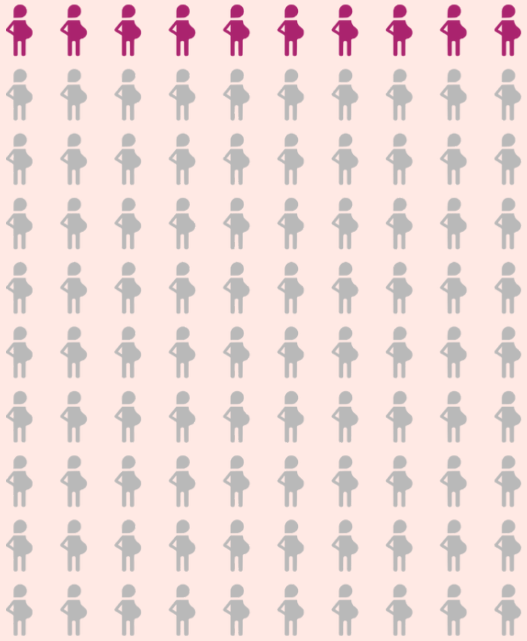

High maternal temperature happens in 10 in 100 women

Bleeding before or during labour is a complication that requires urgent review and if we are concerned about you or your baby, we may recommend urgent birth.

Monitoring vital signs like blood pressure, heart rate, and urine output during labour helps identify issues early on.

Complications relating to the baby:

A baby's first poo (meconium) may happen before birth and may occur more commonly in post-dates (41 weeks+) pregnancies. This can indicate distress in some cases. Breathing in meconium can make the baby unwell. Transfer to a consultant-led unit is needed for monitoring and specialised care.

Fetal distress (baby may not be getting enough oxygen) may mean you are offered an urgent birth.

FUNDED BY NIHR | National Institute for Health and Care Research
University of Liverpool
NHS University Hospitals of Liverpool
University of Bristol

Birth Options

Shoulder dystocia: (Baby's shoulder gets stuck after the head is out) happens in fewer than 1 in 100 (0.58-0.70 in 100) vaginal births. This can lead to brain or shoulder injuries. Injury involving the shoulder and arms (brachial plexus injury) occurs in 4 in 10,000 births, 10% of these injuries are permanent.

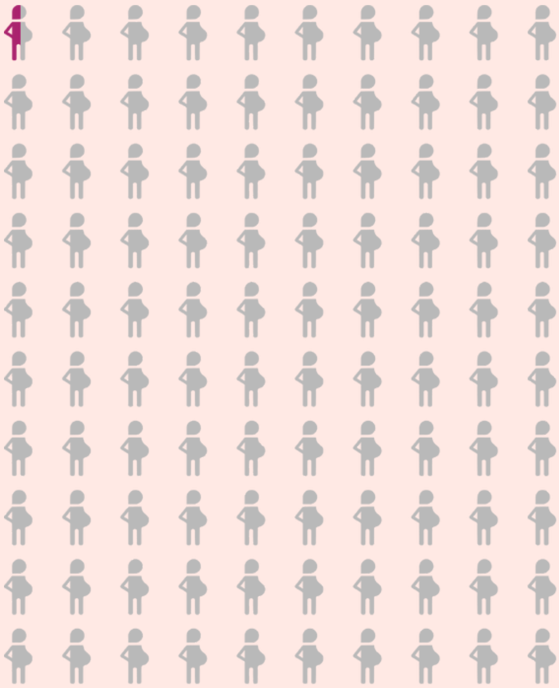

0.58 in 100

Shoulder dystocia happens in fewer than 1 in 100 (0.58-0.70 in 100) vaginal births.

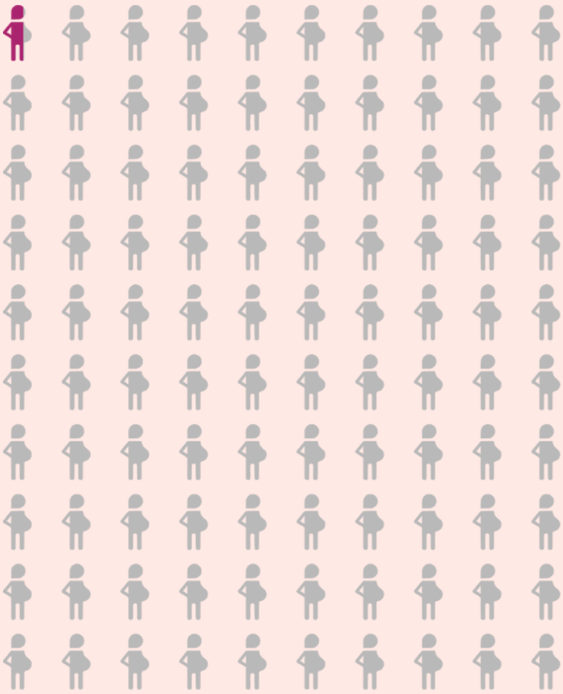

0.70 in 100

Uterus (womb) infection (chorioamnionitis) affecting both the mother and the baby, requires antibiotics. The risk is higher if waters break before labour starts compared to intact waters.

Risk of your baby developing infection is 1 in 100 if your waters break before labour starts vs. 0.5 in 100 if your labour starts and your waters are intact.

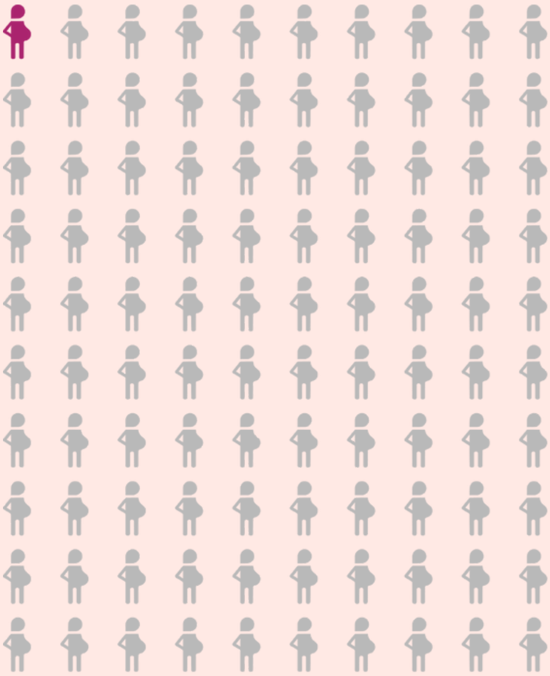

If your waters break before labour starts, the risk of your baby developing an infection is 1 in 100

FUNDED BY NIHR National Institute for Health and Care Research
University of Liverpool
NHS University Hospitals of Liverpool
University of Bristol

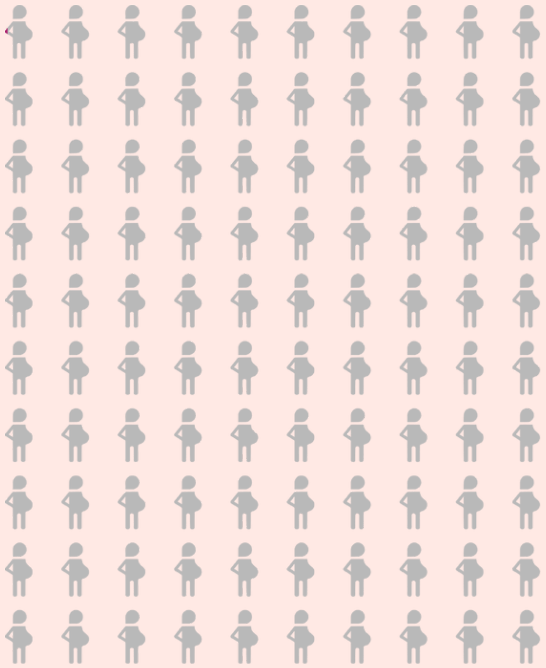

0.1 in 100

Cord prolapse happens in **0.1–0.6 in 100** births

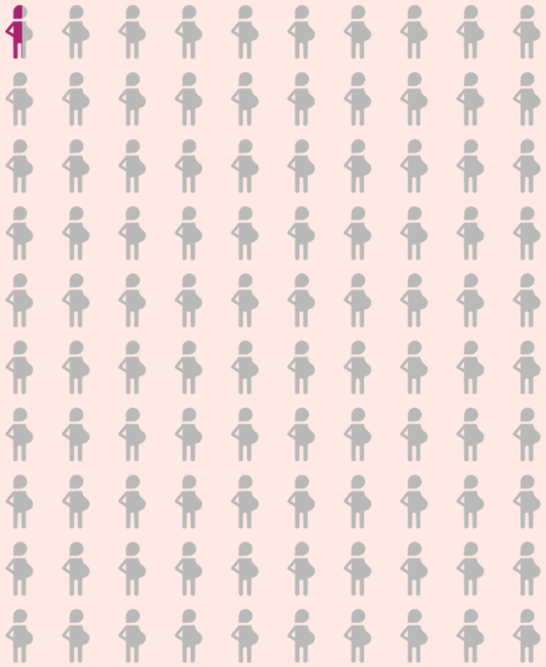

0.6 in 100

A Cord Prolapse (umbilical cord coming out before the baby) is rare (0.1–0.6 in 100). Immediate birth is necessary if this happens.

Outcomes for the baby

Condition of baby when they are born

Immediately after birth, an assessment called the APGAR test will be done to see if your baby needs more support.

If your baby has difficulty breathing or has a low heart rate at birth, they may need immediate help to breathe. This may involve encouraging breathing or helping the baby's heart to pump blood around their body.

The baby may need to be taken to another location (e.g. neonatal unit) for further care and tests.

FUNDED BY NIHR | National Institute for Health and Care Research
University of Liverpool
NHS University Hospitals of Liverpool
University of Bristol

Experiences after birth

Possible experiences or symptoms immediately after birth

It is common to experience pain and discomfort. This can include cramping similar to period pains (after pains), bruising, and perineal pain.

Bleeding, known as lochia, is normal and can last up to 12 weeks. If you are soaking through pads and passing large clots, this is not normal. You should seek medical assistance.

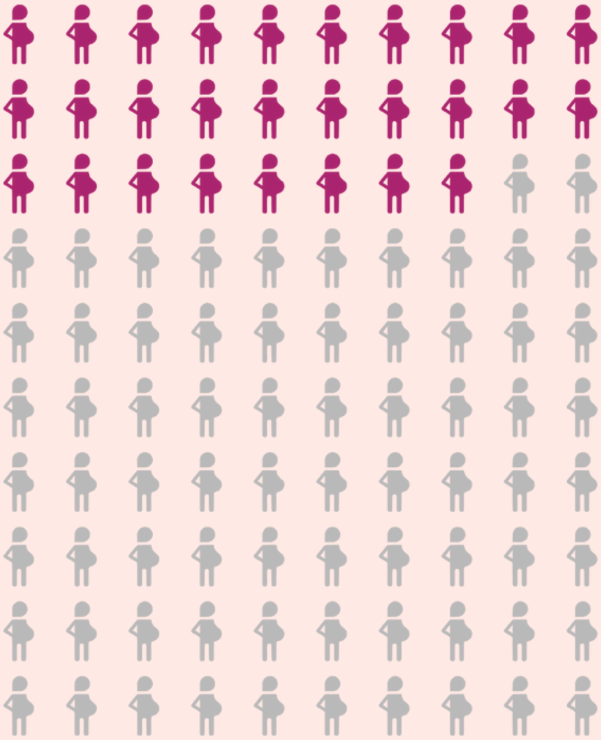

Around 28 in 100 people will experience urinary incontinence of any type following a spontaneous vaginal birth

Physical symptoms like urinary incontinence or difficulty urinating may occur, especially after certain types of labour. Around 28 in 100 people will experience urinary incontinence in the first 2 weeks following a spontaneous vaginal birth.

Emotionally, you might feel anxious, exhausted, or down, which is normal in the first week or two. If these feelings last longer it's important to seek help from a midwife or GP.

FUNDED BY NIHR National Institute for Health and Care Research
University of Liverpool
NHS University Hospitals of Liverpool
University of Bristol

Feeding the baby following birth

There are three options for feeding your baby. These are breastfeeding, formula and a mix of both (combi feeding).

You will be encouraged to start to feed within an hour of birth where possible. Your midwives and doctors will support whichever decision you make regarding the type of milk you choose for your baby.

We recommend breastfeeding if possible as there are health benefits even if you breastfeed for a short time.

Breastfeeding is not right for everyone and there are lots of different factors. If you would like to/need to use formula milk, you can use the first infant formula for the whole first year of your baby's life. To prepare feeds safely, bottles should be sterilised.

Pelvic floor injuries that can happen during labour, examination of the area to assess these, and potential issues with this area following birth:

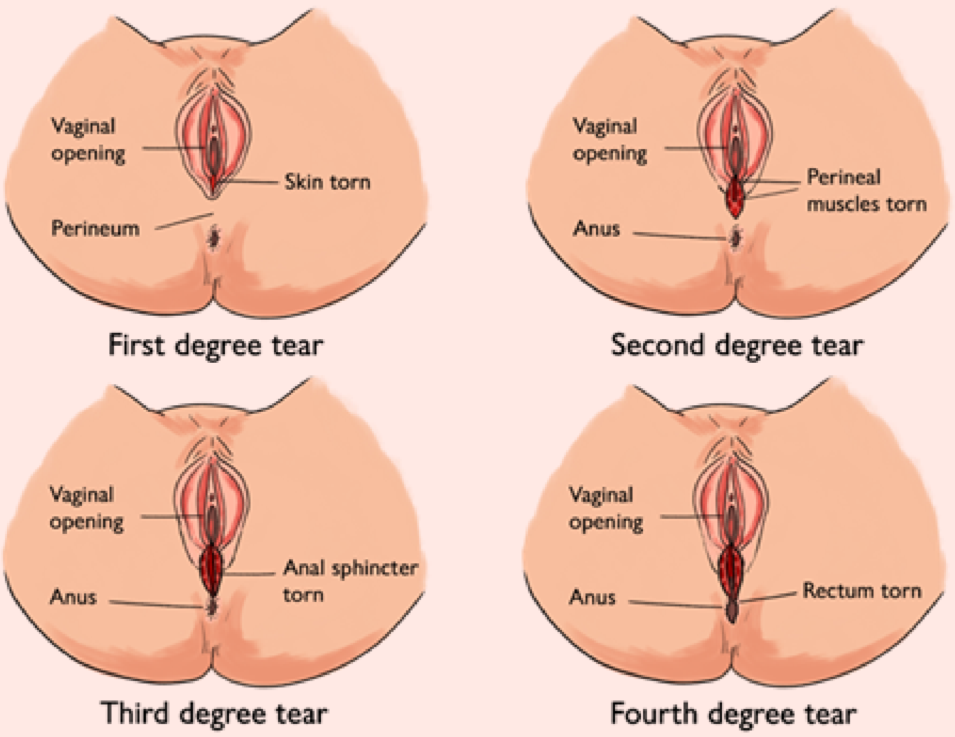

Pelvic floor injuries range from minor grazes to more serious tears involving the anus and rectum.

1st degree tears involve the skin, and often don't require stitches, while 2nd degree tears involving the perineal muscles do.

More serious tears (3rd and 4th degree) involving the anus or rectum require stitches, antibiotics and physiotherapy.

Symptoms following a tear may include pain, changes in sensation, pain during sexual intercourse, infections, bladder or bowel issues (urinary or faecal incontinence), which might be temporary or long-lasting.

60-80 out of 100 women have no symptoms a year after experiencing the most serious tears.

Mental health problems like body image issues and self-esteem concerns can arise, regardless of physical symptoms.

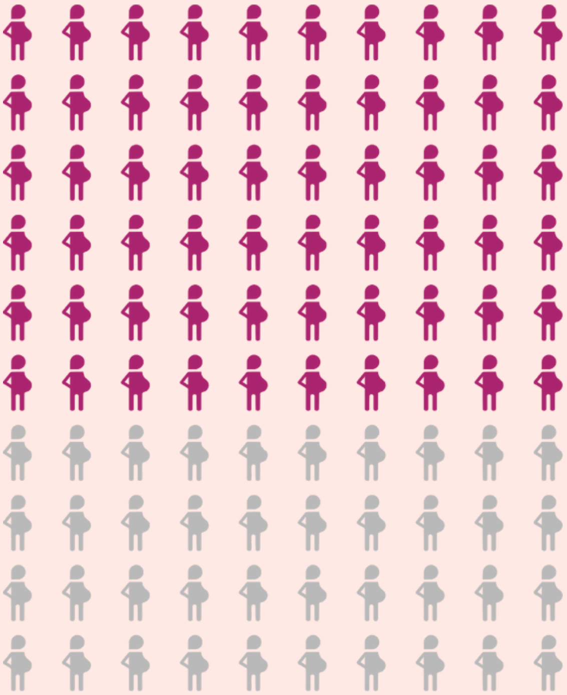

60 out of 100

60-80 out of 100 women with the most serious tears have no symptoms a year later

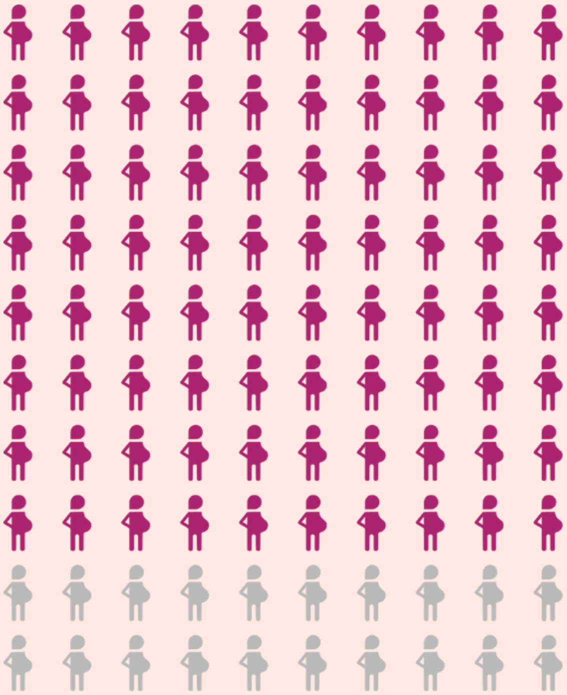

80 out of 100

Possible mental health experiences following birth (may be in the short and long term):

Mental health issues following birth are common and effect each woman differently. Baby blues, (30-80 out of 100 women), causes mood swings, anxiety, and irritability, and usually fades within two weeks.

FUNDED BY NIHR | National Institute for Health and Care Research
University of Liverpool
NHS University Hospitals of Liverpool
University of Bristol

Baby blues happen in 30 – 80 out of 100 women

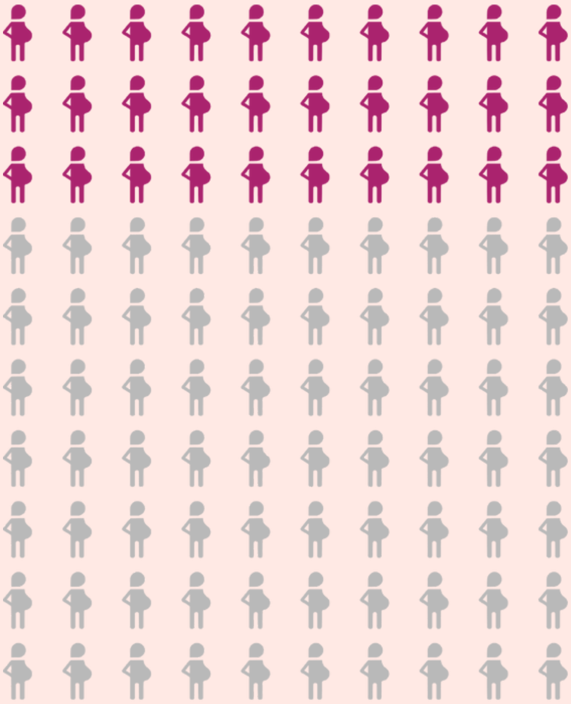

30 out of 100

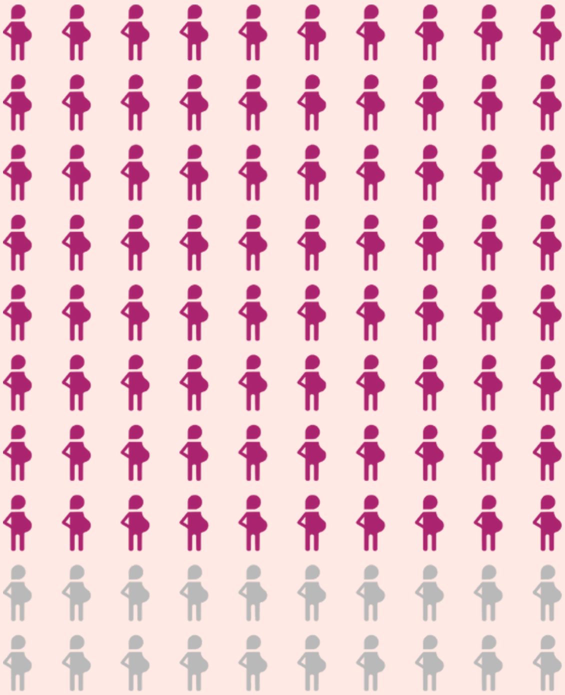

80 out of 100

Postnatal depression, (15-20 out of 100 women), may involve persistent sadness, loss of interest, and physical symptoms, and can start in the first year after birth.

Postnatal depression happens in 15-20 in 100 women

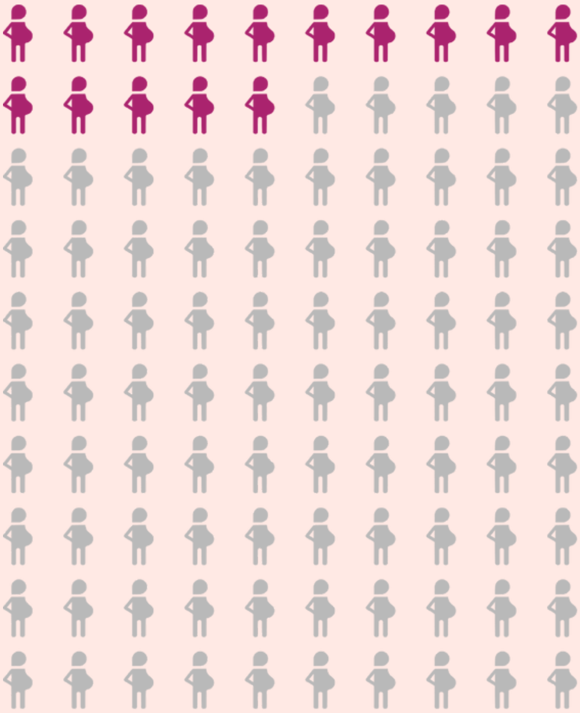

15 out of 100

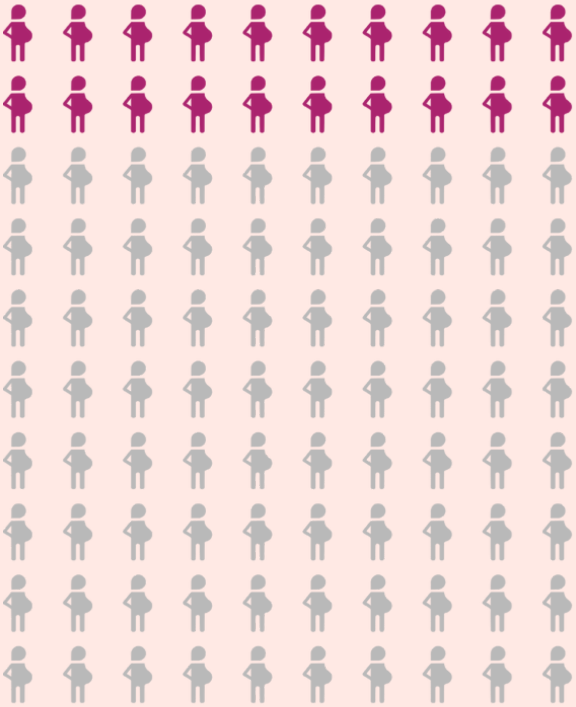

20 out of 100

FUNDED BY NIHR | National Institute for Health and Care Research
UNIVERSITY OF LIVERPOOL
NHS University Hospitals of Liverpool
University of BRISTOL

Birth Options

Postnatal anxiety (15-20 out of 100 women), may include worrying thoughts that keep happening, feelings of panic or panic attacks, constant restlessness, irritability and feeling tense.

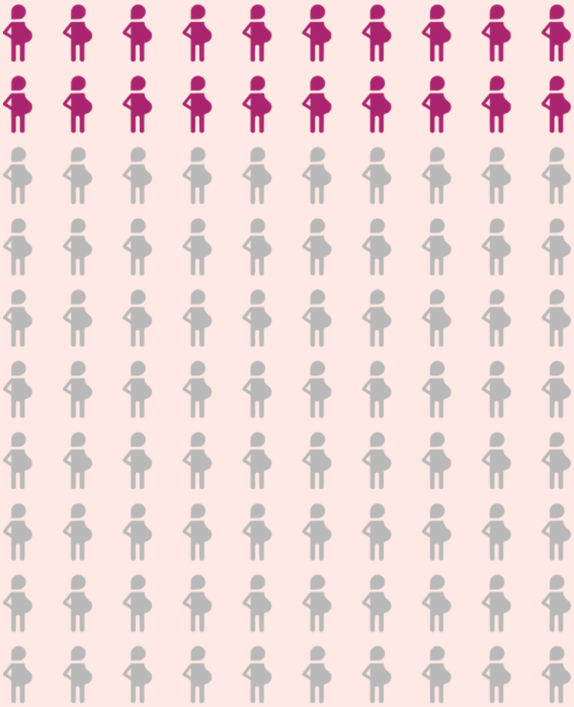

15 out of 100

Postnatal anxiety happens in 15-20 in 100 women

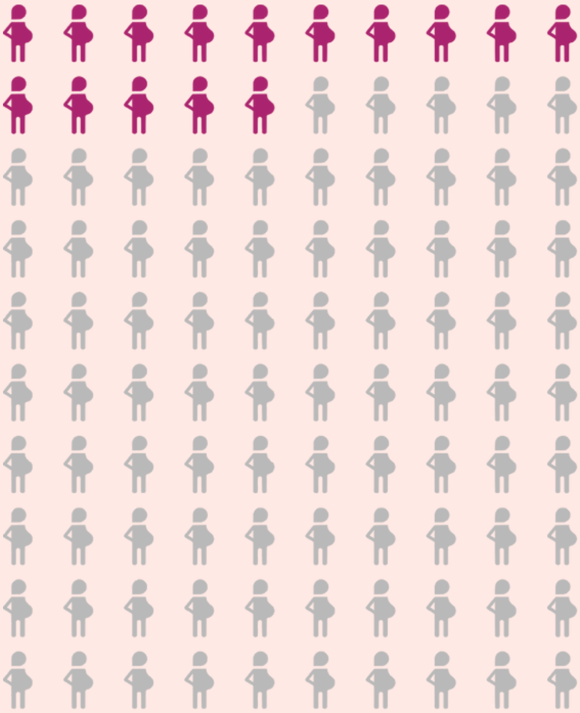

20 out of 100

Post-traumatic stress disorder (PTSD) can happen after a traumatic event when the negative feelings don’t go away. These feelings can interfere with daily life.

Post-partum psychosis is rare (0.1 in 100 women) and tends to start within two weeks of birth. It includes extreme mood changes, paranoia, and altered behaviour, often necessitating immediate intervention.

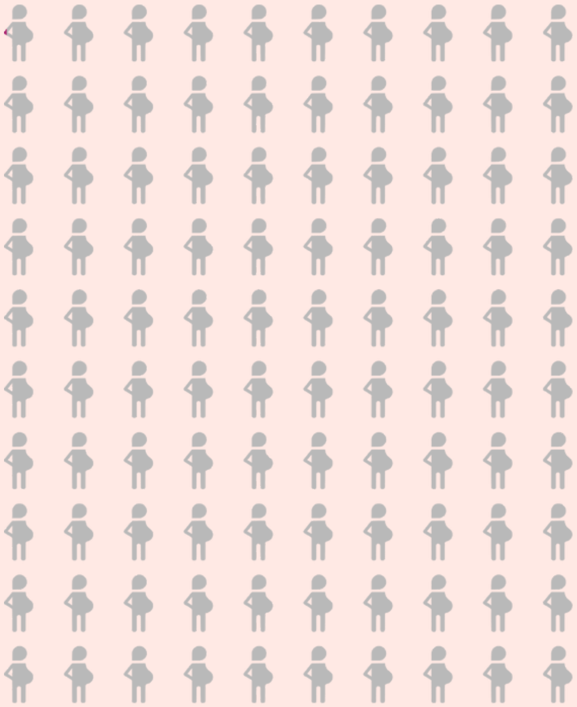

Postpartum psychosis happens in 0.1 in 100 women

If you are worried about any of these symptoms, please contact your midwife, health visitor, 111 or GP.

FUNDED BY NIHR | National Institute for Health and Care Research
UNIVERSITY OF LIVERPOOL
NHS University Hospitals of Liverpool
University of BRISTOL

Supplementary information for if you decide to have a vaginal birth

The signs and symptoms of labour

Contractions might begin like intense period pains and are different from earlier Braxton-Hicks contractions as they are longer. Lower back pain and a feeling of heaviness or achiness. The mucus plug, a jelly-like pink substance, may come out of your vagina, indicating that the cervix (neck of the womb) is starting to open. Your waters can break before or during labour, either as a slow trickle or a sudden gush.

Waters breaking before labour

If your water breaks before labour, contact your midwife or maternity unit. You should contact your maternity unit immediately if there is any bleeding.

You will be offered induction straight away or at 24 hours if you prefer. 60 in 100 labours start naturally within 24 hours of the waters breaking. Waiting too long means there is a higher risk of serious infection for your baby.

You can continue to have baths and showers as normal as this is not known to increase your risk of infection, however, sexual intercourse after your waters have broken may increase infection risk.

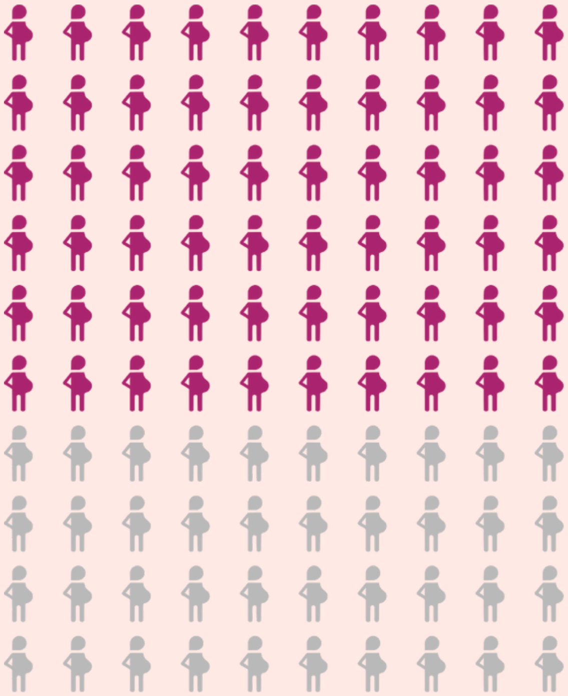

60 in 100 labours will start naturally within 24 hours of the waters breaking

FUNDED BY NIHR National Institute for Health and Care Research
UNIVERSITY OF LIVERPOOL
NHS University Hospitals of Liverpool
University of BRISTOL

Medical professionals who may be present at your labour

During your labour there may be midwives, doctors (obstetricians and anaesthetists), midwifery care assistants, student midwives and student doctors present. Who is present will depend on where you are having your baby. How many will be present depends on your birth and how it is going. Whether you have students present is your choice, and you will be asked beforehand.

Food and drink during labour

During labour, you will have the freedom to eat and drink, although there may be recommendations for a light diet for some women. There's advantage to consuming more fluids than usual, isotonic drinks might be preferable to water. However, personal medical conditions need to be considered, so please talk to your midwives and doctors.

Skin-to-skin

Skin-to-skin involves placing the baby directly onto either the mother's or birthing partner's bare skin following birth. Skin-to-skin is encouraged as soon as possible, as it is beneficial for both the mother and the baby, promotes breastfeeding and helps with bonding.

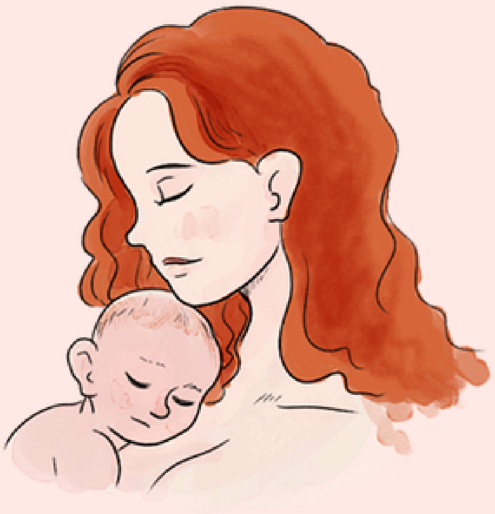

FUNDED BY NIHR | National Institute for Health and Care Research
UNIVERSITY OF LIVERPOOL
NHS University Hospitals of Liverpool
University of BRISTOL

Birth Options

Where we got this information from

The Labour Process

Intrapartum care. NICE guidelines Published September 29, 2023. Accessed October 18, 2023. https://www.nice.org.uk/guidance/ng235/chapter/Recommendations#planning-place-of-birth

Hollowell J, Rowe R, Townend J, et al. The Birthplace in England national prospective cohort study: further analyses to enhance policy and service delivery decision-making for planned place of birth. Health Serv Deliv Res. 2015;3(36).

Pushing methods for the second stage of labour. doi:10.1002/14651858.CD009124.pub3

Chapman V, Charles C. The Midwife's Labour and Birth Handbook. Newark: John Wiley & Sons, Incorporated; 2013

Assisted vaginal birth (ventouse or forceps). Royal College of Obstetricians and Gynaecologists. https://www.rcog.org.uk/for-the-public/browse-our-patient-information/assisted-vaginal-birth-ventouse-or-forceps/

Environment During Labour

Intrapartum care. NICE guidelines Published September 29, 2023. Accessed October 18, 2023. https://www.nice.org.uk/guidance/ng235/chapter/Recommendations#planning-place-of-birth

Fetal monitoring in labour. NICE guidelines. Published December 14, 2022. Accessed October 20, 2023. https://www.nice.org.uk/guidance/ng229

Assisted vaginal birth (ventouse or forceps). Royal College of Obstetricians and Gynaecologists. https://www.rcog.org.uk/for-the-public/browse-our-patient-information/assisted-vaginal-birth-ventouse-or-forceps/

Pushing methods for the second stage of labour. doi:10.1002/14651858.CD009124.pub3

Bohren MA, Hofmeyr GJ, Sakala C, Fukuzawa RK, Cuthbert A. Continuous support for women during childbirth. CDSR. 2017 July 16;(7).

Mobbs N, Williams C, Weeks A. Humanising birth: Does the language we use matter? The BMJ. Published February 8, 2018. Accessed November 28, 2023. https://blogs.bmj.com/bmj/2018/02/08/humanising-birth-does-the-language-we-use-matter

Possible Labour Complications

Intrapartum care. NICE guidelines Published September 29, 2023. Accessed October 18, 2023. https://www.nice.org.uk/guidance/ng235/chapter/Recommendations#planning-place-of-birth

Chandraharan E, Krishna A. Diagnosis and management of postpartum haemorrhage. BMJ. 2017;358:j3875. doi:10.1136/bmj.j3875

Intrapartum care for women with existing medical conditions or obstetric complications and their babies. Evidence reviews for pyrexia. NICE. Published online 2019.

Calman KC, Royston G. Personal paper: Risk language and dialects. British Medical Journal 1997;315:939–42.

Crofts J, Draycott TJ et al, on behalf of the Royal College of Obstetricians and Gynaecologists. Shoulder Dystocia. BJOG 2012
Chebsey CS, Fox R, et al on behalf of the Royal College of Obstetricians and Gynaecologists. Umbilical Cord Prolapse. BJOG 2014

Pain Relief

Intrapartum care. NICE guidelines Published September 29, 2023. Accessed October 18, 2023. https://www.nice.org.uk/guidance/ng235/chapter/Recommendations#planning-place-of-birth

Twin and Triplet pregnancy | Guidance | NICE. Published September 4, 2019. Accessed November 24, 2023. https://www.nice.org.uk/guidance/NG137

Opiate injections. Newcastle Hospitals NHS Foundation Trust. Published February 5, 2021. Accessed January 8, 2024. https://www.newcastle-hospitals.nhs.uk/services/maternity/labour-and-birth/pain-relief/opiate-injections/

Possible procedures or interventions during labour

Intrapartum care. NICE guidelines Published September 29, 2023. Accessed October 18, 2023. https://www.nice.org.uk/guidance/ng235/chapter/Recommendations#planning-place-of-birth

Fetal monitoring in labour. NICE guidelines. Published December 14, 2022. Accessed October 20, 2023. https://www.nice.org.uk/guidance/ng229

Alfirevic Z, Devane D, Gyte GML, Cuthbert A. Continuous cardiotocography (CTG) as a form of electronic fetal monitoring (EFM) for fetal assessment during labour. Cochrane Database of Systematic Reviews 2017, Issue 2. Art. No.: CD006066. DOI: 10.1002/14651858.CD006066.pub3

Bohren MA, Hofmeyr GJ, Sakala C, Fukuzawa RK, Cuthbert A. Continuous support for women during childbirth. CDSR. 2017 July 16;(7).

Mobbs N, Williams C, Weeks A. Humanising birth: Does the language we use matter? The BMJ. Published February 8, 2018. Accessed November 28, 2023. https://blogs.bmj.com/bmj/2018/02/08/humanising-birth-does-the-language-we-use-matter/

Signs that labour has begun. nhs.uk. Published December 1, 2020. Accessed November 20, 2023. https://www.nhs.uk/pregnancy/labour-and-birth/signs-of-labour/signs-that-labour-has-begun/

Preterm labour and birth | Guidance | NICE. Published November 20, 2015. Accessed November 24, 2023. https://www.nice.org.uk/guidance/ng25

NHS Maternity Statistics, England, 2023-24. last accessed 10/03/25. Available from: https://digital.nhs.uk/data-and-information/publications/statistical/nhs-maternity-statistics/2023-24/births

Episiotomy. Royal College of Obstetricians and Gynaecologists. https://www.rcog.org.uk/for-the-public/perineal-tears-and-episiotomies-in-childbirth/episiotomy/

Fernado RJ, Sultan AH, et al on behalf of the Royal College of Obstetricians and Gynaecologists. The Management of Third-and-Fourth-Degree Perineal Tears. BJOG 2015

Murphy DJ, Strachan BK, Bahl R, on behalf of the Royal College of Obstetricians Gynaecologists. Assisted Vaginal Birth. BJOG 2020;127:e70–e112.
Caesarean birth. NICE guidelines. Published March 31, 2021. Accessed October 20, 2023. https://www.nice.org.uk/guidance/ng192

NMPA Project Team. National Maternity and Perinatal Audit: Clinical Report 2022. Based on births in NHS maternity services in England and Wales between 1 April 2018 and 31 March 2019. London: RCOG; 2022.

Assisted vaginal birth (ventouse or forceps). Royal College of Obstetricians and Gynaecologists. https://www.rcog.org.uk/for-the-public/browse-our-patient-information/assisted-vaginal-birth-ventouse-or-forceps/

Wyn Jones N, Mitchell EJ, Wakefield N, et al. Impacted fetal head during second stage Caesarean birth: A prospective observational study. European Journal of Obstetrics & Gynecology and Reproductive Biology. 2022;272:77-81. doi:10.1016/j.ejogrb.2022.03.004

Perineal tears and episiotomy. NHS inform. Accessed December 4, 2023. https://www.nhsinform.scot/ready-steady-baby/labour-and-birth/assisted-birth/perineal-tears-and-episiotomy/

FUNDED BY NIHR | National Institute for Health and Care Research
University of Liverpool
NHS University Hospitals of Liverpool
University of Bristol

Where we got this information from

Outcomes for the Baby

Intrapartum care. NICE guidelines Published September 29, 2023. Accessed October 18, 2023. https://www.nice.org.uk/guidance/ng235/chapter/Recommendations#planning-place-of-birth

Birthplace in England research programme | sheer | NPEU. Accessed October 18, 2023. https://www.npeu.ox.ac.uk/birthplace

Fetal monitoring in labour. NICE guidelines. Published December 14, 2022. Accessed October 20, 2023. https://www.nice.org.uk/guidance/ng229
Quality statement 7: Skin-to-skin contact |

Intrapartum care | Quality standards | NICE. Published December 10, 2015. Accessed October 20, 2023. https://www.nice.org.uk/guidance/qs105/chapter/quality-statement-7-skin-to-skin-contact

Postnatal care of the baby | Guidance | NICE. Published April 20, 2021. Accessed November 20, 2023. https://www.nice.org.uk/guidance/ng194/chapter/Recommendations#postnatal-care-of-the-baby

Lee AC, Cousens S, Wall SN, et al. Neonatal resuscitation and immediate newborn assessment and stimulation for the prevention of neonatal deaths: a systematic review, meta-analysis and Delphi estimation of mortality effect. BMC Public Health. 2011;11(3):S12. doi:10.1186/1471-2458-11-S3-S12

Supplementary information

Intrapartum care. NICE guidelines Published September 29, 2023. Accessed October 18, 2023. https://www.nice.org.uk/guidance/ng235/chapter/Recommendations#planning-place-of-birth

Eating and drinking in labour. doi:10.1002/14651858.CD003930.pub3

https://www.nice.org.uk/guidance/ng235/chapter/Recommendations#planning-place-of-birth Quality statement 7: Skin-to-skin contact | Intrapartum care | Quality standards | NICE. Published December 10, 2015. Accessed October 20, 2023. Chapman V, Charles C. The Midwife's Labour and Birth Handbook. Newark: John Wiley & Sons, Incorporated; 2013

Signs that labour has begun. nhs.uk. Published December 1, 2020. Accessed November 20, 2023. https://www.nhs.uk/pregnancy/labour-and-birth/signs-of-labour/signs-that-labour-has-begun/

How we present information about risk

Very common	1 in 1 to 1 in 10
Common	Less than 1 in 10 to 1 in 100
Uncommon	Less than 1 in 100 to 1 in 1000
Rare	Less than 1 in 1000 to 1 in 10,000
Very rare	Less than 1 in 10,000

Experiences After Birth

Murphy DJ, Strachan BK, Bahl R, on behalf of the Royal College of Obstetricians Gynaecologists. Assisted Vaginal Birth. BJOG 2020;127:e70–e112.

Intrapartum care. NICE guidelines Published September 29, 2023. Accessed October 18, 2023. https://www.nice.org.uk/guidance/ng235/chapter/Recommendations#planning-place-of-birth
Early days. nhs.uk. Published December 8, 2020. Accessed November 20, 2023. https://www.nhs.uk/pregnancy/labour-and-birth/after-the-birth/early-days/

Fernado RJ, Sultan AH, et al on behalf of the Royal College of Obstetricians and Gynaecologists. The Management of Third-and-Fourth-Degree Perineal Tears. BJOG 2015

Postnatal care - Formula feeding information and support. https://www.nice.org.uk/guidance/ng194/evidence/t-formula-feeding-information-and-support-pdf-326764486011#:~:text=Breastfeeding%20is%20known%20to%20have,breast%20cancer%20in%20the%20mothers

Postnatal care of the baby | Guidance | NICE. Published April 20, 2021. Accessed November 20, 2023. https://www.nice.org.uk/guidance/ng194/chapter/Recommendations#postnatal-care-of-the-baby

Episiotomy. Royal College of Obstetricians and Gynaecologists. https://www.rcog.org.uk/for-the-public/perineal-tears-and-episiotomies-in-childbirth/episiotomy/

Depression - antenatal and postnatal: What else might it be? NICE. Accessed October 21, 2023. https://www.nice.org.uk/cks-uk-only

Antenatal and postnatal mental health: clinical management and service guidance | Guidance | NICE. Published December 17, 2014. Accessed November 24, 2023. https://www.nice.org.uk/guidance/cg192

Assisted vaginal birth (ventouse or forceps). Royal College of Obstetricians and Gynaecologists. https://www.rcog.org.uk/for-the-public/browse-our-patient-information/assisted-vaginal-birth-ventouse-or-forceps/
Caesarean birth. NICE guidelines. Published March 31, 2021. Accessed October 20, 2023. https://www.nice.org.uk/guidance/ng192

Postnatal care | Guidance | NICE. Published April 20, 2021. Accessed November 24, 2023. https://www.nice.org.uk/guidance/ng194

Pelvic floor dysfunction: prevention and non-surgical management | Guidance | NICE. Published December 9, 2021. Accessed November 24, 2023. https://www.nice.org.uk/guidance/ng210/chapter/Recommendations

Depression - antenatal and postnatal. NICE. Accessed November 20, 2023. https://www.nice.org.uk/cks-uk-only

Postnatal depression. nhs.uk. Published February 15, 2021. Accessed November 20, 2023. https://www.nhs.uk/mental-health/conditions/post-natal-depression/overview/

Postnatal depression | Royal College of Psychiatrists. www.rcpsych.ac.uk. Accessed November 20, 2023. https://www.rcpsych.ac.uk/mental-health/mental-illnesses-and-mental-health-problems/post-natal-depression

Postpartum psychosis for carers | Royal College of Psychiatrists. www.rcpsych.ac.uk. Accessed November 20, 2023. https://www.rcpsych.ac.uk/mental-health/mental-illnesses-and-mental-health-problems/postpartum-psychosis-in-carers

Antenatal care | Guidance | NICE. Published August 19, 2021. Accessed November 24, 2023. https://www.nice.org.uk/guidance/ng201

Urinary retention. NICE. Accessed November 20, 2023. https://www.nice.org.uk/bnf-uk-only

FUNDED BY NIHR | National Institute for Health and Care Research
University of Liverpool
NHS University Hospitals of Liverpool
University of Bristol
