# Supplementary material for: What Should Be Discussed When Considering a Vaginal Birth? A Delphi Consensus Study
Source: BJOG. 2025 Nov 18;133(3):520–31. doi: 10.1111/1471-0528.70071 (PMC12770075; doi:10.1111/1471-0528.70071)
Supplement: Supplementary file 6 — Appendix S6: Vaginal birth core information set detailed document. [file BJO-133-520-s010.pdf]

Vaginal Birth Core Information Set

Birth Options

This is the Options Core Information set for vaginal birth

It includes information about spontaneous vaginal birth

It does not include extensive information about induction of labour, instrumental vaginal birth, or caesarean birth.

Other Core Information Sets are available for Induction of Labour, Elective Caesarean birth, Emergency Caesarean birth and Instrumental birth.

The Options Study | Institute of Life Course and Medical Sciences | University of Liverpool

FUNDED BY NIHR | National Institute for Health and Care Research

University of Liverpool

NHS Liverpool Women's NHS Foundation Trust

University of Bristol

Birth Options

The labour process

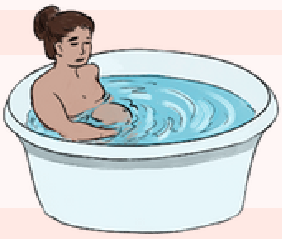

How the stages of labour are defined and the expected progress through these:

There are three stages to labour.
Labour progresses through these stages at **different rates for different women** and we will consider factors such as how many babies you have had and whether you have had an epidural when making recommendations about your progress. If there is a delay in any of these stages, we may offer to carry out interventions to try and speed your labour up.

Stage 1 is made of three parts. During this stage of labour, you will go from experiencing some contractions to having regular contractions. In your **first birth** this is expected to last **on average 8 hours** and **not usually more than 18 hours**. If you have already had a baby, this part is expected to last on average 5 hours and unlikely to last over 12 hours. **We will say there is a delay in the first stage of labour if your cervix opens less than 2cm in a 4-hour period.**

The **early stages** are where you may experience some contractions and your cervix will start to change. This part continues until your cervix is 4cm dilated.

The **'active' stage**– this is where your **cervix is actively dilates from 4cm to 10cm** and you are **contracting regularly**. Some women experience an intensity at the end of this stage. This is known as a **'transition'.** This is where the cervix is fully dilated (10cm) but you are not regularly pushing. Labour and birth are very individual and not everyone will experience these stages in the same way.

Stage 2 is made up of two parts, the passive and active stage. In **first births**, we expect this stage to take up to **3 hours** and in subsequent births we expect this to take up to 2 hours. We would not expect you to need to push for more than 2 hours.
·**The passive stage**. During this part you will have a fully dilated cervix but you will not have started to push yet.
·**The active stage** is where you will be pushing and baby will be born.

FUNDED BY NIHR National Institute for Health and Care Research
University of Liverpool
NHS Liverpool Women's NHS Foundation Trust
University of Bristol

Stage 3 is the stage where your placenta is born. You can have an **'active'** or a **'physiological'** third stage. An active third stage is where you are offered an injection to encourage your placenta to be born, and we speed its birth by pulling on the cord.
A physiological third stage will be where we don't give you an injection and do not pull on the cord.
We recommend an 'Active' third stage to reduce the risk of bleeding after birth.
The third stage of labour is prolonged if it is not complete within 30 minutes after birthing your baby if we use active management, and 60 minutes if we use physiological management.

Interventions to speed up first stage of labour:
Breaking your waters: If your waters have not broken or we see that your 'membranes' are intact still (fluid from baby), we may offer to break them manually
.
Oxytocin: This medication goes into a vein which we can access by putting a cannula in your arm or hand. This medication is given to try and help increase the number and strength of your contractions. If you would like this, we have to keep an eye on your baby a bit more closely and will use continuous monitoring which involves having some straps on your abdomen which are attached to a machine.

Oxytocin doesn't have an impact on the mode of birth you will end up with but it can cause hyperstimulation. This is where your uterus contracts too much and it can increase the risk of transient fetal hypoxia which is where your baby has less oxygen for a short period. If there are issues with this, we can stop the oxytocin. If you decide that you would like to have the oxytocin.

Interventions to speed up second stage of labour:
There are two interventions: **oxytocin** to increase the strength and frequency of contractions or an **instrumental vaginal birth** or if this is not possible a **caesarean section.**

Giving birth to the placenta

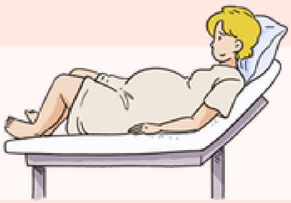

Giving birth to the placenta happens after your baby has been born. There are two approaches to this: physiological or active management.

FUNDED BY NIHR | National Institute for Health and Care Research
UNIVERSITY OF LIVERPOOL
NHS Liverpool Women's NHS Foundation Trust
University of BRISTOL

Active management is where we give you an injection to help the placenta come out of your uterus, we clamp and cut the cord after approximately two minutes and we pull gently on the cord to encourage the placenta to be born quickly. We recommend this to reduce excessive bleeding after birth.

Physiological management is where we don't use drugs, we only clamp the cord after it has stopped pulsating or if the placenta has been delivered. The placenta is delivered by your effort rather than us guiding it out.

You are **less likely to have a post-partum haemorrhage (big bleed after birth) when we do active management** compared to physiological management.

You are also **less likely to need a blood transfusion, b**ecome anaemic, or need more uterotonic drugs to help. However, you are more likely to feel sick, be sick, have a headache, have high blood pressure, and come back to hospital with bleeding.

If your placenta doesn't come out or it is taking too long, we can give you drugs to help it be born, empty your bladder and perform a vaginal examination. It this does not work; we will need to transfer you to a surgical theatre for a procedure to take your placenta out of your uterus. If you are not in a consultant-led unit, this will mean transferring you to one.

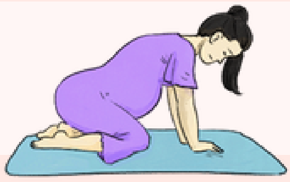

Expected experiences whilst pushing during labour, when about to give birth:

During pushing you may experience a **variety of sensations.**

During labour there is a lot of pressure pushing down below and you will be bearing down like you are trying to have a bowel movement. As a result, you may open your bowels and may pass wind, both of which are completely normal. You may also experience a stinging sensation down below: This can happen as your baby's head is coming out, or if you are tearing.

The types of pushing are:
Spontaneous- where women follow their own instincts and push around 3-5 times per contraction
Directed- done during the second stage of labour. You take a deep breath at the beginning of the contraction and then push throughout the contraction.
Delayed directed- where you are asked to avoid pushing until there is an irresistible urge to push, or if part of baby has moved down into the perineum.
Directed pushing can shorten the length of the pushing stage (stage 2) of labour and for first births can reduce chance of unplanned caesarean birth, and for subsequent births can reduce chance of an instrumental vaginal birth.

FUNDED BY NIHR National Institute for Health and Care Research
University of Liverpool
NHS Liverpool Women's NHS Foundation Trust
University of Bristol

Choice of where to give birth (home, midwife led unit, doctor led unit), and when & why may it be recommended to change location during labour:

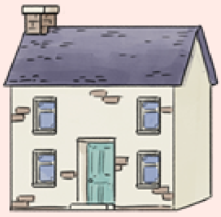

Home

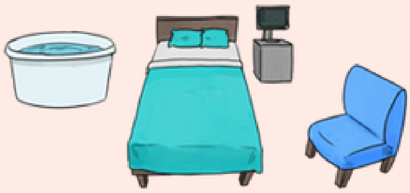

Midwife-led unit

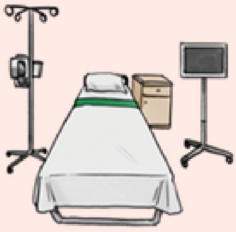

Consultant-led unit

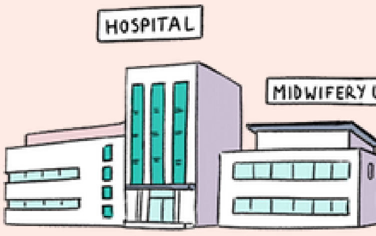

Alongside unit

Women give birth in different places such as at home, in a unit run by midwives either alone or nearby one run by doctors, or in a doctor-led unit. In all settings there will be midwives, student midwives and other healthcare students including student doctors. Most care during labour is given by midwifes in all settings, however, there are certain procedures which are only available in a doctor-led setting, for example, epidural, instrumental vaginal birth, caesarean birth, management of infection and other urgent surgical interventions.

Your midwife or doctor may recommend having your baby in a specific setting. For example, if you are at an increased risk of or have previously experienced labour complications, they may recommend having your baby in a midwifery unit in a hospital with a doctor-led unit, or in a doctor-led delivery suite. In these doctor-led settings, there will be paediatric doctors (doctors that look after babies and children) and anaesthetic doctors available in these areas as well if you need any help from them.

Different locations have different risks and benefits for you and your baby.

If this is your first or if you have already had a baby, giving birth at home or in a freestanding midwifery unit might mean intervention is less likely and vaginal birth is more likely. Interventions include episiotomy, caesarean section and assisted vaginal birth. If you are giving birth at home, there is a small increase in the number of babies who will have a serious medical problem, in all other locations the outcomes for babies are the same.

FUNDED BY NIHR National Institute for Health and Care Research
UNIVERSITY OF LIVERPOOL
NHS Liverpool Women's NHS Foundation Trust
University of BRISTOL

If you have had a baby before, giving birth at home or in a freestanding midwifery unit might mean intervention is less likely and vaginal birth is more likely. Interventions include episiotomy, caesarean section and assisted vaginal birth. The outcome for your baby at home or in a freestanding midwifery unit is no different than their outcome if you gave birth in a consultant-led unit.

An important consideration if you decide to give birth at home, or in a midwifery led unit is the rate of transfer into a consultant-led setting. Transfers can be at your request (e.g. for an epidural for pain relief) or because of an emergency (e.g. concerns with baby's heart rate, bleeding after birth), or because of a change of situation in labour (e.g. baby has done a poo inside your womb and causing them distress, a doctor is required to fix your vaginal tear).

If this is your first birth and your pregnancy is considered low risk, there is an **35-45 in 100** rate of transfer into an consultant-led unit.

If this is a subsequent birth and your pregnancy is considered low risk there is an **9-13 in 100** rate of transfer into an doctor-led unit. It is useful for you to consider this, and the length of time it takes for a transfer in from each of the various birth settings.

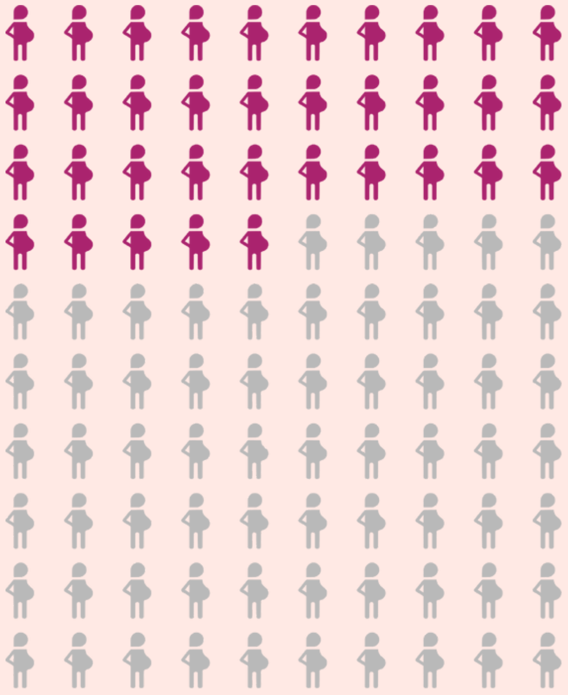

35 in 100

For first births with low-risk pregnancies, there's a 35-45 in 100 rate of transfer into a consultant-led unit.

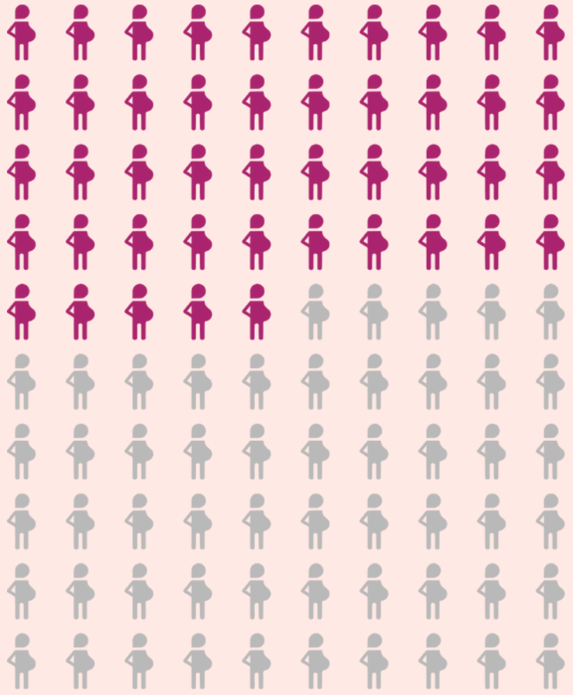

45 in 100

FUNDED BY NIHR | National Institute for Health and Care Research
UNIVERSITY OF LIVERPOOL
NHS Liverpool Women's NHS Foundation Trust
University of BRISTOL

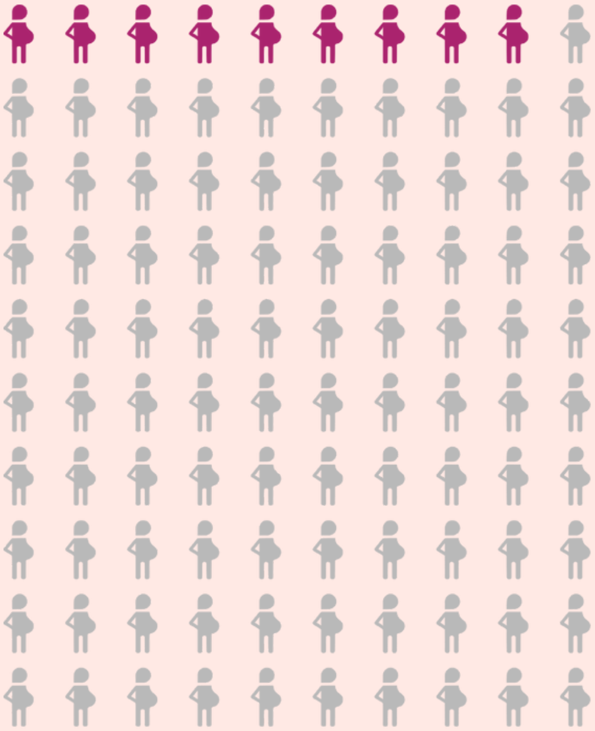

9 in 100

For subsequent births with low-risk pregnancies, there's a 9-13 in 100 rate of transfer into a consultant-led unit.

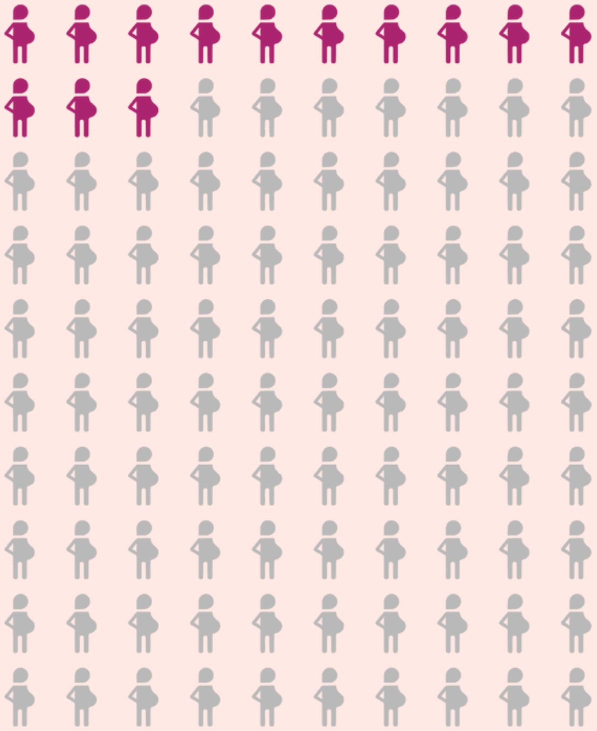

13 in 100

The time it takes to transfer from each birth setting will also vary.

Possible procedures or interventions during labour

Vaginal examinations offered during labour:

During your labour, a midwife or doctor may ask to perform a vaginal examination. A vaginal examination is done for many reasons, e.g. to see if your waters are intact or broken and how open your cervix is. This involves them putting their fingers into your vagina and up to your cervix. This will only ever be done with your consent. A cervical sweep may be offered to help start your labour and this would also involve a midwife putting their fingers into your vagina and moving their finger around the cervix in a 'sweeping' motion.

If an examination like this could trigger trauma for you, please let your carer know so they can help ensure their care helps you in a way that suits you. If you would like a chaperone, you are free to request one at any time. There are other methods to assess progress in labour and if you opt for a vaginal examination this can be stopped at any time.

FUNDED BY NIHR | National Institute for Health and Care Research
University of Liverpool
NHS Liverpool Women's NHS Foundation Trust
University of Bristol

A vaginal examination is usually offered every 4 hours in your first stage of labour to see how dilated your cervix is. Sometimes, this may be offered sooner if we have intervened, for example by breaking your waters, or of there is concern for you or your baby.

During the second stage of your labour, you may be offered more frequent vaginal examinations to see where your baby's head is, how far down it is, and the shape of the baby's head. After you have given birth to your baby, your placenta will come out. If your placenta doesn't come out by itself, you will be offered an examination.

The number of vaginal examinations will vary from person to person and from labour to labour. Not all labours progress the same and not everyone has the same circumstances and therefore the number you will be offered may be different. To perform any of these, your consent will be needed and you can always choose to say no. Your midwives and doctors will try not to do too many examinations as they are an infection risk.

How baby's wellbeing is checked during labour monitoring and procedures:

During your labour we will recommend that you have either intermittent or continuous monitoring of your baby. It is your choice whether or not to accept this. We will recommend continuous monitoring in some situations, and for this you will need to be in a consultant-led unit.

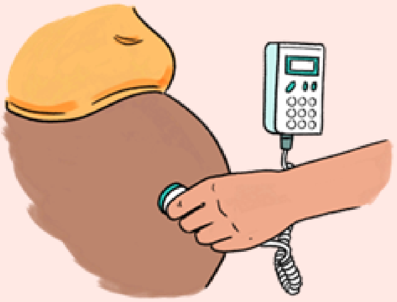

Intermittent monitoring is where your midwife will listen to your baby's heartbeat either using a special stethoscope or using a handheld device. This is available in all birth settings.

Continuous monitoring is where we monitor your baby constantly during your labour, this is called a CTG.

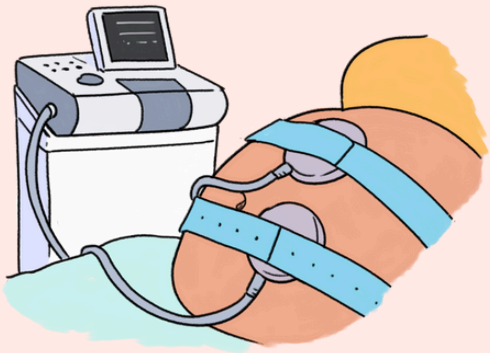

Cardiotocography (CTG) is a way of monitoring baby's heartbeat and your contractions at the same time. An elastic strap is placed over your abdomen and there are two round flat parts. One of these is looking at baby's heartbeat and the other is monitoring your contractions

The outcomes for both of these types of monitoring are similar. The rates of cerebral palsy in babies are also the same with intermittent monitoring and CTG. Continuous monitoring is also associated with more caesarean and instrumental births. However, seizures in babies are less common when CTG is used to monitor baby's heartbeat.

We can also continuously monitor your baby using a fetal scalp electrode. This is clip attached to baby's head during your birth. This is a way of monitoring baby if we are unable to pick up their heartbeat through your tummy reliably. In some hospitals, the fetal scalp electrode is used routinely as part of their fetal monitoring system called 'STAN'.

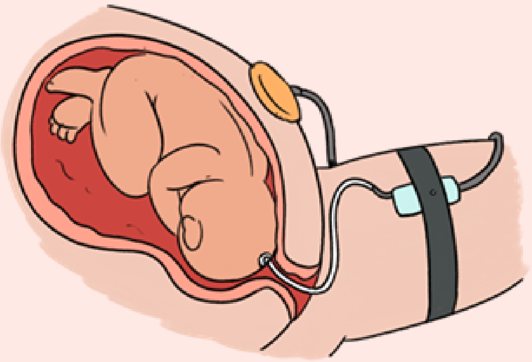

When the baby heart trace is normal, we can be sure that the baby is getting enough oxygen.

When it is not normal, it is not always the case that your baby is distressed.

To try to work this out we can examine you and tickle the baby's head. If your baby responds, we can be somewhat reassured that the baby is coping.
In some circumstances, a fetal blood sample can be taken to see if your baby is getting enough oxygen. This will require you to remain still whilst a doctor takes this sample from your baby's head. If this result is normal, it is reassuring, if it is not, we would recommend an urgent instrumental or caesarean birth.

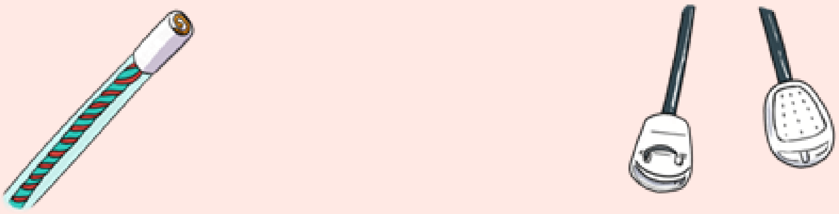

Different types of fetal scalp electrode

FUNDED BY NIHR | National Institute for Health and Care Research
UNIVERSITY OF LIVERPOOL
NHS Liverpool Women's NHS Foundation Trust
University of BRISTOL

Methods to reduce the risk of serious tears to the perineum:

There are different types of tears which can happen during childbirth, some heal by themselves and others need stitches. The more serious tears, the ones which include the anus, and can mean difficulties in holding in poo or wind, happen in around 6 in 100 of first births and around 2 in 100 subsequent births. Your midwife/doctor can try preventing these tears in multiple ways.

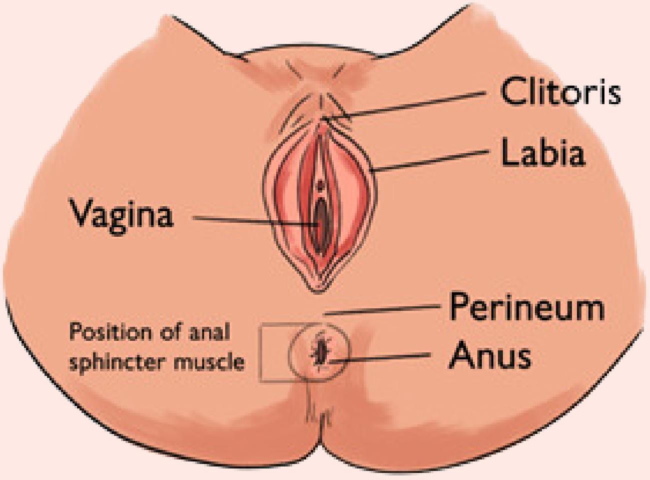

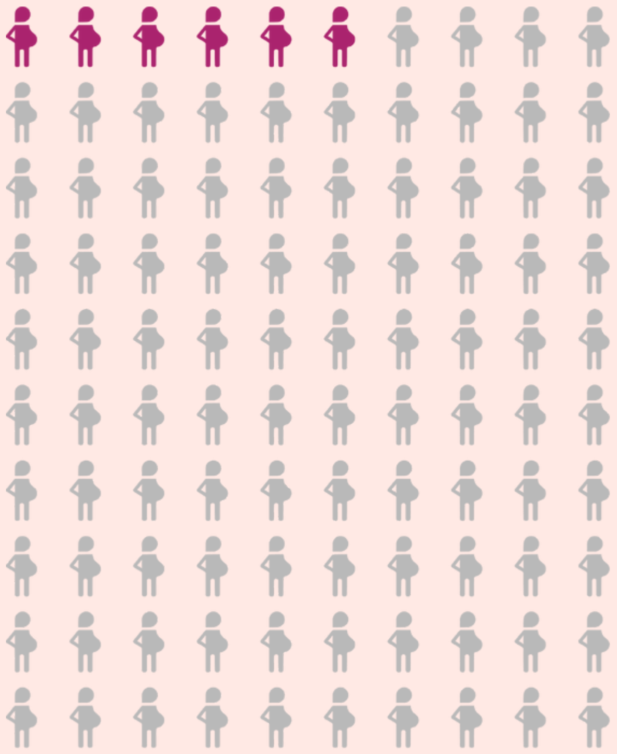

The most serious tears happen in 6 in 100 first births

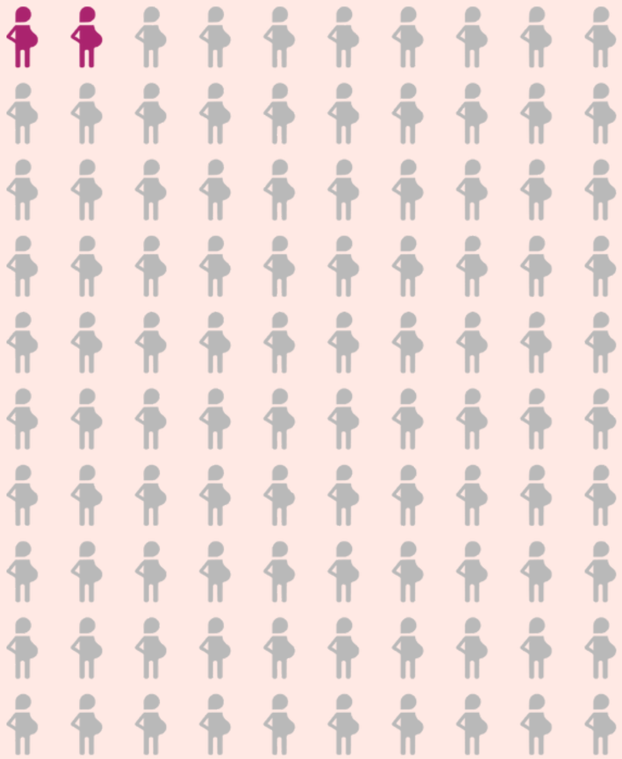

The most serious tears happen in 2 in 100 subsequent births

FUNDED BY NIHR | National Institute for Health and Care Research
University of Liverpool
NHS Liverpool Women's NHS Foundation Trust
University of Bristol

Guided Pushing:
This is where your midwife will tell you when to push/or not. You may be instructed to take a deep breath at the start of the contraction and then push through the contraction. You may also be advised to puff or pant when your baby's head is visible. These are both methods to help reduce the risk of a more serious tear. This guided pushing can also reduce the likelihood of needing an assisted birth which increases your risk of a more serious tear to the vagina.

Massage/Warm Compress:
Holding a warm compress on your perineum (the area between your vagina and your back passage) or massage your perineum with water soluble lubricant during the time you are pushing.

Guiding your baby's head out whilst supporting the perineum at the time of the baby's head being born can help reduce the risk of a more serious tear.

Episiotomy:
You may be offered this if your baby needs extra room to be born, and your midwife or doctor feel that a more serious tear is likely and are trying to reduce this risk. This is more likely to be considered during an instrumental vaginal birth or if your midwife/doctor suspects that your baby is not getting enough oxygen and needs to be born quickly.

When Episiotomy may be offered:

An episiotomy is a small cut made at the opening of the vagina at the time of birth.

During labour, an episiotomy may be recommended to you. An episiotomy will only be offered to you if your midwife or doctor feels that there is a risk of a more serious perineal tear, or the birth of your baby is very urgent . This may be necessary during an instrumental vaginal birth

An episiotomy is a cut which is made at the opening of the vagina into the perineum to give you baby more room to be born. The aim of an episiotomy is to reduce the seriousness of a perineal tear or to birth you baby as quickly as possible

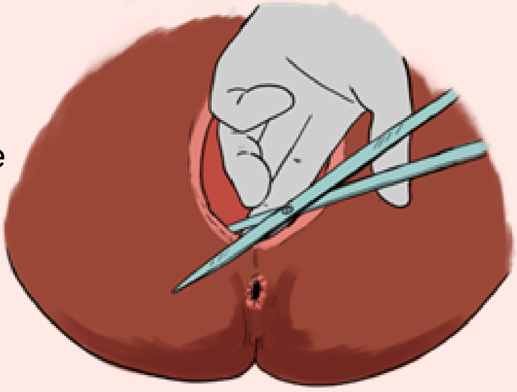

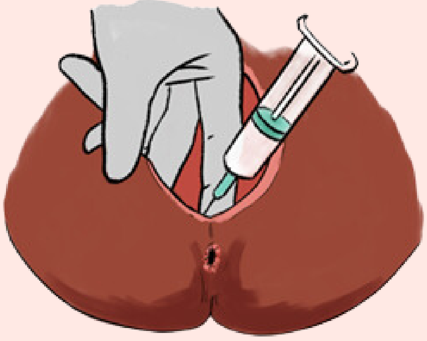

If you accept the episiotomy, you will usually be numbed using local anaesthetic and you will require some stitches afterwards. This may happen in your labour room or in a surgical theatre, this would be discussed with you and the reasons for the choice of location.

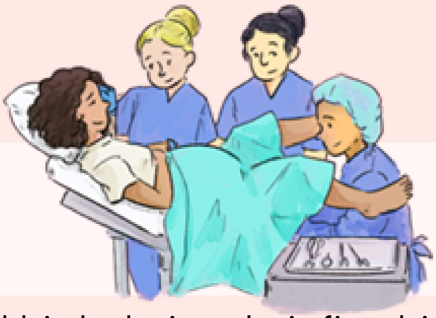

When an Instrumental or caesarean birth may be offered or recommended and why:

Around **18 in 100** women experience instrumental vaginal birth during their first birth, and **5 in 100** during subsequent births

Reasons include delay during birth, maternal request, or concerns about the baby.

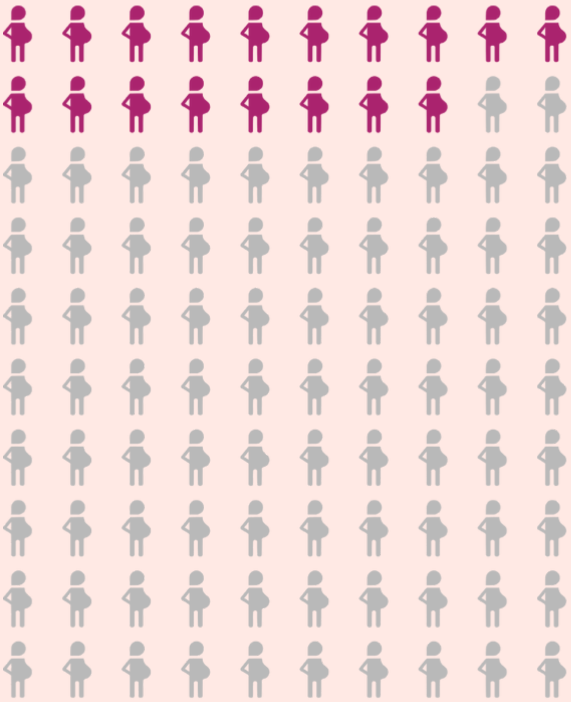

18 in 100 women have an instrumental vaginal birth for their first birth

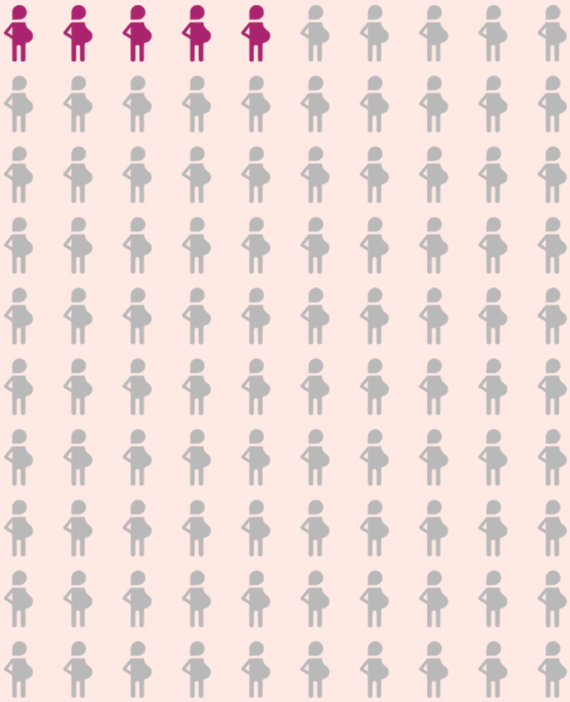

5 in 100 women have an instrumental vaginal birth in a subsequent birth

FUNDED BY NIHR | National Institute for Health and Care Research
UNIVERSITY OF LIVERPOOL
NHS Liverpool Women's NHS Foundation Trust
University of BRISTOL

There are two main types of instruments which can be used. One is called forceps and these are like big metal spoons.

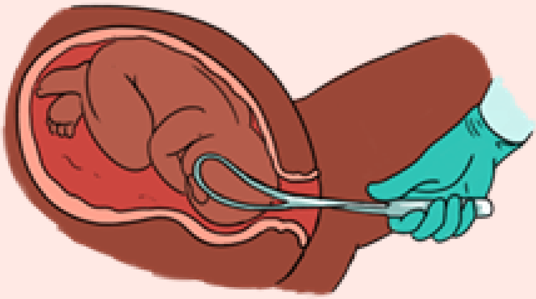

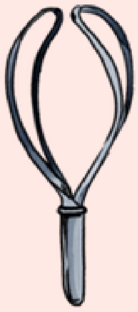

The other type of is a suction cup (ventouse), these instruments are put on your baby's head to help guide them out whilst you are pushing.

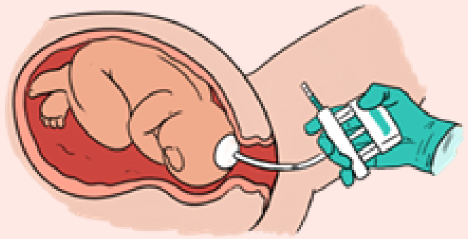

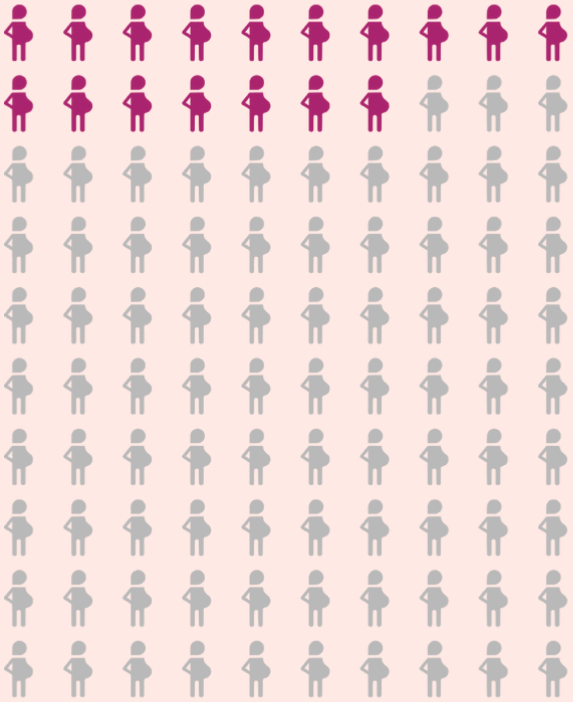

17 in 100

Sometimes you will be offered an episiotomy to reduce risk of tears to your perineum because it is more likely you will have a more serious perineal tear when having an instrumental birth. This happens in around 17 in 100 instrumental births.

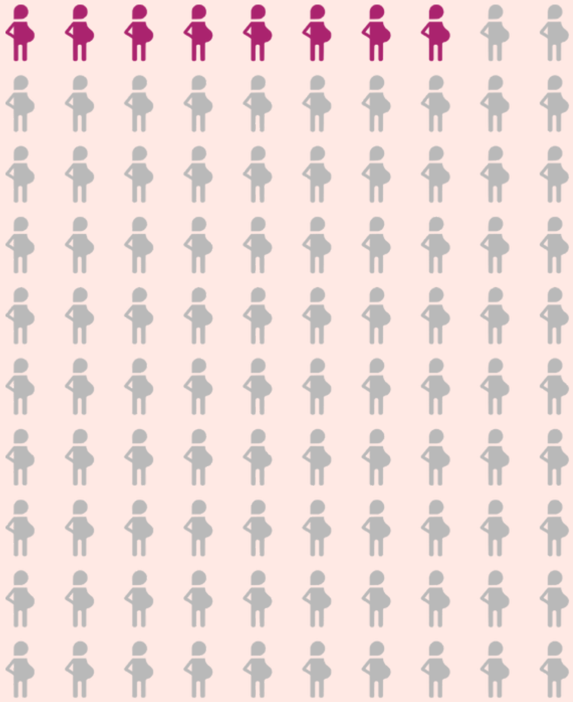

8 in 100

Around **8-12 women in every 100** who have a forceps delivery have a more serious perineal tear.

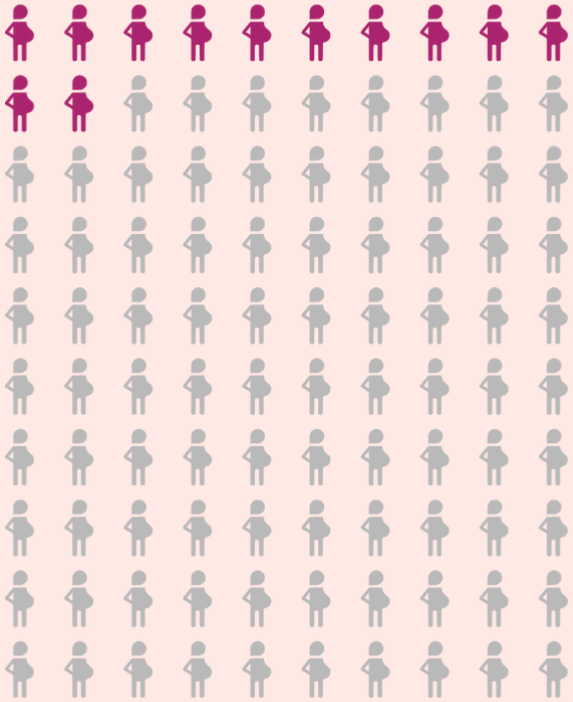

12 in 100

FUNDED BY NIHR National Institute for Health and Care Research
University of Liverpool
NHS Liverpool Women's NHS Foundation Trust
University of Bristol

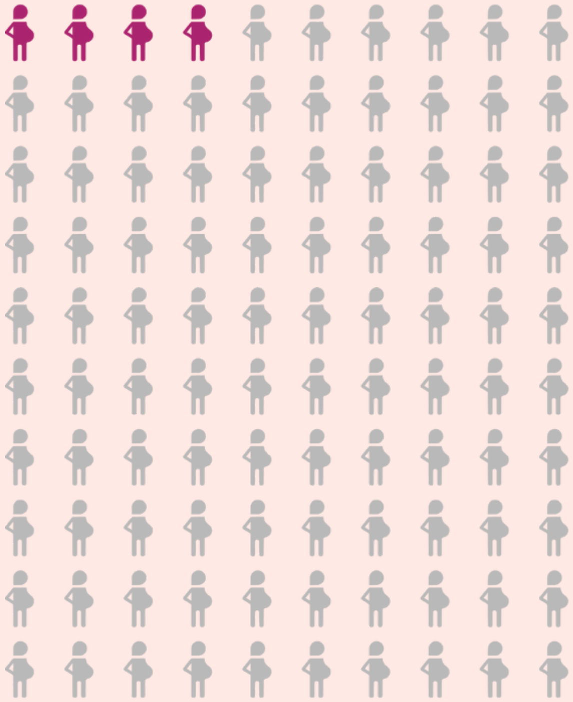

With a ventouse, sometimes the cup pops off and makes a sound and will need to be put back on, or there will need to be a switch to forceps.

Up to **4 in 100** women who deliver with a ventouse will have more serious perineal tear (3rd or 4th degree tear).

4 in 100

If it is not viable to try an instrumental birth or if it doesn't work, a caesarean birth can be offered. This is where your baby is delivered through a cut to your abdomen and uterus. This is done in a surgical theatre with multiple doctors and your midwife present. You get an injection into your back to numb you from your chest to your toes so that you don't feel pain when your abdomen is cut into.

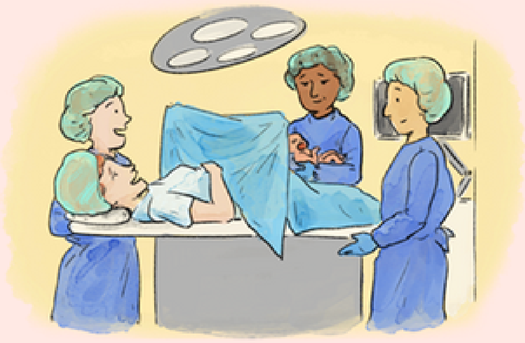

When you are in the second stage it may well be faster and safer for your baby to be born by instrumental vaginal birth than an urgent caesarean birth. For example, if the baby's head is too low, a caesarean birth is associated with difficulties in your baby's head being born (known as impacted fetal head) and this can be associated with babies getting a lack of oxygen or having injuries during birth.

FUNDED BY NIHR | National Institute for Health and Care Research
UNIVERSITY OF LIVERPOOL
NHS Liverpool Women's NHS Foundation Trust
University of BRISTOL

Pain relief

Use of medical pain relief in labour including gas & air, oral medications (i.e. paracetamol, dihydrocodeine), injectable medications (i.e. pethidine, diamorphine) & epidural

Women use a variety of things to cope with pain during labour. There are medical strategies and non-medical strategies.

Non-medical pain relief:
Some women find that having a bath or shower may reduce pain in early labour. Some also find that breathing exercises and massage also reduce pain in early labour. TENS machines, whist they do not have lots of evidence of their effectiveness, have no evidence of harm and any choice of pain relief will be supported.

Paracetamol and Dihydrocodeine are available as tablets. The effect of these tablets lasts for hours. Paracetamol can take around 30 minutes to work and dihydrocodeine can take around 90 minutes.

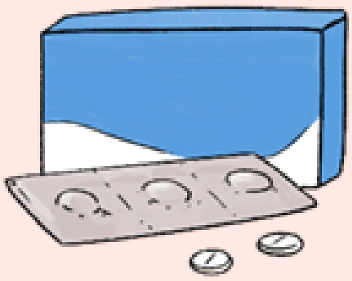

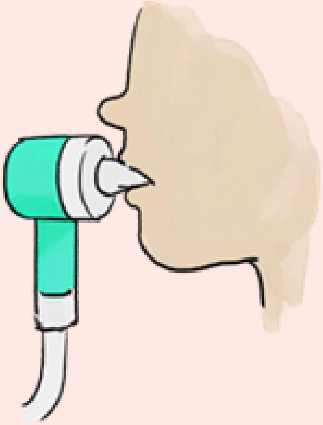

Gas and Air (Entonox) is available in all settings. This is a very effective form of pain relief and the effects wear off quickly if you stop using it. Gas and air can be used in a birthing pool but it can make you feel light-headed and nauseous.

Injected medications (opioid medications e.g. diamorphine, pethidine), they take around 20-30 minutes to work

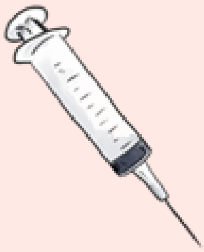

FUNDED BY NIHR | National Institute for Health and Care Research
UNIVERSITY OF LIVERPOOL
NHS Liverpool Women's NHS Foundation Trust
University of BRISTOL

They may not always take the pain away completely but may make the pain more manageable for you. They last for 2-4 hours. They may also help you feel more relaxed. However, these medications have side effects such as drowsiness, feeling sick and being sick, short term breathing problems and drowsiness for your baby. The drowsiness in your baby can last several days so it can be more difficult to breast feed and, if you feel drowsy it may impact you if you wish to give birth in water. However, giving birth in water is still an option as long as it has been around 4 hours since you received the medication and there are no concerns about safety. This is because it is advised that you don't enter a birthing pool or bath whilst you feel drowsy or within 2 hours of receiving an opioid medication.

There are two kinds of **patient-controlled pain relief** where you decide and oversee when you receive doses of the medication.

Through your back: An epidural is an injection that goes into your back. This numbs you from the bottom of your chest to your toes. It is more effective than opioids at relieving pain. The epidural may need to be adjusted or replaced, and you will need to have a cannula in your hand or arm. An epidural does not increase the likelihood of having a caesarean birth. The epidural doesn't usually cause long term back pain either. It does however make the second stage of labour longer and increase the chance of an instrumental birth. You may also experience a severe headache after you give birth. You will require more monitoring which will be slightly different depending on which hospital you have chosen. But you can assume any position apart from lying flat on your back and, if you feel like you have sufficient leg strength and sensation, you can mobilise as you please, with assistance if you need it. An epidural in a twin or triplet pregnancy improves the likelihood and timing of an instrumental vaginal birth, and, if needed, a quicker birth by an urgent caesarean birth.

Through a vein (Remifentanil): having this medication means you are less likely to need an epidural or instrumental birth compared to if you receive the injected medication called pethidine. You are also more likely to have a spontaneous vaginal birth, but you may need extra oxygen than if you had pethidine. You will have to have a midwife present the entire time and continuous monitoring if you have any other risk factors. This increases your baby's risk of respiratory distress. However, this is an effective way of relieving pain.

Additional Supportive Medication:

You can be given an anti-sickness medication to help with feeling sick or being sick during your labour They can be given at the same time as the pain medication.

FUNDED BY NIHR | National Institute for Health and Care Research
University of Liverpool
NHS Liverpool Women's NHS Foundation Trust
University of Bristol

Environment during labour

Labour companions who you can choose to have present during labour and their role in the process.

Having a birth partner/labour companion is beneficial as they are there to support you specifically. This can mean you have a better birth experience. How many birth partners can vary from trust to trust and also between the different areas of the hospital. You can choose any adult you wish to be your birthing partner.

Keeping mobile and adopting different positions during labour

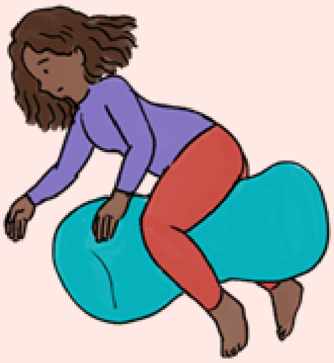

During your labour and birth, you may wish to get into positions that make you most comfortable and feel right for you. Some positions are good for example, upright positions or lying on your side after you are fully dilated reduces your chance of needing an instrumental birth. Lying on your side may also increase the chance of having a vaginal birth without any extra help. We do not recommend lying flat on your back for long periods, although this may be necessary at times.

If you choose to have CTG as a way of monitoring your baby, this may restrict how mobile you can be. If you would like to have your baby monitored intermittently, you may be asked to take on a different position to allow your midwife or doctor to do so

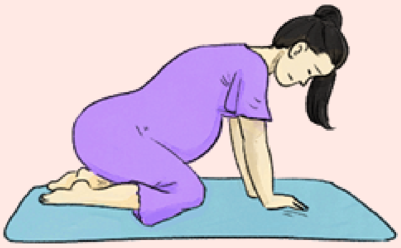

Some things will have an impact on the positions we recommend or that you can physically get into. For example, an epidural may reduce your mobility.

Ultimately, the position you choose is down to you and you are encouraged to choose any position that you are comfortable and happy being in.

Possible labour complications

Complications relating to the mother during labour:

Labour carries the chance of a list of complications which can affect the mother. These can be split into moderate to severe complications which are common and complications relating to bleeding.

Labour is often straightforward, however, sometimes there are complications which means action is recommended. To try to identify issues before they become problems, we will monitor your vital signs (e.g. blood pressure, heart rate, how much you wee and drink) and your baby.

High maternal temperature: This is common and happens in around 10 in 100 women
This requires investigations such as blood tests and swabs and antibiotics and fluids may be needed. We may suggest additional tests and may recommend urgent birth to protect both mother and baby.

Raised blood pressure: Whilst this can happen any time in pregnancy, sometimes it happens in labour. We may want to take bloods and a urine sample and give some medications to reduce blood pressure.

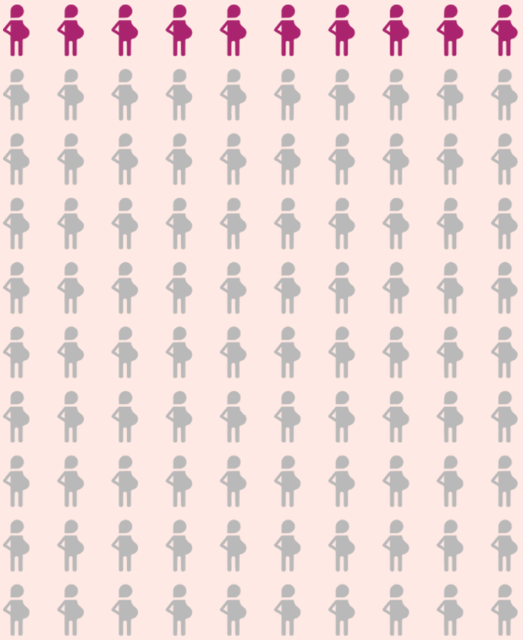

High maternal temperature happens in 10 in 100 women

Uterine hyperstimulation is where the uterus (womb) gets too many contractions in a short space of time, this can be uncomfortable but can also cause the fetus to not get enough oxygen.

This can sometimes happen if we have tried to help her labour progress using a medication called oxytocin (a medication that helps your womb contract). If this is the case, we can stop the medication and if necessary, give a drug to relax the uterus.

Bleeding before or during labour (Antepartum Haemorrhage): This is not normal and if this happens, we will recommend continuous fetal monitoring. Most serious is if your placenta is separating from the uterus before your baby is born, if this is the case, we will recommend an urgent caesarean birth or an urgent instrumental birth.

FUNDED BY NIHR National Institute for Health and Care Research
University of Liverpool
NHS Liverpool Women's NHS Foundation Trust
University of Bristol

Complications relating to the baby:

There can be some events in labour that are complications for your baby these are:

Baby doing a poo: This is your baby's first poo (meconium) and normally it happens after your baby is born but sometimes it happens before they are born. This can be normal (e.g. baby is post-dates (41+ weeks), or it can be a sign of distress during labour. We are concerned about this not only because it is a sign of possible distress, but also because if a baby breathes in this meconium, they can become unwell. For this reason, your midwife or doctor may want to transfer you to a consultant-led unit if you are not already at one. This enables continuous monitoring and the availability of doctors that specialise in looking after newborn babies if required.

Fetal distress: This is when we suspect that the baby may not be getting enough oxygen. This can be for unknown reasons but also because your uterus is tightening too frequently without getting enough rest between contractions. This can mean your baby does not get enough oxygen. This can also be because your baby has an infection.

Cord Prolapse: This is where the umbilical cord comes out of the womb before baby is born. This complication is severe but uncommon. It happens in 0.1-0.6 in 100 births. If this happens you will most likely be moved immediately to a surgical theatre for a caesarean birth unless an instrumental vaginal birth would be quicker or transferred urgently into a consultant-led unit if you are not there already.

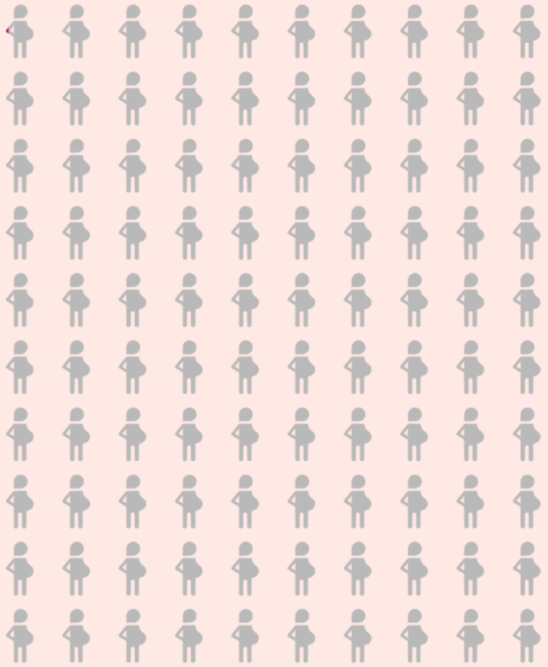

0.1 in 100

Cord prolapse happens in **0.1-0.6 in 100** births

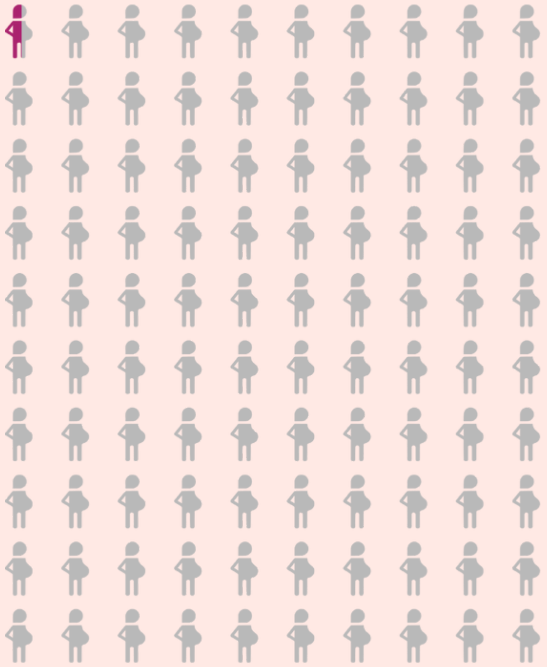

0.6 in 100

FUNDED BY NIHR | National Institute for Health and Care Research
UNIVERSITY OF LIVERPOOL
NHS Liverpool Women's NHS Foundation Trust
University of BRISTOL

Shoulder dystocia: This is where baby’s shoulder gets stuck after their head has come out. This happens in 0.58–0.70 in 100 vaginal births. This can result in a brain injury to baby as they cannot get enough oxygen or an injury (brachial plexus injury) which impacts their shoulder and arm. Shoulder dystocia causes a brachial plexus injury in 0.043 per 100 live births in the UK and 10 in 100 of those that end up with this injury will have permanent neurological problems.

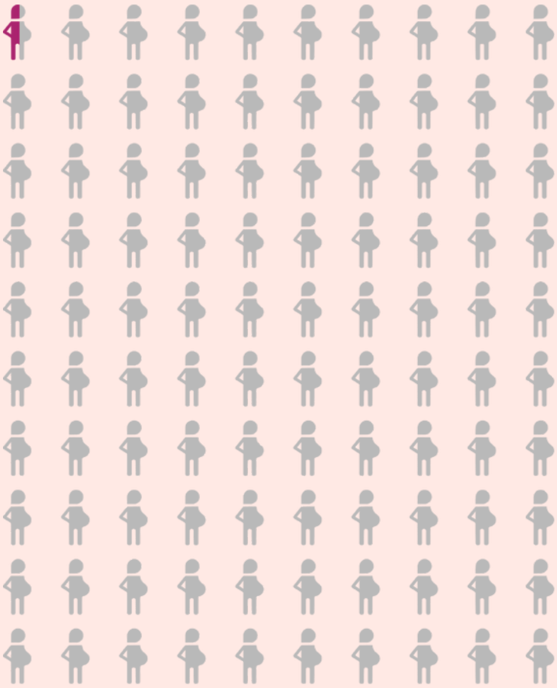

0.58 in 100

Shoulder dystocia happens in fewer than 1 in 100 (0.58–0.70 in 100) vaginal births.

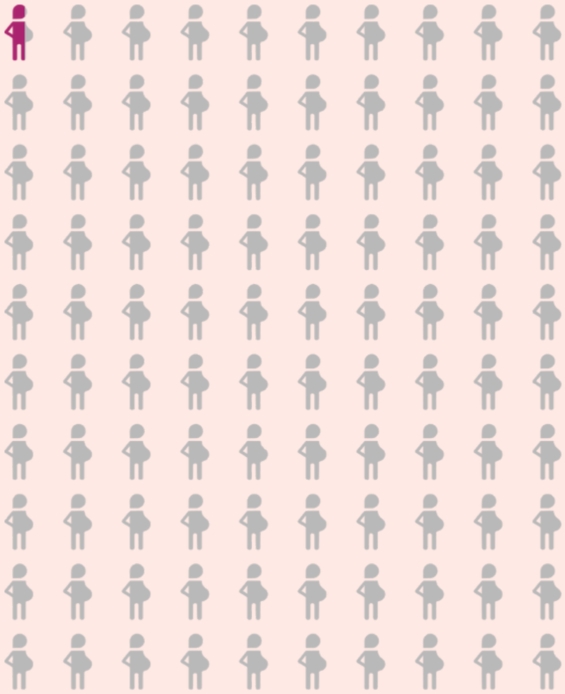

0.70 in 100

Chorioamnionitis: is an infection which affects your uterus and the baby. This will require antibiotics.

Neonatal infection: If your waters have broken before labour starts your baby has 1 in 100 risk of neonatal infection compared to 0.5 in 100 if your waters were still intact.

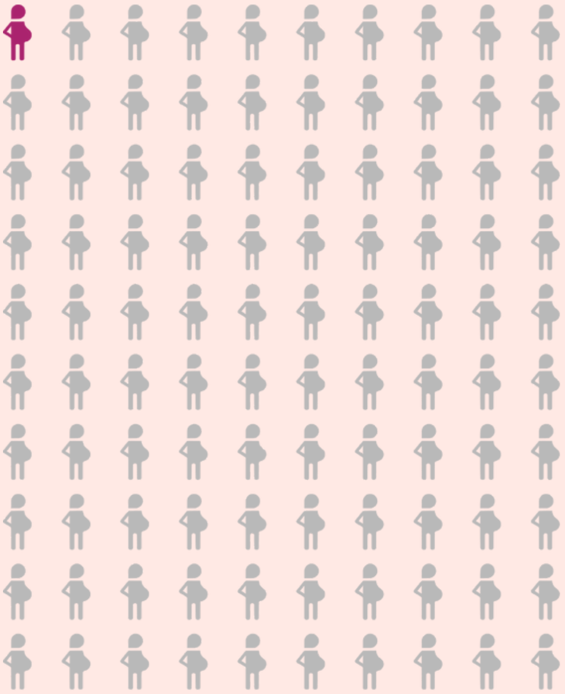

If your waters break before labour starts, the risk of your baby developing an infection is 1 in 100

FUNDED BY NIHR | National Institute for Health and Care Research
University of Liverpool
NHS Liverpool Women’s NHS Foundation Trust
University of Bristol

Outcomes for the baby

Condition of baby when they are born

If your baby comes out and needs some help breathing, or their heart rate is not at a normal level, they may need some additional support. If this happens, the team of midwifes (and paediatric doctors if you are in a hospital) will step in to help immediately. There may be lots of people coming into the room to help. This will mean you will not get to have skin-to-skin straight away as your baby needs to be taken to the right place to be resuscitated. For most babies, all that will be required is some encouragement to breathe.

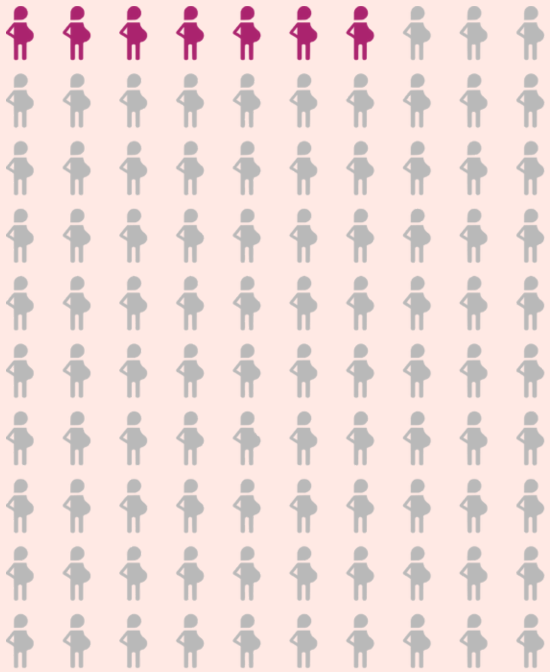

However, for others more support is required, and in some cases, we need to help babies' hearts to beat. Around 7 in 100 babies will need some extra help to breathe after birth.

If this happens, we understand that this can be worrying but your baby will only be away from you as long as it takes to stabilise them. Usually, they will be back with you very quickly, but sometimes it is necessary for them to be transferred to the neonatal unit for further care and more tests.

If we suspect that your baby may be born needing support, we may ask to take a blood test from the umbilical cord after you have given birth to the baby and the placenta.

When your baby is born, your midwife will do immediate observations on your baby (called an APGAR) to decide if it is necessary to resuscitate them or call for help. It may be necessary to do other tests for example check oxygen levels
.

All babies will have an early examination to make sure there are no obvious problems, and to check things such as their head size, weight and temperature checked.

FUNDED BY NIHR | National Institute for Health and Care Research
University of Liverpool
NHS Liverpool Women's NHS Foundation Trust
University of Bristol

Experiences after birth

Possible experiences or symptoms immediately after birth

After you give birth, it is expected that you will experience some things which may be new for you. You may experience pain, some different emotions, or other physical symptoms.

Most women will experience an element of pain or discomfort after giving birth. Even without any injuries, your uterus (womb) needs to contract back to its original shape and size. This is often experienced as a cramping sensation, often described as being similar to period pains. You may also experience pain due to bruising, or injury to your perineum.

Bleeding is normal following birth, and it is called lochia. Normally, the bleeding stops by the time you are 12 weeks postpartum and may be quite heavy at first. However, some women will experience significant bleeding after birth (Post partum haemorrhage), this is usually within the first 24 hours but can be in the weeks following birth. If you are frequently soaking through pads or passing clots it is important to seek help for some advice.

You may feel lots of emotions such as anxiety and may feel exhausted after giving birth. You may also feel anxious or down during the first week. This is normal and it shouldn’t last more than 2 weeks. If it does last longer, this could be a sign of postnatal depression. If you are concerned, it is important that you seek help from your midwife or GP.

FUNDED BY NIHR | National Institute for Health and Care Research
UNIVERSITY OF LIVERPOOL
NHS Liverpool Women's NHS Foundation Trust
University of BRISTOL

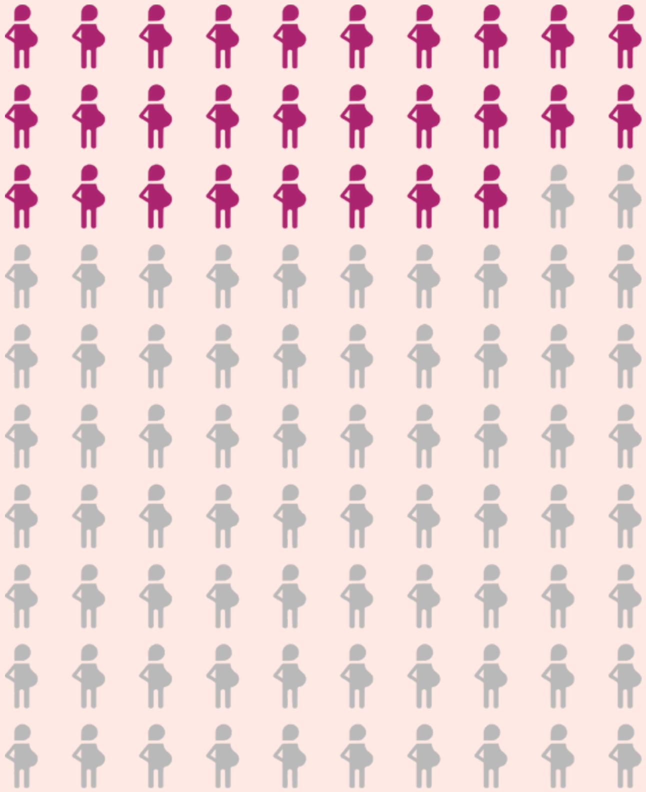

Around **28 in 100** people will experience urinary incontinence in the first 2 weeks following a spontaneous vaginal birth.

Depending upon how your labour has been, you may experience other physical symptoms. One of these symptoms is urinary incontinence. This is where you accidently urinate. This can be because of the birth or a tear. Some women will experience the opposite and find they struggle to voluntarily urinate postpartum. This is more likely if you have had an assisted birth, prolonged labour, or an epidural. Around 28 in 100 people will experience urinary incontinence in the first 2 weeks following a spontaneous vaginal birth.

Feeding the baby following birth

There are three options for feeding your baby. These are breastfeeding, formula and a mix of both (combi feeding).

You will be encouraged to start to feed within an hour of birth where possible. Your midwives and doctors will support whichever decision you make regarding the type of milk you choose for your baby.

We recommend breastfeeding where possible as breast feeding has additional health benefits.

If you choose to breastfeed and you would like help, you can get support on how to breastfeed and there are even medications available which can help. There are lots of factors which can impact how viable breastfeeding is for you and your baby. It is not right for everyone, but if you choose to, those benefits are there no matter how short you breast feed for.

If you would like to use formula milk, you can use the first infant formula for the whole first year of your baby's life. To prepare feeds safely you can sterilise their bottles.

Birth Options

Pelvic floor injuries that can happen during labour, examination of the area to assess these, and potential issues with this area following birth:

Pelvic floor injuries can be quite common following labour. They can be grazes right through to more serious perineal tears involving your anus and rectum.

When there is an injury just to the skin, stitches are not generally needed (1st degree tears).

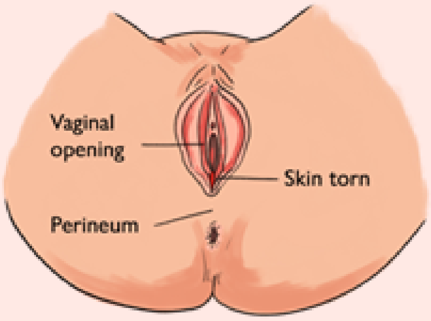

First degree tear

When there is injury to the perineal muscles, but not the muscles around your anus, stitches will be needed. (2nd degree tears)

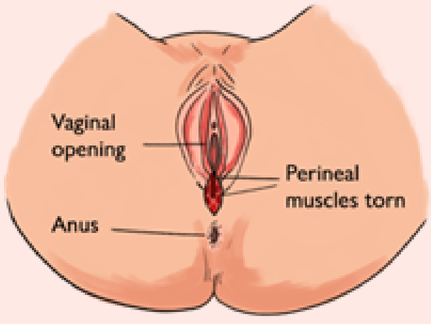

Second degree tear

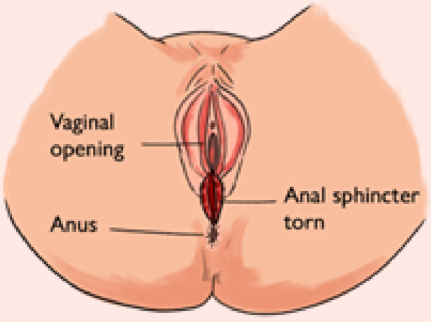

Third degree tear

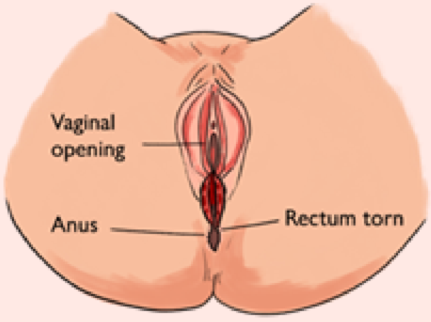

Fourth degree tear

The most serious tears are when there is an injury that includes your anus, or your rectum. These will need stitches and antibiotics. (3rd and 4th degree tears)

After a pelvic floor injury, you may experience some symptoms in that area. These may include, pain, a change in sensation, infection and your wound breaking down. If you have an infection, you may require antibiotics.

FUNDED BY NIHR | National Institute for Health and Care Research
University of Liverpool
NHS Liverpool Women's NHS Foundation Trust
University of Bristol

Some women will also experience bladder and bowel issues. This may involve pain or accidently urinating or opening your bowels when you didn't intend to. This may be short lived for some women and they may only temporarily experience this,. In women who have sustained the most serious tears (third and fourth degree tears), 60-80 in 100 women have no symptoms 12 months postpartum.

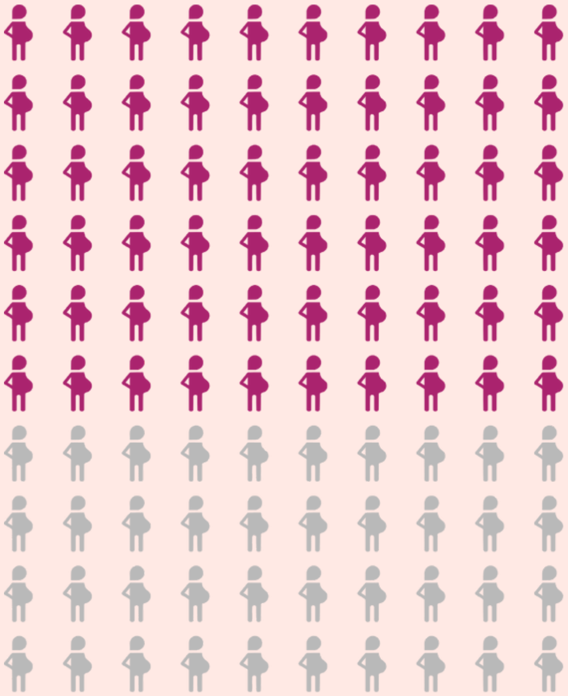

60 out of 100

60-80 out of 100
women with the most serious tears have no symptoms a year later

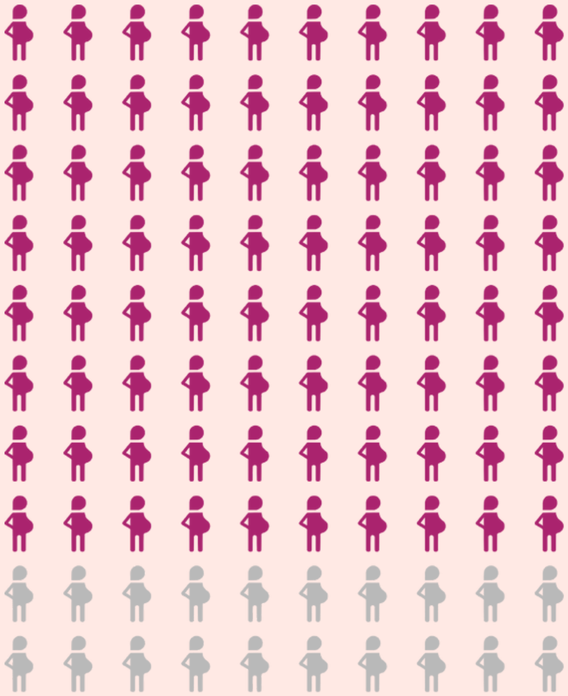

80 out of 100

Around 49 per 100 women who experience urinary incontinence after a vaginal birth will still be experiencing this a year later. If required you will be managed by a specialist team of doctors and supported by them, specialist midwives and physiotherapists.

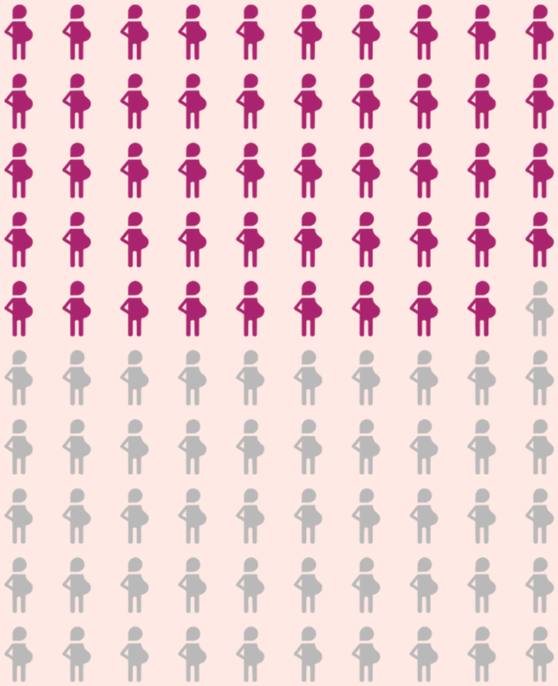

49 out of 100

FUNDED BY NIHR | National Institute for Health and Care Research
UNIVERSITY OF LIVERPOOL
NHS Liverpool Women's NHS Foundation Trust
University of BRISTOL

Some women may also have problems with their mental health following a perineal tear. This can be for lots of reasons, even if they don't experience things such as urinary or faecal incontinence. A tear like this could interfere with a woman's body image, self-esteem, intimate relationships, and bodily functions. Therefore, feeling emotions related to this can be anticipated and if you would like to receive help for this, please do contact your GP, health visitor or midwife.

Possible mental health experiences following birth (may be in the short and long term):

Some women experience mental health issues after having a baby, it is very common to have some experience of deteriorating mental health in the weeks following the birth of your baby. Some women find these symptoms pass quickly, others need more in depth support with either talking therapies, medication or admission to hospital.

Baby blues is where your mood is low and this is common in few days after giving birth. It happens in 3-8 in every 10 women. This is normally mild and goes away by itself within 2 weeks or less. During this time, you may be struggling to sleep, cry more than usual, feel anxious, be irritable, struggle to concentrate, or find your mood is changing a lot. This experience will be different from person to person. If this lasts more than 2 weeks, it could be a sign of postnatal depression. With any major life changing event there is going to be an impact on you but it is important to look out for any signs that could indicate something isn't right.

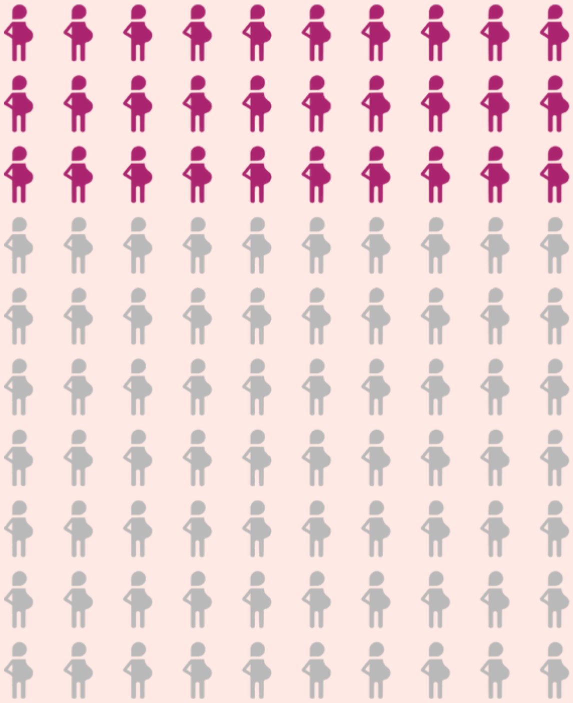

30 out of 100

Baby blues happen in **30 – 80 out of 100** women

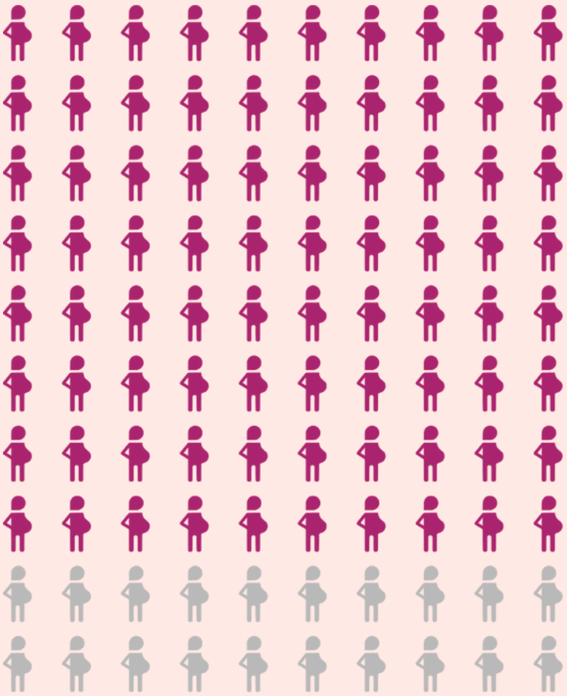

80 out of 100

FUNDED BY NIHR National Institute for Health and Care Research
University of Liverpool
NHS Liverpool Women's NHS Foundation Trust
University of Bristol

Postnatal depression is a spectrum of mental health problems and everyone will experience slightly different symptoms. Around 15-20 in 100 women will experience depression and anxiety in the first year after childbirth. If you have had struggles with mental health previously you may be more likely to experience problems postnatally. You may find you no longer have interest in the things you once did, you may have low mood and exhibit physical and behavioural symptoms also. This can start from the day you give birth all the way up until 1 year postpartum, it often develops gradually.

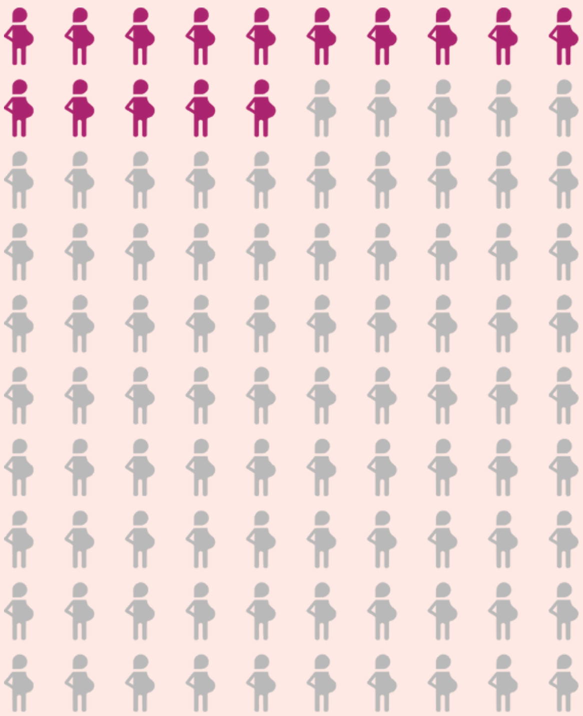

15 out of 100

Postnatal depression happens in 15-20 in 100 women

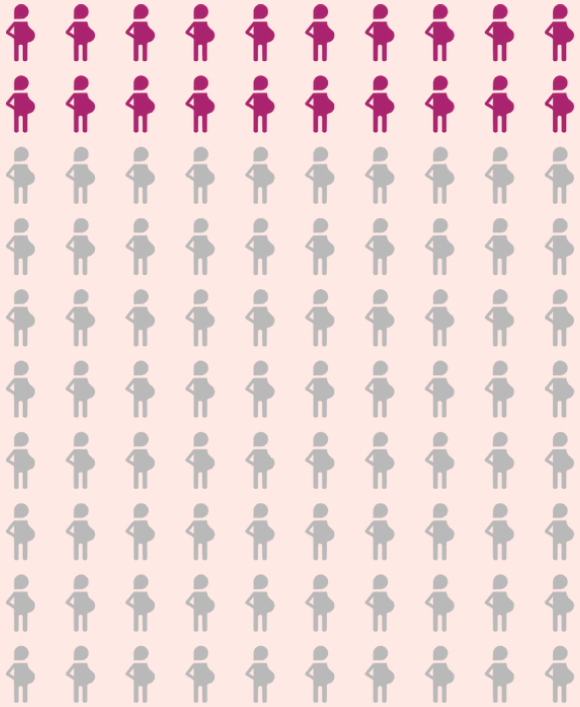

20 out of 100

Postnatal anxiety (15-20 out of 100 women), may include worrying thoughts that keep happening, feelings of panic or panic attacks, constant restlessness, irritability and feeling tense.

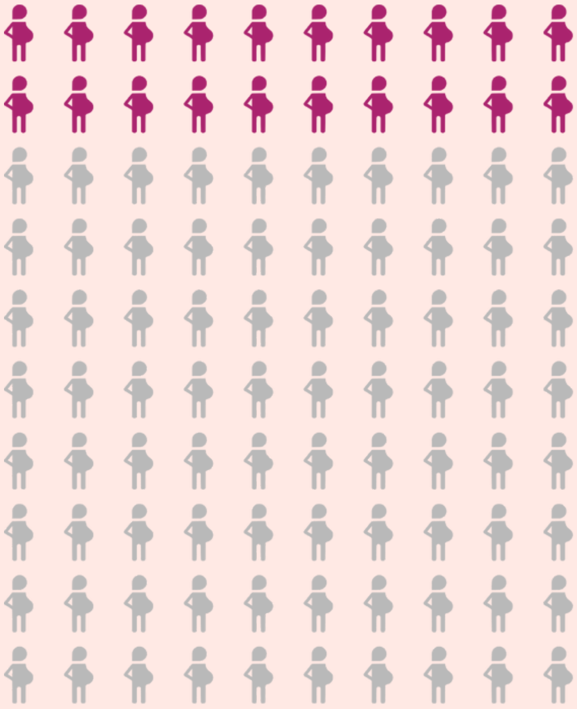

15 out of 100

Postnatal anxiety happens in 15-20 in 100 women

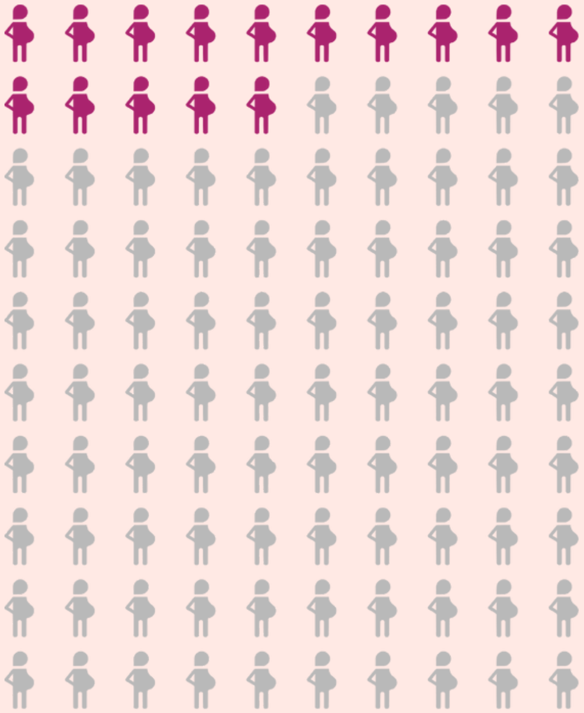

20 out of 100

FUNDED BY NIHR | National Institute for Health and Care Research
UNIVERSITY OF LIVERPOOL
NHS Liverpool Women's NHS Foundation Trust
University of BRISTOL

Some women will experience **post-traumatic stress disorder (PTSD)** following birth. This is a condition that happens when someone has been exposed to a traumatic event and the negative feelings that you would expect to have after such an event don't go away. This can start to affect and interfere with your daily life. An instrumental vaginal birth is a risk factor for PTSD. This could be for many reasons but may be due to feeling a loss of control. Some women will have fears about a future pregnancy/birth.

Post-partum psychosis affects 0.1 in 100 women after giving birth and it usually starts within 2 weeks of giving birth. Women who already have bipolar disorder are at a greater risk of this condition. These symptoms can include being excited or elated, being unusually chatty, feeling like your thoughts are racing, feeling really fearful or paranoid, not behaving like you normally would, or having heightened senses. If you, or someone you know exhibits signs like this, it is important to seek out help.

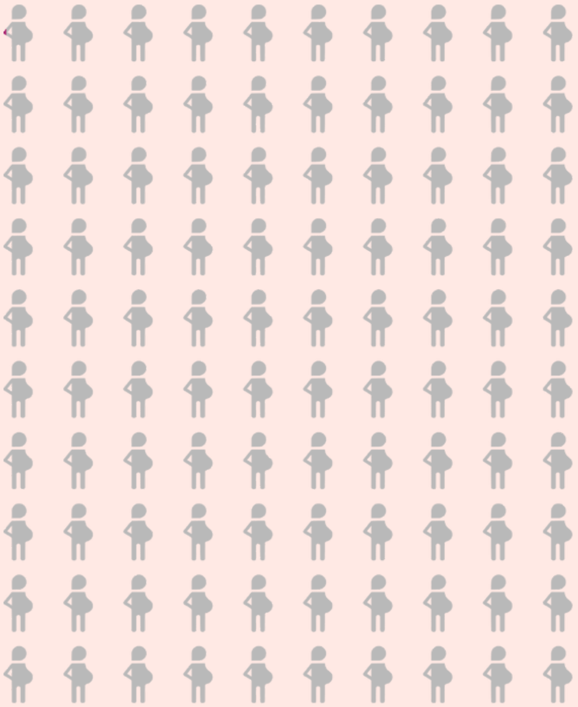

Postpartum psychosis happens in 0.1 in 100 women

Supplementary information for if you decide to have a vaginal birth

The signs and symptoms of labour

There are many signs and symptoms which may indicate you are in labour. These may include lower back pain, a mucus plug coming out, your waters breaking and feeling pressure down below.

During your pregnancy you may have experienced Braxton-Hicks. Your contractions may feel similar but will continue for a prolonged period of time. The contractions you have may feel like extreme period pains. If you touch your abdomen, it may even feel hard when you feel a contraction, then soften when the contraction eases.

FUNDED BY NIHR | National Institute for Health and Care Research
University of Liverpool
NHS Liverpool Women's NHS Foundation Trust
University of Bristol

You may also experience some lower back pain. It may even feel heavy or achy. As well as this, you may experience a feeling of pressure down below.

A mucus plug may also come out of your vagina. This mucus plug is a sticky jelly like pink substance. For some people, it may come out all at once and then for others it will come out in several pieces. This indicates that your cervix is starting to open up. Labour can come very soon after or take a few days and not everyone will experience a mucus plug coming out.

Your waters may also break before or after your labour starts. During pregnancy, your baby grows in a bag of fluid called the amniotic sac. When your waters break, it is this bag of fluid breaking and exiting out of the vagina. For some it will be a slow trickle and for others it will come all in a big gush. This is a clear fluid which may be a bit blood stained to start off with.

Waters breaking before labour

If your waters break before you go into labour, it is best to contact your midwife or maternity unit.

They will be able to ask you some additional questions such as if you are having any bleeding and if you are feeling unwell, this will help to decide the next steps in your care.

You may go into labour naturally within 24 hours. If not, and you are full term, you may be offered an induction of labour. 60 in 100 labours start naturally within 24 hours of the waters breaking.

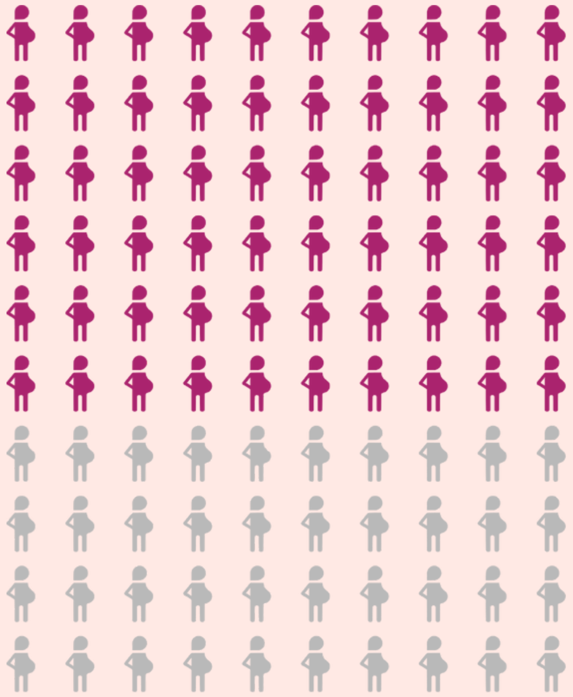

60 in 100 labours will start naturally within 24 hours of the waters breaking

FUNDED BY NIHR | National Institute for Health and Care Research
University of Liverpool
NHS Liverpool Women's NHS Foundation Trust
University of Bristol

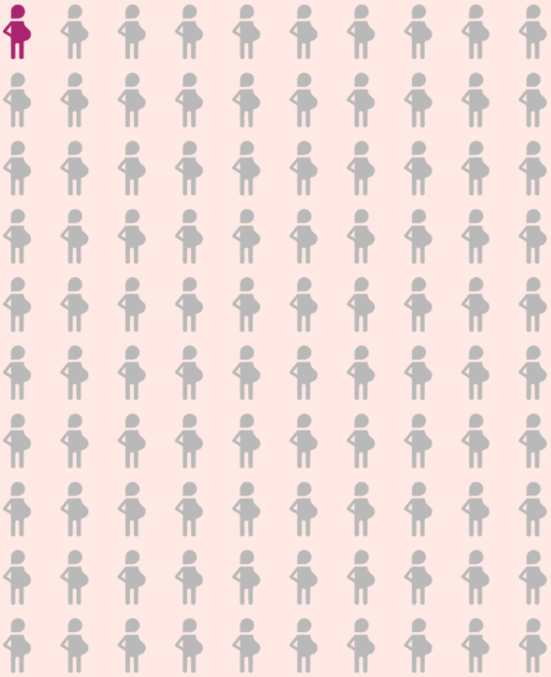

If your waters break before labour starts, the risk of your baby developing an infection is 1 in 100

If labour does not start within 24 hours, this is where you are given medications to try and start your labour. You can continue to wait for your labour to start if you would prefer. Waiting for your labour for a prolonged time can be a risk to you and your baby. The risk of serious neonatal infection is 1 in 100 for women whose waters have broken before labour starts compared to 0.5 in 100 for those who have their waters intact.

This risk increases as time goes on. If at any point your baby's movements decrease it is important to report this to your local maternity service as soon as possible. You may also be offered some antibiotics depending on how long your waters have been broken. You can continue bathing and showering as normal as they don't increase your risk of infection, however, sexual intercourse might.

Medical professionals who may be present at your labour

During your labour there may be midwives, doctors, midwifery care assistants, student midwives and student doctors present. Who is present will depend on where you are having your baby. How many will be present depends on your birth and how it is going. If you don't want students present that is entirely your choice.

FUNDED BY NIHR National Institute for Health and Care Research
University of Liverpool
NHS Liverpool Women's NHS Foundation Trust
University of Bristol

Food and drink during labour

During labour you can drink and eat what you want. There is no benefit to drinking more than normal but it may be beneficial to drink isotonic drinks rather than water. An example of an isotonic drink is a Lucozade sport.
There are other varieties available, but these drinks are unlikely to be provided by your place of birth.

If you receive opioids or have risk factors that make it more likely you will have a caesarean, it may be recommended to have a light diet as you may feel unwell or need anaesthetic.

It is important that your care is personal to you and suits any medical conditions you may have. It is important to speak to your midwives or doctors if a medical condition may impact what you can eat during labour.

Skin-to-skin

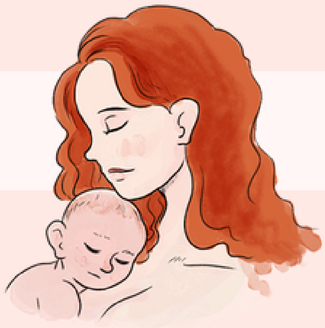

Skin-to-skin is where your baby is placed directly onto your skin or your birth partners bare skin after birth. This is encouraged as soon as possible after birth.

Skin-to-skin has been shown to promote the starting of breast feeding. It can help with bonding with you and your baby and can even impact baby and mum’s wellbeing following birth.

Skin-to-skin might not be possible for lots of reasons or may not be possible immediately. This may be because you may not be well enough and baby may not be in the best condition and may need some extra care. As soon as skin-to-skin is possible, you are more than welcome to take that opportunity.

Skin-to-skin can also be done with your birthing partner if you are not well enough and are happy with that. This also has benefits for your birthing partner and helps with bonding with your baby.

FUNDED BY NIHR | National Institute for Health and Care Research
UNIVERSITY OF LIVERPOOL
NHS Liverpool Women’s NHS Foundation Trust
University of BRISTOL

Where we got this information from

The Labour Process

Intrapartum care. NICE guidelines Published September 29, 2023. Accessed October 18, 2023. https://www.nice.org.uk/guidance/ng235/chapter/Recommendations#planning-place-of-birth

Hollowell J, Rowe R, Townend J, et al. The Birthplace in England national prospective cohort study: further analyses to enhance policy and service delivery decision-making for planned place of birth. Health Serv Deliv Res. 2015;3(36).

Pushing methods for the second stage of labour. doi:10.1002/14651858.CD009124.pub3

Chapman V, Charles C. The Midwife's Labour and Birth Handbook. Newark: John Wiley & Sons, Incorporated; 2013

Assisted vaginal birth (ventouse or forceps). Royal College of Obstetricians and Gynaecologists. https://www.rcog.org.uk/for-the-public/browse-our-patient-information/assisted-vaginal-birth-ventouse-or-forceps/

Possible procedures or interventions during labour

Intrapartum care. NICE guidelines Published September 29, 2023. Accessed October 18, 2023. https://www.nice.org.uk/guidance/ng235/chapter/Recommendations#planning-place-of-birth

Fetal monitoring in labour. NICE guidelines. Published December 14, 2022. Accessed October 20, 2023. https://www.nice.org.uk/guidance/ng229

Alfirevic Z, Devane D, Gyte GML, Cuthbert A. Continuous cardiotocography (CTG) as a form of electronic fetal monitoring (EFM) for fetal assessment during labour. Cochrane Database of Systematic Reviews 2017, Issue 2. Art. No.: CD006066. DOI: 10.1002/14651858.CD006066.pub3

Bohren MA, Hofmeyr GJ, Sakala C, Fukuzawa RK, Cuthbert A. Continuous support for women during childbirth. CDSR. 2017 July 16;(7).

Mobbs N, Williams C, Weeks A. Humanising birth: Does the language we use matter? The BMJ. Published February 8, 2018. Accessed November 28, 2023. https://blogs.bmj.com/bmj/2018/02/08/humanising-birth-does-the-language-we-use-matter/

Signs that labour has begun. nhs.uk. Published December 1, 2020. Accessed November 20, 2023. https://www.nhs.uk/pregnancy/labour-and-birth/signs-of-labour/signs-that-labour-has-begun/

Preterm labour and birth | Guidance | NICE. Published November 20, 2015. Accessed November 24, 2023. https://www.nice.org.uk/guidance/ng25

NHS Maternity Statistics, England, 2023-24. last accessed 10/03/25. Available from: https://digital.nhs.uk/data-and-information/publications/statistical/nhs-maternity-statistics/2023-24/births

Episiotomy. Royal College of Obstetricians and Gynaecologists. https://www.rcog.org.uk/for-the-public/perineal-tears-and-episiotomies-in-childbirth/episiotomy/

Fernado RJ, Sultan AH, et al on behalf of the Royal College of Obstetricians and Gynaecologists. The Management of Third-and-Fourth-Degree Perineal Tears. BJOG 2015

Murphy DJ, Strachan BK, Bahl R, on behalf of the Royal College of Obstetricians Gynaecologists. Assisted Vaginal Birth. BJOG 2020;127:e70–e112.

Caesarean birth. NICE guidelines. Published March 31, 2021. Accessed October 20, 2023. https://www.nice.org.uk/guidance/ng192

NMPA Project Team. National Maternity and Perinatal Audit: Clinical Report 2022. Based on births in NHS maternity services in England and Wales between 1 April 2018 and 31 March 2019. London: RCOG; 2022.

Assisted vaginal birth (ventouse or forceps). Royal College of Obstetricians and Gynaecologists. https://www.rcog.org.uk/for-the-public/browse-our-patient-information/assisted-vaginal-birth-ventouse-or-forceps/

Wyn Jones N, Mitchell EJ, Wakefield N, et al. Impacted fetal head during second stage Caesarean birth: A prospective observational study. European Journal of Obstetrics & Gynecology and Reproductive Biology. 2022;272:77-81. doi:10.1016/j.ejogrb.2022.03.004

Perineal tears and episiotomy. NHS inform. Accessed December 4, 2023. https://www.nhsinform.scot/ready-steady-baby/labour-and-birth/assisted-birth/perineal-tears-and-episiotomy/

FUNDED BY NIHR | National Institute for Health and Care Research   University of Liverpool   NHS Liverpool Women's NHS Foundation Trust   University of Bristol

Pain Relief

Intrapartum care. NICE guidelines Published September 29, 2023. Accessed October 18, 2023. https://www.nice.org.uk/guidance/ng235/chapter/Recommendations#planning-place-of-birth

Twin and Triplet pregnancy | Guidance | NICE. Published September 4, 2019. Accessed November 24, 2023. https://www.nice.org.uk/guidance/NG137

Opiate injections. Newcastle Hospitals NHS Foundation Trust. Published February 5, 2021. Accessed January 8, 2024. https://www.newcastle-hospitals.nhs.uk/services/maternity/labour-and-birth/pain-relief/opiate-injections/

Environment During Labour

Intrapartum care. NICE guidelines Published September 29, 2023. Accessed October 18, 2023. https://www.nice.org.uk/guidance/ng235/chapter/Recommendations#planning-place-of-birth

Fetal monitoring in labour. NICE guidelines. Published December 14, 2022. Accessed October 20, 2023. https://www.nice.org.uk/guidance/ng229

Assisted vaginal birth (ventouse or forceps). Royal College of Obstetricians and Gynaecologists. https://www.rcog.org.uk/for-the-public/browse-our-patient-information/assisted-vaginal-birth-ventouse-or-forceps/

Pushing methods for the second stage of labour. doi:10.1002/14651858.CD009124.pub3

Bohren MA, Hofmeyr GJ, Sakala C, Fukuzawa RK, Cuthbert A. Continuous support for women during childbirth. CDSR. 2017 July 16;(7).

Mobbs N, Williams C, Weeks A. Humanising birth: Does the language we use matter? The BMJ. Published February 8, 2018. Accessed November 28, 2023. https://blogs.bmj.com/bmj/2018/02/08/humanising-birth-does-the-language-we-use-matter

Possible Labour Complications

Intrapartum care. NICE guidelines Published September 29, 2023. Accessed October 18, 2023. https://www.nice.org.uk/guidance/ng235/chapter/Recommendations#planning-place-of-birth

Chandraharan E, Krishna A. Diagnosis and management of postpartum haemorrhage. BMJ. 2017;358:j3875. doi:10.1136/bmj.j3875

Intrapartum care for women with existing medical conditions or obstetric complications and their babies. Evidence reviews for pyrexia. NICE. Published online 2019.

Calman KC, Royston G. Personal paper: Risk language and dialects. British Medical Journal 1997;315:939–42.

Crofts J, Draycott TJ et al, on behalf of the Royal College of Obstetricians and Gynaecologists. Shoulder Dystocia. BJOG 2012
Chebsey CS, Fox R, et al on behalf of the Royal College of Obstetricians and Gynaecologists. Umbilical Cord Prolapse. BJOG 2014

Outcomes for the Baby

Intrapartum care. NICE guidelines Published September 29, 2023. Accessed October 18, 2023. https://www.nice.org.uk/guidance/ng235/chapter/Recommendations#planning-place-of-birth

Birthplace in England research programme | sheer | NPEU. Accessed October 18, 2023. https://www.npeu.ox.ac.uk/birthplace

Fetal monitoring in labour. NICE guidelines. Published December 14, 2022. Accessed October 20, 2023. https://www.nice.org.uk/guidance/ng229
Quality statement 7: Skin-to-skin contact |

Intrapartum care | Quality standards | NICE. Published December 10, 2015. Accessed October 20, 2023. https://www.nice.org.uk/guidance/qs105/chapter/quality-statement-7-skin-to-skin-contact

Postnatal care of the baby | Guidance | NICE. Published April 20, 2021. Accessed November 20, 2023. https://www.nice.org.uk/guidance/ng194/chapter/Recommendations#postnatal-care-of-the-baby

Lee AC, Cousens S, Wall SN, et al. Neonatal resuscitation and immediate newborn assessment and stimulation for the prevention of neonatal deaths: a systematic review, meta-analysis and Delphi estimation of mortality effect. BMC Public Health. 2011;11(3):S12. doi:10.1186/1471-2458-11-S3-S12

Experiences After Birth

Murphy DJ, Strachan BK, Bahl R, on behalf of the Royal College of Obstetricians Gynaecologists. Assisted Vaginal Birth. BJOG 2020;127:e70–e112.

Intrapartum care. NICE guidelines Published September 29, 2023. Accessed October 18, 2023. https://www.nice.org.uk/guidance/ng235/chapter/Recommendations#planning-place-of-birth
Early days. nhs.uk. Published December 8, 2020. Accessed November 20, 2023. https://www.nhs.uk/pregnancy/labour-and-birth/after-the-birth/early-days/

Fernado RJ, Sultan AH, et al on behalf of the Royal College of Obstetricians and Gynaecologists. The Management of Third-and-Fourth-Degree Perineal Tears. BJOG 2015

Postnatal care - Formula feeding information and support. https://www.nice.org.uk/guidance/ng194/evidence/t-formula-feeding-information-and-support-pdf-326764486011#:~:text=Breastfeeding%20is%20known%20to%20have,breast%20cancer%20in%20the%20mothers

Postnatal care of the baby | Guidance | NICE. Published April 20, 2021. Accessed November 20, 2023. https://www.nice.org.uk/guidance/ng194/chapter/Recommendations#postnatal-care-of-the-baby

Episiotomy. Royal College of Obstetricians and Gynaecologists. https://www.rcog.org.uk/for-the-public/perineal-tears-and-episiotomies-in-childbirth/episiotomy/

Depression - antenatal and postnatal: What else might it be? NICE. Accessed October 21, 2023. https://www.nice.org.uk/cks-uk-only

Antenatal and postnatal mental health: clinical management and service guidance | Guidance | NICE. Published December 17, 2014. Accessed November 24, 2023. https://www.nice.org.uk/guidance/cg192

Assisted vaginal birth (ventouse or forceps). Royal College of Obstetricians and Gynaecologists. https://www.rcog.org.uk/for-the-public/browse-our-patient-information/assisted-vaginal-birth-ventouse-or-forceps/
Caesarean birth. NICE guidelines. Published March 31, 2021. Accessed October 20, 2023. https://www.nice.org.uk/guidance/ng192

Postnatal care | Guidance | NICE. Published April 20, 2021. Accessed November 24, 2023. https://www.nice.org.uk/guidance/ng194

Pelvic floor dysfunction: prevention and non-surgical management | Guidance | NICE. Published December 9, 2021. Accessed November 24, 2023. https://www.nice.org.uk/guidance/ng210/chapter/Recommendations

Depression - antenatal and postnatal. NICE. Accessed November 20, 2023. https://www.nice.org.uk/cks-uk-only

Postnatal depression. nhs.uk. Published February 15, 2021. Accessed November 20, 2023. https://www.nhs.uk/mental-health/conditions/post-natal-depression/overview/

Postnatal depression | Royal College of Psychiatrists. www.rcpsych.ac.uk. Accessed November 20, 2023. https://www.rcpsych.ac.uk/mental-health/mental-illnesses-and-mental-health-problems/post-natal-depression

Postpartum psychosis for carers | Royal College of Psychiatrists. www.rcpsych.ac.uk. Accessed November 20, 2023. https://www.rcpsych.ac.uk/mental-health/mental-illnesses-and-mental-health-problems/postpartum-psychosis-in-carers

Antenatal care | Guidance | NICE. Published August 19, 2021. Accessed November 24, 2023. https://www.nice.org.uk/guidance/ng201

Urinary retention. NICE. Accessed November 20, 2023. https://www.nice.org.uk/bnf-uk-only

Supplementary information

Intrapartum care. NICE guidelines Published September 29, 2023. Accessed October 18, 2023. https://www.nice.org.uk/guidance/ng235/chapter/Recommendations#planning-place-of-birth

Eating and drinking in labour. doi:10.1002/14651858.CD003930.pub3

https://www.nice.org.uk/guidance/ng235/chapter/Recommendations#planning-place-of-birth Quality statement 7: Skin-to-skin contact | Intrapartum care | Quality standards | NICE. Published December 10, 2015. Accessed October 20, 2023.
Chapman V, Charles C. The Midwife's Labour and Birth Handbook. Newark: John Wiley & Sons, Incorporated; 2013

Signs that labour has begun. nhs.uk. Published December 1, 2020. Accessed November 20, 2023. https://www.nhs.uk/pregnancy/labour-and-birth/signs-of-labour/signs-that-labour-has-begun/
