# Supplementary material for: What Should Be Discussed When Considering a Vaginal Birth? A Delphi Consensus Study
Source: BJOG. 2025 Nov 18;133(3):520–31. doi: 10.1111/1471-0528.70071 (PMC12770075; doi:10.1111/1471-0528.70071)
Supplement: Supplementary file 7 — Table S1: Core Outcome Set‐STAndardised Protocol Items (COS‐STAP) Checklist. [file BJO-133-520-s015.pdf]

S1. Core Outcome Set-STAndardised Protocol Items (COS-STAP) Checklist for the 'Vaginal Birth Core Information Set' Study

Item Number	Name	Location
1	Identify in the title that the paper describes the background, methods and results for the development of a CIS	Title<br>Page 1
2	Provide a structured abstract	Abstract<br>Page 2-3
3	Describe the background and explain the rationale for developing the CIS	Background<br>Page 4-5
4	Describe the specific objectives with reference to developing a CIS	Background<br>Page 4-5
5	Describe the health condition(s) and population(s) that will be covered by the CIS	Methods<br>Page 5-10
6	Describe the intervention(s) that will be covered by the CIS	Methods<br>Page 5-10
7	Describe the setting(s) that will be covered by the CIS	Methods<br>Page 5-10
8	Indicate the CIS study registration details and registry name. If not yet registered indicate the intended registry	Methods<br>Page 5
9	Describe any study oversight committees	Acknowledgements<br>(Options Study Collaborative)<br>Page 19-21
10	Describe sources of funding, role of funders	Funding<br>Page 18
11	Describe any potential conflicts of interest within the study team and how these will be managed	Disclosures of interests<br>Page 21
12	Describe the stakeholder groups to be involved in the CIS development process and the rationale for their involvement	Methods- Page 5-10<br>Acknowledgements- Page 21
13	Describe the eligibility criteria for individuals from each stakeholder group	Methods<br>Page 5-10
14	Describe how individuals of each stakeholder groups will be identified	Methods<br>Page 5-10

15	Describe how individuals of each stakeholder group will be chosen from within the stakeholder group	Methods<br>Page 5-10
16	Describe how many planned individuals within each stakeholder group will be invited to participate in the consensus process	Methods<br>Page 5-10
17	Describe how individuals will be invited to take part in the consensus process	Methods<br>Page 8
18	Describe the information sources that will be used to identify the list of outcomes. Outline the methods or reference other protocols/papers.	Methods (Stage 1)<br>Page 5-7
19	Describe how outcomes may be dropped/ combined, with reasons	Methods- Page 9-10<br>Results- Page 13
20	Describe the methods to identify outcome descriptor terms	Methods (Stage 3)<br>Page 8
21	Describe the plans for how the consensus process will be undertaken	Methods (Stage 4)<br>Page 8-10
22	Describe what information will be presented to participants at the start of the consensus process	Methods (Stage 4)<br>Page 8
23	Describe what each participant will be asked to do at each stage of the consensus process	Methods (Stage 4)<br>Page 8
24	Describe how the participants will receive any feedback during the consensus process	Methods (Stage 4)<br>Page 9-10
25	Describe how non-response (or partial response) will be handled during the consensus process	Methods<br>Page 9
26	Describe how the study material will be made patient friendly and understandable (if relevant)	Methods (Think-aloud interviews)<br>Page 8
27	Describe the consensus definition	Methods<br>Page 9
28	Describe the procedure for determining how outcomes will be added/combined/dropped from consideration during the consensus process	Methods<br>Page 9
29	Describe how outcomes will be scored and summarised	Methods<br>Page 9-10

30	Describe how the response rate will be maximised	Methods<br>Page 8-9
31	Describe how attrition bias will be assessed	Results<br>Page 17
32	Describe any software that will be used during the consensus process and to analyse the results	Methods<br>Page 8
33	Describe any plans for obtaining research ethics committee / institutional review board approval in relation to the consensus process (if relevant)	Ethical approval<br>Page 21
34	Describe how informed consent will be obtained (if relevant)	Methods<br>Page 5-10
35	Describe any details about how the confidentiality of data collection will be preserved during the consensus process (if relevant)	Methods<br>Page 10
