# Supplementary material for: What Should Be Discussed When Considering a Vaginal Birth? A Delphi Consensus Study
Source: BJOG. 2025 Nov 18;133(3):520–31. doi: 10.1111/1471-0528.70071 (PMC12770075; doi:10.1111/1471-0528.70071)
Supplement: Supplementary file 8 — Table S2: DELPHISTAR checklist. [file BJO-133-520-s012.docx]

**S2.** DELPHISTAR checklist

| **Topic** | **Section** | **Item** | **Checklist Item** | **Location where item is reported** | **Exemplary wording** |
| --- | --- | --- | --- | --- | --- |
| **I**  **Title and Abstract** |  | 1 | Identification as a Delphi study in the title | Title page | What should be discussed when considering a vaginal birth? : A Delphi Consensus Study |
|  |  | 2 | Identification as a Delphi study in the abstract | Page 2; Abstract | A Delphi study was used to create the CIS. |
|  |  | 3 | Structured abstract | Page 2; Abstract | Abstract- Objective, design, setting, population, main outcome, results, conckusion |
| **II**  **Context** | **Formal** | 4 | Information about the sources of funding | Page 15; Funding | This Delphi study was funded by the Research England Policy Support Fund (PSF). |
|  |  | 5 | Information about the team of authors and/or researchers (e.g., discipline, institution) | Page 1; Authors | Author list (including affiliations) |
|  |  | 6 | Information about method consulting | Page 5; Methods | This study was registered with the Core Outcome Measures Effectiveness in Tests initiative (<https://comet-initiative.org/Studies/Details/2069>), and adheres to their recommended protocol and development standards and the COS-Standards for Reporting guidance (S1). |
|  |  | 7 | Information about the project background | Page 3-4; Introduction | Background of project and aims |
|  |  | 8 | Information about the study protocol | Page 5; Methods | The methodology was adapted from previously developed core outcome sets and CIS. The full protocol has been previously published.  Link: https://bmjopen.bmj.com/content/13/8/e070215 |
|  | **Content** | 9 | Justification of the chosen method (Delphi) to answer the research question | Page 5; Methods | The methodology was adapted from previously developed core outcome sets and core information sets. |
|  |  | 10 | Aim of the Delphi study (e.g., consensus, forecasting) | Page 2; Abstract & Page 4; Introduction | Abstract: *“We aimed to develop a Core Information Set (CIS) for vaginal birth.”*  Introduction: *“We aimed to define a CIS for vaginal birth, to provide women with consistent, patient-centred information to ensure they are well-informed about vaginal birth.”* |
| **III**  **Method** | **Body & Integration of knowledge** | 11 | Identification and elucidation of relevant expertise, spheres of experience, and perspectives (e.g., theory, practice, affected groups, disciplines) | Page 6–7; Methods, Stage 3 (The Delphi Survey), Participants | Stakeholders included antenatal and postnatal women, birth partners, healthcare professionals (obstetricians, midwives, anaesthetists, GPs, physiotherapists), medicolegal experts, researchers, and representatives from advocacy groups |
|  |  | 12 | Handling of knowledge, expertise and perspectives which are missing or have been deliberately not integrated | Not applicable | The stakeholder groups included capture the broad scope of knowledge and expertise in the area. No groups were excluded |
|  |  | 13 | Basic definition of expert | Page 6–7; Methods, Stage 3 | Professionals were defined as healthcare professionals, medicolegal experts or researchers working in reproductive health; parents were defined as antenatal/postnatal women, birth partners, or representatives from advocacy groups. |
|  | **Delphi variant and modifications** | 14 | Identification of the type of Delphi variant and potential modifications (e.g., classic Delphi, real-time Delphi, group Delphi) | Page 7; Stage 3: The Delphi Survey | The final survey was presented in a two-round modified Delphi process on REDCap. |
|  |  | 15 | Justification of the Delphi variant and modifications, including during the Delphi study, if applicable | Page 7; Methods, Stage 3 | A two-round modified Delphi was selected to optimise participation and minimise attrition, consistent with approaches used in other CIS developments. |
|  | **Sample of experts** | 16 | Selection criteria for the experts (per round, per expert group if applicable) | Page 6–7; Methods, Stage 3 | Stakeholders from across the UK were eligible if they were pregnant, had given birth, were a birth partner, healthcare professional, medicolegal professional, researcher, or advocate in maternity care. |
|  |  | 17 | Identification of the experts | Page 5–7; Methods, Stages 1–3 | Experts were identified through social media recruitment, stakeholder survey responses, and engagement with professional and advocacy organisations. |
|  |  | 18 | Information about recruiting and any subsequent recruiting of experts | Page 6; Methods, Stage 3 | Participants were recruited through online social media channels and invited to complete both survey rounds; round 1 participants who provided an email address were invited to round 2. |
|  | **Survey** | 19 | Elucidation of the content development for the questionnaire | Page 5–6; Methods, Stage 1–2 | Survey content was developed from systematic reviews, patient information leaflets, qualitative interviews with antenatal and postnatal women, and a stakeholder survey. |
|  |  | 20 | Description of the questionnaire (content and structure) | Page 6; Methods, Stage 2–3 | The Delphi survey contained 74 information items across 11 categories, rated on a 9-point Likert scale, with free-text boxes for comments. |
|  | **Delphi rounds** | 21 | Number of Delphi rounds | Page 7; Methods, Stage 3 | The final survey was presented in a two-round modified Delphi process. |
|  |  | 22 | Information about the aims of the individual Delphi rounds | Page 7; Methods, Stage 3 | Round 1 sought initial ratings of all items. Round 2 presented median scores and histograms from round 1 and asked participants to re-rate items. |
|  |  | 23 | Disclosure and justification of the criterion for discontinuation | Page 7; Methods, Stage 3 | A two-round process was pre-specified, consistent with other CIS studies, to balance rigour with feasibility and limit attrition. |
|  | **Feedback** | 24 | Information about what data was reported back per round | Page 7; Methods, Stage 3 | Participants were provided with their own score, the median score, and histograms of the distribution of scores for each group. |
|  |  | 25 | Information on how the results of the previous Delphi round were fed back to the experts surveyed (e.g., via frequencies, mean values, measures of dispersion, listing of comments) | Page 7; Methods, Stage 3 | Feedback was presented as numerical distributions and graphical histograms, differentiated by parent and professional groups. |
|  |  | 26 | Information on whether feedback was differentiated by specific groups (e.g., by field of expertise, institutional affiliation) | Page 7; Methods, Stage 3 | Yes, results were fed back separately for parents and professionals. |
|  |  | 27 | Information about how dissent and unclear results were handled | Page 8; Methods, Stage 4 | Items without consensus were carried forward to the consensus meetings for further discussion and voting. |
|  | **Data analysis** | 28 | Disclosure of the quantitative and qualitative analytical strategy | Page 6–7; Methods, Stage 1–3 | Quantitative items were analysed descriptively. Free-text responses and interview transcripts were analysed thematically using Braun and Clarke’s approach. |
|  |  | 29 | Definition and measurement of consensus | Page 7; Methods, Stage 3 | Consensus was defined as percentage agreement, meaning that agreement was assumed if at least 80% of the respondents agreed on an item. |
|  |  | 30 | Information on group-specific analysis or weighting of experts (e.g., theory vs. practice, discipline-specific analysis) | Page 7; Methods, Stage 3 | Analysis was stratified by stakeholder group (parents/non-professionals and professionals), with results presented separately for each group. Equal weighting was given to both groups. |
| **IV**  **Results** | **Delphi process** | 31 | Illustration of the Delphi study (e.g., in a flow chart) | Page 12; Results, Stage 4 / Figures | flow diagram (Figure 1) illustrates the Delphi process from item identification to consensus meetings and final CIS development. |
|  |  | 32 | Information about special aspects during the Delphi study (e.g., deviations from the intended approach with justification) | Page 11; Results, Stage 4 | Two consensus meetings were held instead of one due to the number of items requiring discussion, to allow adequate time for participation and decision-making. |
|  |  | 33 | Number of experts per round (both invited and participating) | Page 9; Results, Stage 3 | 631 participants completed round 1 (533 patients, 98 professionals). 288 participants completed round 2 (214 patients, 74 professionals), representing an attrition rate of 45.2%. |
|  | **Results** | 34 | Presentation of the results for each Delphi round and the final results | Page 10–11; Results, Stage 3–4 | No items met exclusion criteria after round 1, and three new items were suggested. In round 2, 29 items reached the threshold for discussion. After consensus meetings and merging, 19 final items were agreed across seven domains. |
| **V Discussion** | **Quality of findings** | 35 | Highlighting the findings from the Delphi study | Page 13; Discussion, Main Findings | The final CIS contains 19 key information points, grouped into seven domains, to support informed decision-making about vaginal birth. |
|  |  | 36 | Validity of the results (e.g., transferability of the findings) | Page 14; Discussion, Main Findings | Although developed in the UK, the CIS can be adapted for other settings, with careful tailoring to local statistics and cultural context. |
|  |  | 37 | Reliability of the results (e.g., split half, inter-rater reliability) | Page 14; Discussion, Strengths and Limitations | The iterative process involved more than 600 stakeholders, with consistent representation of patient and professional voices across rounds, supporting the reliability of the findings. |
|  |  | 38 | Reflection on potential limitations (e.g., number of experts, response bias) | Page 14–15; Discussion, Strengths and Limitations | Attrition between rounds was 45.2%, higher than anticipated, and recruitment via social media may favour more digitally engaged participants. Nonetheless, broad representation was achieved. |
