# Supplementary material for: What Should Be Discussed When Considering a Vaginal Birth? A Delphi Consensus Study
Source: BJOG. 2025 Nov 18;133(3):520–31. doi: 10.1111/1471-0528.70071 (PMC12770075; doi:10.1111/1471-0528.70071)
Supplement: Supplementary file 9 — Table S3: Pregnancy related demographics. [file BJO-133-520-s007.docx]

S7. Pregnancy related demographics of participants for Round 1 and 2

|  | **Round 1** | | **Round 2** | |
| --- | --- | --- | --- | --- |
|  | ***Pregnant at time of survey (n= 144)*** | | ***Pregnant at time of survey (n= 42)*** | |
| **Parity** | **N (%)** | | **N (%)** | |
| Primiparous | 123 (85.4) | | 34 (81.0) | |
| Multiparous | 21 (14.6) | | 8 (19.0) | |
| **Gestation** | **Median** | **Range** | **Median** | **Range** |
| *Mean gestation in weeks (range)* | 14 | 4-39 | 15 | 4-39 |
|  | ***Previously been pregnant (n=335)*** | | ***Previously been pregnant (n=179)*** | |
| **How many children?** | **N (%)** | | **N (%)** | |
| 1 | 173 (51.6) | | 103 (57.5) | |
| 2 | 123 (36.7) | | 51 (28.5) | |
| 3 | 25 (7.5) | | 17 (9.5) | |
| 4 | 9 (2.7) | | 6 (3.4) | |
| ≥5 | 5 (1.5) | | 2 (1.1) | |
| Prefer not to say | 0 (0.0) | | 0 (0.0) | |
| **How long ago did (most recent) birth occur?** | **N (%)** | | **N (%)** | |
| In last 6 months | 42 (12.5) | | 19 (10.6) | |
| In last 6-12 months | 87 (26.0) | | 46 (25.7) | |
| 1-3 years ago | 110 (32.8) | | 60 (33.5) | |
| 3-5 years ago | 48 (14.3) | | 13 (7.3) | |
| 5-10 years ago | 30 (9.0) | | 21 (11.7) | |
| 10-20 years ago | 12 (3.6) | | 13 (7.3) | |
| >20 years ago | 6 (1.8) | | 7 (3.9) | |
| **Mode of birth experienced** | **N (%)** | | **N (%)** | |
| Vaginal birth | 262 (78.2) | | 152 (84.9) | |
| Assisted vaginal birth  (forceps, ventouse Kiwi, silastic) | 50 (14.9) | | 32 (17.9) | |
| Emergency caesarean section | 38 (11.3) | | 16 (8.9) | |
| Elective caesarean section | 30 (9.0) | | 11 (6.1) | |
| Prefer not to say | 1 (0.3) | | 0 (0.0) | |
