# Supplementary material for: What Should Be Discussed When Considering a Vaginal Birth? A Delphi Consensus Study
Source: BJOG. 2025 Nov 18;133(3):520–31. doi: 10.1111/1471-0528.70071 (PMC12770075; doi:10.1111/1471-0528.70071)
Supplement: Supplementary file 10 — Table S4: Delphi survey round 1 scoring. [file BJO-133-520-s006.docx]

**S8.** Delphi Survey round 1 scoring

| Information item | % of all participants scoring as 'Limited importance' | % of all participants who scored as ‘Critical’ | % of patients who scored as 'Limited importance' | % of patients who scored as 'Critical' | % of professionals who scored as 'Limited importance' | % of professionals who scored as 'Critical' (%) |
| --- | --- | --- | --- | --- | --- | --- |
| Keeping mobile and adopting different positions in labour | 8.23% | 62.10% | 8.37% | 60.46% | 6.52% | 69.89% |
| Information about being in water during labour and birth | 8.39% | 55.16% | 8.37% | 55.70% | 8.70% | 47.31% |
| Medical professionals who may be present in the room during labour | 5.32% | 65.16% | 5.89% | 63.88% | 2.17% | 69.89% |
| Labour companions who you can choose to have present during labour and their role in the process | 8.23% | 64.03% | 9.13% | 62.93% | 3.26% | 67.74% |
| Atmosphere and environment during labour | 6.94% | 54.68% | 7.03% | 56.84% | 5.43% | 43.01% |
| Items that may be needed when in labour | 5.48% | 57.90% | 4.94% | 60.65% | 8.70% | 40.86% |
| Eating and drinking in labour- What food or drink can be consumed; When can it be consumed or not?. | 6.13% | 59.52% | 6.27% | 60.08% | 5.43% | 54.84% |
| Birth locations: Choice of where to give birth (home, midwife led unit, consultant led unit) | 5.48% | 70.65% | 6.08% | 67.49% | 2.17% | 87.10% |
| Transfer of location during labour | 5.81% | 71.29% | 6.27% | 69.01% | 3.26% | 81.72% |
| The different stages of labour and birth | 6.94% | 59.68% | 6.65% | 59.70% | 7.61% | 58.06% |
| The signs and symptoms of labour | 5.65% | 71.29% | 5.70% | 69.01% | 4.35% | 81.72% |
| Expected progress during labour | 5.97% | 65.00% | 5.70% | 64.83% | 6.52% | 63.44% |
| What happens when waters break before labour | 5.32% | 66.94% | 5.32% | 68.06% | 4.35% | 59.14% |
| The effect a baby's position (which way the baby is facing) can have on labour and experience | 5.97% | 62.10% | 5.32% | 65.59% | 7.61% | 40.86% |
| Fluid intake during labour and urinating during labour | 5.81% | 58.71% | 4.94% | 59.51% | 9.78% | 52.69% |
| Giving birth to the placenta | 5.48% | 64.35% | 5.13% | 64.45% | 5.43% | 62.37% |
| Expected experiences whilst pushing during labour, when about to give birth | 6.45% | 66.77% | 6.46% | 66.92% | 5.43% | 64.52% |
| Expected experiences immediately following birth | 5.16% | 71.61% | 4.75% | 70.34% | 5.43% | 77.42% |
| Use of non-medical pain relief during labour | 5.97% | 61.13% | 5.89% | 60.27% | 5.43% | 62.37% |
| Use of simple medical pain relief during labour | 4.52% | 69.84% | 4.56% | 68.25% | 2.17% | 76.34% |
| Use of epidural during labour | 5.48% | 69.35% | 5.89% | 68.25% | 2.17% | 72.04% |
| Moderate but common complications relating to the mother during labour | 6.94% | 59.03% | 6.65% | 60.27% | 6.52% | 51.61% |
| Moderate but common complications relating to the baby during labour | 6.77% | 65.81% | 6.65% | 66.54% | 3.26% | 61.29% |
| Severe but common complications related to vaginal bleeding during labour | 5.81% | 63.23% | 6.08% | 64.64% | 1.09% | 54.84% |
| Severe but uncommon complications relating to the baby during labour | 7.42% | 63.55% | 7.22% | 66.54% | 5.43% | 46.24% |
| Severe but rare complications for mother and baby during labour | 8.71% | 56.29% | 7.79% | 61.79% | 11.96% | 24.73% |
| Serious illness for mother that may result in a long-term hospital stay and/or possible long-term consequences (severe but very rare) | 11.94% | 56.29% | 9.70% | 62.55% | 22.83% | 20.43% |
| Maternal death (very rare) | 14.19% | 53.39% | 10.65% | 59.70% | 32.61% | 17.20% |
| Vaginal examinations offered during labour | 4.19% | 64.52% | 4.18% | 63.31% | 3.26% | 69.89% |
| How a baby's wellbeing is monitored during labour | 5.48% | 67.90% | 5.89% | 66.16% | 2.17% | 75.27% |
| Procedures to investigate baby's wellbeing during labour when there are concerns with the monitoring | 5.00% | 60.65% | 5.13% | 61.98% | 3.26% | 51.61% |
| The process of speeding up labour (augmentation of labour) | 4.52% | 64.68% | 4.56% | 65.97% | 3.26% | 55.91% |
| When an assisted vaginal birth may be offered or recommended | 5.16% | 67.90% | 5.32% | 66.73% | 2.17% | 70.97% |
| When an episiotomy may be offered | 4.03% | 67.74% | 4.37% | 67.30% | 1.09% | 68.82% |
| When a caesarean section may be offered | 4.68% | 69.68% | 4.94% | 68.63% | 2.17% | 73.12% |
| When intravenous antibiotics may be recommended during labour | 4.84% | 55.65% | 4.56% | 58.56% | 5.43% | 37.63% |
| When a blood sample or drip (intravenous line) may be needed | 5.48% | 55.16% | 5.13% | 57.60% | 6.52% | 38.71% |
| Umbilical cord cutting | 5.81% | 57.10% | 5.32% | 57.98% | 7.61% | 50.54% |
| Methods to reduce risk of serious tears to the vagina | 4.03% | 67.58% | 3.99% | 67.30% | 3.26% | 66.67% |
| Examination of the vagina and the rectum following birth | 5.65% | 62.42% | 5.89% | 62.55% | 3.26% | 59.14% |
| Repair of tears with stitches | 5.00% | 66.77% | 4.94% | 67.49% | 4.35% | 61.29% |
| Manual removal of placenta | 7.26% | 55.65% | 6.08% | 60.84% | 11.96% | 23.66% |
| What is done when bleeding after birth is more than the usual | 5.81% | 60.97% | 5.32% | 64.83% | 7.61% | 36.56% |
| Transfer to different area of care due to concerns for mother's health | 5.65% | 58.87% | 4.75% | 61.60% | 8.70% | 40.86% |
| Symptoms that may be experienced following birth | 3.87% | 63.71% | 3.99% | 64.83% | 2.17% | 55.91% |
| Pelvic floor injury that can happen during labour | 5.16% | 62.74% | 5.13% | 64.26% | 4.35% | 51.61% |
| Length of stay in unit or hospital following birth | 4.68% | 57.26% | 4.75% | 59.70% | 2.17% | 40.86% |
| Retained tissue or placenta following birth | 5.97% | 55.65% | 4.94% | 61.41% | 10.87% | 21.51% |
| Issues with the perineum following birth | 5.32% | 62.26% | 5.32% | 65.02% | 4.35% | 44.09% |
| Maternal infection requiring antibiotics following birth | 6.77% | 53.39% | 5.70% | 58.17% | 11.96% | 23.66% |
| Bowel or bladder symptoms following birth | 4.84% | 62.90% | 4.75% | 64.64% | 4.35% | 51.61% |
| Serious maternal health conditions following birth that require medical treatment | 6.94% | 60.65% | 5.70% | 64.45% | 13.04% | 35.48% |
| Possible mental health experiences following vaginal birth | 3.87% | 70.97% | 3.61% | 71.10% | 4.35% | 65.59% |
| Long term back pain | 8.55% | 49.52% | 5.70% | 55.13% | 23.91% | 16.13% |
| Future pregnancies and birth experiences | 5.32% | 59.35% | 4.37% | 61.03% | 9.78% | 45.16% |
| Pelvic floor and genital tract issues | 6.61% | 59.19% | 4.56% | 62.55% | 16.30% | 38.71% |
| Changes related to sexual health function after birth | 4.52% | 58.87% | 3.61% | 62.36% | 8.70% | 37.63% |
| Long term effects of childbirth on mental health | 5.00% | 62.42% | 3.80% | 65.97% | 9.78% | 40.86% |
| Effects of childbirth on social health | 5.16% | 60.48% | 4.37% | 63.12% | 8.70% | 44.09% |
| Skin to skin following birth | 5.32% | 60.48% | 5.51% | 59.89% | 3.26% | 62.37% |
| Feeding of the baby following birth | 4.84% | 65.97% | 4.75% | 64.64% | 3.26% | 69.89% |
| Attachment of the baby following birth to the mother | 4.35% | 60.00% | 4.37% | 59.89% | 3.26% | 56.99% |
| Impact on baby's immune system | 7.26% | 59.03% | 7.03% | 61.41% | 7.61% | 43.01% |
| Condition of baby when they are born | 5.97% | 62.58% | 6.27% | 64.64% | 3.26% | 48.39% |
| Transmission of bloodborne viruses to baby | 7.90% | 61.45% | 6.65% | 65.02% | 14.13% | 38.71% |
| Length of hospital stay for the baby | 5.65% | 56.45% | 4.94% | 60.46% | 7.61% | 33.33% |
| Admission of baby to special care or neonatal intensive care unit (SCBU, NICU) | 6.77% | 58.55% | 6.46% | 62.36% | 7.61% | 33.33% |
| Birth trauma to baby during birth | 6.94% | 59.84% | 5.70% | 64.07% | 13.04% | 34.41% |
| Serious conditions that may affect baby in the short or long term | 7.74% | 58.06% | 6.65% | 62.17% | 13.04% | 33.33% |
| Physical impact on life of baby | 9.19% | 57.90% | 6.84% | 62.74% | 21.74% | 25.81% |
| Birth partner wellbeing following birth | 6.13% | 51.77% | 5.32% | 54.94% | 9.78% | 32.26% |
| Financial cost to family following birth | 9.19% | 49.35% | 6.65% | 53.99% | 22.83% | 22.58% |
| Hospital conditions can affect labour experience | 5.65% | 55.81% | 4.94% | 57.98% | 8.70% | 40.86% |
| Financial cost to health service | 20.97% | 46.29% | 15.97% | 51.71% | 47.83% | 12.90% |
