# Supplementary material for: What Should Be Discussed When Considering a Vaginal Birth? A Delphi Consensus Study
Source: BJOG. 2025 Nov 18;133(3):520–31. doi: 10.1111/1471-0528.70071 (PMC12770075; doi:10.1111/1471-0528.70071)
Supplement: Supplementary file 11 — Table S5: Summary information item scoring for survey round 2 and inclusion or exclusion in consensus meeting. [file BJO-133-520-s011.docx]

S9. *Summary of information item scoring for survey round 2 and inclusion or exclusion in consensus meeting*

| Information item | % professionals scored critical (7-9) | % patients scored as critical (7-9) | Median | In or out of consensus meeting | Automatically included in CIS |
| --- | --- | --- | --- | --- | --- |
| Keeping mobile and adopting different positions in labour | 82.3% | 83.0% | 8 | In | No |
| Information about being in water during labour and birth | 50.6% | 68.6% | 7 | Out | No |
| Medical professionals who may be present in the room during labour | 78.5% | 81.3% | 7 | In | No |
| Labour companions who you can choose to have present during labour and their role in the process | 85.0% | 82.9% | 8 | In | No |
| Atmosphere and environment during labour | 50.0% | 63.7% | 7 | Out | No |
| Items that may be needed when in labour | 47.5% | 73.2% | 7 | Out | No |
| Eating and drinking in labour- What food or drink can be consumed; When can it be consumed or not?. | 83.5% | 75.4% | 7 | In | No |
| Birth locations: Choice of where to give birth | 97.5% | 87.8% | 8 | In | Yes |
| Transfer of location during labour | 92.4% | 85.7% | 8 | In | Yes |
| The different stages of labour and birth | 78.5% | 80.4% | 7 | In | No |
| The signs and symptoms of labour | 98.7% | 90.4% | 8 | In | Yes |
| Expected progress during labour | 84.8% | 82.2% | 7 | In | No |
| What happens when waters break before labour | 86.1% | 87.9% | 7 | In | No |
| The effect a baby's position can have on labour and experience | 51.3% | 75.1% | 7 | Out | No |
| Fluid intake during labour and urinating during labour | 72.2% | 73.6% | 7 | Out | No |
| Giving birth to the placenta | 76.3% | 83.3% | 7 | In | No |
| Expected experiences whilst pushing during labour, when about to give birth | 87.3% | 84.8% | 8 | In | No |
| Expected experiences immediately following birth | 93.6% | 88.3% | 8 | In | Yes |
| Use of non-medical pain relief during labour | 78.2% | 78.7% | 7 | Out | No |
| Use of simple medical pain relief during labour | 92.2% | 87.4% | 8 | In | Yes |
| Use of epidural during labour | 92.3% | 87.4% | 8 | In | Yes |
| Moderate but common complications relating to the mother during labour | 75.6% | 83.0% | 7 | In | No |
| Moderate but common complications relating to the baby during labour | 82.3% | 85.7% | 7 | In | No |
| Severe but common complications related to vaginal bleeding during labour | 83.5% | 84.2% | 7 | In | No |
| Severe but uncommon complications relating to the baby during labour | 74.7% | 82.7% | 7 | In | No |
| Severe but rare complications for mother and baby during labour | 31.7% | 62.8% | 7 | Out | No |
| Serious illness for mother that may result in a long-term hospital stay and/or possible long-term consequences (severe but very rare) | 21.5% | 60.9% | 6 | Out | No |
| Maternal death (very rare) | 17.7% | 60.4% | 6 | Out | No |
| Vaginal examinations offered during labour | 89.7% | 84.1% | 8 | In | No |
| How a baby's wellbeing is monitored during labour | 94.9% | 90.6% | 8 | In | Yes |
| Procedures to investigate baby's wellbeing during labour when there are concerns with the monitoring | 79.2% | 84.1% | 7 | In | Yes* |
| The process of speeding up labour (augmentation) | 77.2% | 82.9% | 7 | In | No |
| When an assisted vaginal birth may be offered or recommended | 92.5% | 85.0% | 8 | In | Yes |
| When an episiotomy may be offered | 89.9% | 84.5% | 8 | In | No |
| When a caesarean section may be offered | 94.9% | 87.1% | 8 | In | Yes |
| When intravenous antibiotics may be recommended during labour | 48.1% | 67.4% | 7 | Out | No |
| When a blood sample or drip (intravenous line) may be needed | 37.2% | 63.2% | 7 | Out | No |
| Umbilical cord cutting | 72.2% | 76.7% | 7 | Out | No |
| Methods to reduce risk of serious tears to the vagina | 83.5% | 88.0% | 8 | In | No |
| Examination of the vagina and the rectum following birth | 84.8% | 80.5% | 7 | In | No |
| Repair of tears with stitches | 88.3% | 83.6% | 7 | In | No |
| Manual removal of placenta | 39.7% | 68.9% | 7 | Out | No |
| What is done when bleeding after birth is more than the usual | 59.0% | 74.9% | 7 | Out | No |
| Transfer to different area of care due to concerns for mother's health | 60.3% | 77.3% | 7 | Out | No |
| Symptoms that may be experienced following birth | 84.4% | 88.5% | 8 | In | Yes* |
| Pelvic floor injury that can happen during labour | 80.8% | 85.9% | 7 | In | No |
| Length of stay in unit or hospital following birth | 59.7% | 73.7% | 7 | Out | No |
| Retained tissue or placenta following birth | 30.4% | 71.7% | 7 | Out | No |
| Issues with the perineum following birth | 69.6% | 82.6% | 7 | In | No |
| Maternal infection requiring antibiotics following birth | 32.9% | 72.3% | 7 | Out | No |
| Bowel or bladder symptoms following birth | 75.9% | 84.3% | 7 | In | No |
| Serious maternal health conditions following birth that require medical treatment | 53.2% | 74.9% | 7 | Out | No |
| Possible mental health experiences following vaginal birth | 91.1% | 91.6% | 8 | In | Yes |
| Long term back pain | 20.3% | 56.5% | 6 | Out | No |
| Future pregnancies and birth experiences | 67.1% | 74.9% | 7 | Out | No |
| Pelvic floor and genital tract issues | 63.3% | 73.8% | 7 | Out | No |
| Changes related to sexual health function after birth | 58.2% | 70.7% | 7 | Out | No |
| Long term effects of childbirth on mental health | 65.8% | 82.2% | 7 | In | Yes |
| Effects of childbirth on social health | 70.9% | 75.4% | 7 | Out | No |
| Skin to skin following birth | 91.1% | 88.5% | 8 | In | No |
| Feeding of the baby following birth | 100.0% | 89.5% | 8 | In | Yes |
| Attachment of the baby following birth to the mother | 84.8% | 83.8% | 8 | In | No |
| Impact on baby's immune system | 59.5% | 78.5% | 7 | Out | No |
| Condition of baby when they are born | 87.3% | 85.9% | 7 | In | No |
| Transmission of bloodborne viruses to baby | 45.6% | 72.8% | 7 | Out | No |
| Length of hospital stay for the baby | 44.3% | 67.5% | 7 | Out | No |
| Admission of baby to special care or neonatal intensive care unit (SCBU, NICU) | 43.0% | 68.6% | 7 | Out | No |
| Birth trauma to baby during birth | 45.6% | 70.2% | 7 | Out | No |
| Serious conditions that may affect baby in the short or long term | 43.0% | 68.4% | 7 | Out | No |
| Physical impact on life of baby | 26.6% | 64.9% | 7 | Out | No |
| Birth partner wellbeing following birth | 48.1% | 66.8% | 7 | Out | No |
| Financial cost to family following birth | 30.4% | 58.9% | 7 | Out | No |
| Hospital conditions can affect labour experience | 72.1% | 78.4% | 7 | Out | No |
| Financial cost to health service | 12.7% | 48.4% | 5 | Out | No |
| Family planning following birth | 78.5% | 64.0% | 7 | Out | No |
| Benefits of a vaginal birth compared to other modes of birth | 83.5% | 76.3% | 7 | Out | No |
| After care in the immediate hours following a vaginal birth | 69.6% | 88.4% | 7 | Out | No |

*Item did not meet criteria for automatic inclusion on its own but merged with item that did. During consensus meeting discussions, was agreed to automatically include the new merged item.
