# Supplementary material for: What Should Be Discussed When Considering a Vaginal Birth? A Delphi Consensus Study
Source: BJOG. 2025 Nov 18;133(3):520–31. doi: 10.1111/1471-0528.70071 (PMC12770075; doi:10.1111/1471-0528.70071)
Supplement: Supplementary file 12 — Table S6: Information items included automatically in the core information set following the second Delphi survey round. [file BJO-133-520-s001.docx]

S10. Information items included automatically included

| Information item​ |
| --- |
| Choice of where to give birth (home, midwife led unit, consultant led unit), and when and why may it be recommended to change location during labour.​ |
| Symptoms of labour.​ |
| Use of medical pain relief in labour inc. gas & air, oral meds (I.e. paracetamol, dihydrocodeine), injectable meds (I.e. pethidine, diamorphine) & epidural.​ |
| When an assisted vaginal birth or caesarean birth may be offered or recommended, and why.​ |
| How a baby’s wellbeing is checked during labour- Monitoring and procedures.​ |
| Potential experiences or symptoms immediately following birth.​ |
| Possible mental health experiences following birth (may be in short and long term).​ |
| Feeding of the baby following birth.​ |
