# Supplementary material for: What Should Be Discussed When Considering a Vaginal Birth? A Delphi Consensus Study
Source: BJOG. 2025 Nov 18;133(3):520–31. doi: 10.1111/1471-0528.70071 (PMC12770075; doi:10.1111/1471-0528.70071)
Supplement: Supplementary file 13 — Table S7: Information on merging of information items. [file BJO-133-520-s005.docx]

S11. Information items merged following Delphi rounds

| Information item | Information item merged into | New information item descriptive stem | Rationale for merging during consensus meeting discussions |
| --- | --- | --- | --- |
| The process of speeding up labour (augmentation of labour) | How the stages of labour are defined, and the expected progress through these | -Stages of labour including latent, first, second & third stage, transition.  -What is considered normal timing of progress?  -When intervention to help with progress may be offered if not progressing as expected? I.e. Labour augmentation (what this is, how it is done, risks and benefits). | The process of augmentation was felt to be linked to the expected progress. If progress is not expected, then augmentation may be considered or recommended to the person in labour. This is clarified in the information item’s updated stem. |
| How the stages of labour are defined, and the expected progress through these |  |  |  |
| Birth locations: Choice of where to give birth | Choice of where to give birth (home, midwife led unit, consultant led unit), and when & why may it be recommended to change location during labour. | Factors that may affect where it is recommended that someone give birth (or not give birth).  Which medical professionals will be present depending on location? | Discussions highlighted that choice of birth location and the possibility of transfer are strongly linked. The information is clearer when combined, as transfer is often a consequence of clinical need or preference change during labour. |
| Transfer of location during labour |  |  |  |
| Skin-to-skin and attachment of the baby following birth. | Skin-to-skin and attachment of the baby following birth. | Skin-to-skin is the act of putting baby directly onto skin of mother or birth partner following birth.  How soon after birth can this happen, factors that can affect this etc. · How having a vaginal birth may impact bonding of the parents with the baby. | Attachment and skin-to-skin were felt to overlap, as both describe early bonding practices after birth. Merging avoids duplication and provides a more coherent explanation of immediate postnatal care. |
| Attachment of the baby following birth to the mother |  |  |  |
| Moderate but common complications relating to the baby during labour | Complication related to the baby during labour. | Moderate but common including- Baby doing a poo inside the womb; Concerns about the baby's heart rate; Uterine hyperstimulation distressing the baby.  Severe but uncommon including- Cord prolapse (the baby's cord coming out of the vagina before the baby is born), shoulder dystocia (the baby's shoulders getting stuck after the head is born); Chorioamnionitis- infection affecting the womb and the baby. | The group agreed that complications for the baby should be covered together, distinguishing between more common and rarer, severe scenarios. This gives a single comprehensive item while still clarifying levels of risk. |
| Severe but uncommon complications relating to the baby during labour |  |  |  |
| Moderate but common complications relating to the mother during labour | Complications relating to the mother during labour. | Moderate or severe, but common, complications e.g. Mother having a temperature; Changes in vital parameters (blood pressure, heart rate, urine output); Uterine hyperstimulation (when the womb gets too many contractions in a short space of time).  Complications related to bleeding e.g. Bleeding during labour (antepartum haemorrhage); Placental abruption- when the placenta separates from the womb before birth of the baby. | It was felt that maternal complications should be presented as a combined item, as they often overlap and can escalate in severity. The merged stem better reflects the range of complications and their management. |
| Severe but common complications related to vaginal bleeding during labour |  |  |  |
| Pelvic floor injury that can happen during labour | Pelvic floor injury that can happen during labour, examination of the area to assess these, and potential issues with this area following birth | -Injuries to the pelvic floor include grazes, bruising or tears to the vagina (different degrees- 1st, 2nd, 3rd, 4th); Tears to the neck of the womb. These may require stitches (may be recommended by midwife or doctor).  -Following birth may experience (in the perineal area)- Pain; Change in sensation; Wound breakdown e.g. stitches unravelling; Infection (may or may not require antibiotics).  -Some people may experience short- or long-term bladder or bowel issues (e.g. pain, incontinence). | Bladder or bowel symptoms which can occur following birth are due to stress or injury of the pelvic floor, and so it was decided to merge this with the pelvic floor injury information item. The item description explains this in more detail. |
| Bowel or bladder symptoms following birth |  |  |  |
| How a baby's wellbeing is monitored during labour | How a baby’s wellbeing is checked during labour- Monitoring and procedures. | -Monitoring includes listening to heartbeat with a doppler, continuous monitoring (CTG, STAN), small clip attached to baby's head (fetal scalp electrode).  -Procedures to check wellbeing include- Fetal scalp stimulation (touching baby's head during a vaginal examination), fetal blood sampling (taking a small sample of blood from baby's head). | Both items describe ways of assessing the baby’s wellbeing. The consensus was to merge them, so that monitoring and follow-up procedures are explained together in a logical sequence. |
| Procedures to investigate baby's wellbeing during labour when there are concerns with the monitoring |  |  |  |
| Use of simple medical pain relief during labour | Use of medical pain relief in labour including gas & air, oral medications (i.e. paracetamol, dihydrocodeine), injectable medications (i.e. pethidine, diamorphine) & epidural. | -When they will be offered, when it is not possible to have, possible labour consequences.  -Epidural is a patient-controlled pain relief which numbs from the bottom of the chest to the toes. It is put in by a doctor with an injection of local anaesthetic into the back. | Epidural analgesia is a type of medical pain relief, and it was felt that this was important to include along with the oral or intramuscular medications, so this was merged into a new item. |
| Use of epidural during labour |  |  |  |
| When an assisted vaginal birth may be offered or recommended | When an assisted vaginal birth or caesarean birth may be offered or recommended, and why.​ | -Assisted vaginal birth includes forceps (metal spoons on baby's head) or ventouse (suction cup on baby's head) when the neck of the womb is fully open. A caesarean birth (also known as a C-section) is an operation whereby the baby is born through a cut into the abdomen and womb. | These were merged to give a clear overview of the circumstances in which assisted or surgical births may be offered. The combined item provides balanced information on both approaches, which are often considered together during discussions of birth options. |
| When a caesarean section may be offered |  |  |  |
| Expected experiences immediately following birth | Potential experiences or symptoms immediately following birth.​ | -E.g. Pain (from tears or after pains from womb contracting); Bleeding; Exhaustion; Anxiety; Urinary incontinence; Reduced amounts or urinating. | The group felt these were essentially describing the same content. Merging avoids repetition and ensures all immediate post-birth experiences are presented in one place. |
| Symptoms that may be experienced following birth |  |  |  |
| Possible mental health experiences following vaginal birth | Possible mental health experiences following birth (may be in short and long term). | E.g. Baby blues; Post-traumatic stress disorder; Effect on self-esteem; Postnatal depression; Mental health problems following perineal tear; Fears for future pregnancy/birth. | It was agreed that mental health effects should not be separated into short- and long-term, as experiences often overlap. The merged item provides a clearer narrative of the range of potential psychological impacts. |
| Long term effects of childbirth on mental health |  |  |  |
