# Supplementary material for: What Should Be Discussed When Considering a Vaginal Birth? A Delphi Consensus Study
Source: BJOG. 2025 Nov 18;133(3):520–31. doi: 10.1111/1471-0528.70071 (PMC12770075; doi:10.1111/1471-0528.70071)
Supplement: Supplementary file 14 — Table S8: Voting on information items during consensus meetings and outcomes. [file BJO-133-520-s016.docx]

S11. Voting on information items during consensus meetings and outcomes

Key

Include in core information set

Exclude

Merge into another point (more information with * below)

Include supplementary information list

|  | Round 1 of voting | | | | | Round 2 of voting (no consensus in R1) | | | | | Round 3 of voting (no consensus in R2) | | | | | Round 4 of voting (no consensus R3) | | | |  |
| --- | --- | --- | --- | --- | --- | --- | --- | --- | --- | --- | --- | --- | --- | --- | --- | --- | --- | --- | --- | --- |
| Information item | Include | Exclude | Unsure | Merge | Include supp. | Include | Exclude | Unsure | Merge | Include supp. | Include | Exclude | Unsure | Merge | Include supp. | Include | Exclude | Unsure | Merge | Include supp. |
| Labour process |  |  |  |  |  |  |  |  |  |  |  |  |  |  |  |  |  |  |  |  |
| How the stages of labour are defined, and the expected progress through these | 94% | 6% | 0% |  |  |  |  |  |  |  |  |  |  |  |  |  |  |  |  |  |
| Waters break before labour | 100% | 0% | 0% |  |  | 9% |  |  |  | 91% |  |  |  |  |  |  |  |  |  |  |
| Giving birth to the placenta. | 94% | 0% | 6% |  |  |  |  |  |  |  |  |  |  |  |  |  |  |  |  |  |
| Expected experiences whilst pushing during labour, when about to give birth | 78% | 6% | 17% |  |  | 50% | 39% | 11% |  |  | 82% | 18% | 0% |  |  |  |  |  |  |  |
| Labour complication |  |  |  |  |  |  |  |  |  |  |  |  |  |  |  |  |  |  |  |  |
| Moderate or severe but common complication relating to the mother during labour. | 35% | 25% | 40% |  |  | 53% | 12% | 35% |  |  | 71% | 24% | 6% |  |  | 91% | 0% | 9% |  |  |
| Complication related to the baby during labour. | 93% | 0% | 7% |  |  |  |  |  |  |  |  |  |  |  |  |  |  |  |  |  |
| Possible procedures of interventions during birth |  |  |  |  |  |  |  |  |  |  |  |  |  |  |  |  |  |  |  |  |
| Vaginal examinations offered during labour. | 71% | 29% | 0% |  |  | 87% | 13% | 0% |  |  |  |  |  |  |  |  |  |  |  |  |
| The process of speeding up labour (augmentation of labour). * | 53% | 40% | 7% |  |  | 27% | 73% | 0% |  |  | 38% | 63% | 0% |  |  | 9% | 9% | 0% | 82% * |  |
| When an episiotomy may be offered. | 100% | 0% | 0% |  |  |  |  |  |  |  |  |  |  |  |  |  |  |  |  |  |
| Methods to reduce risk of serious tears to the vagina. | 73% | 20% | 7% |  |  | 79% | 14% | 7% |  |  | 91% | 0% | 9% |  |  |  |  |  |  |  |
| Postnatal procedures |  |  |  |  |  |  |  |  |  |  |  |  |  |  |  |  |  |  |  |  |
| Examination of the vagina and the rectum following birth. | 80% | 20% | 0% |  |  |  |  |  |  |  |  |  |  |  |  |  |  |  |  |  |
| Experiences after birth |  |  |  |  |  |  |  |  |  |  |  |  |  |  |  |  |  |  |  |  |
| Pelvic floor injury that can happen during labour and potential issues with this area following birth..** | 0% | 0% | 0% | 100% ** |  |  |  |  |  |  |  |  |  |  |  |  |  |  |  |  |
| Bowel or bladder symptoms following birth. *** | 0% | 0% | 0% | 100% *** |  |  |  |  |  |  |  |  |  |  |  |  |  |  |  |  |
| After care in the immediate hours following a vaginal birth | 0% | 90% | 0% |  |  |  |  |  |  |  |  |  |  |  |  |  |  |  |  |  |
| Outcomes for baby |  |  |  |  |  |  |  |  |  |  |  |  |  |  |  |  |  |  |  |  |
| Skin-to-skin and attachment of the baby following birth | 58% | 33% | 8% |  |  | 0% | 0% | 0% | - | 100% |  |  |  |  |  |  |  |  |  |  |
| Condition of baby when they are born | 91% | 9% | 0% |  |  |  |  |  |  |  |  |  |  |  |  |  |  |  |  |  |
| Environment during labour |  |  |  |  |  |  |  |  |  |  |  |  |  |  |  |  |  |  |  |  |
| Keeping mobile and adopting different positions in labour. | 64% | 18% | 18% |  |  | 25% | 0% | 0% |  | 75% | 91% |  |  |  | 9% |  |  |  |  |  |
| Medical professionals who may be present in the room during labour. | 58% | 33% | 8% |  |  | 8% | 0% | 0% | - | 92% |  |  |  |  |  |  |  |  |  |  |
| Labour companions who you can choose to have present during labour and their role in the process | 83% | 17% | 0% |  |  |  |  |  |  |  |  |  |  |  |  |  |  |  |  |  |
| What food or drink can be consumed. | 0% | 0% | 0% |  | 100% |  |  |  |  |  |  |  |  |  |  |  |  |  |  |  |

*Information on merged items*

* Merged into information item ‘How the stages of labour are defined, and the expected progress through these’

** Merged into information point ‘Pelvic floor injury that can happen during labour, examination of the area to assess these, and potential issues with this area following birth’

*** Merged into information point ‘Pelvic floor injury that can happen during labour, examination of the area to assess these, and potential issues with this area following birth’
