# Supplementary material for: What Should Be Discussed When Considering a Vaginal Birth? A Delphi Consensus Study
Source: BJOG. 2025 Nov 18;133(3):520–31. doi: 10.1111/1471-0528.70071 (PMC12770075; doi:10.1111/1471-0528.70071)
Supplement: Supplementary file 15 — Table S9: Vaginal birth core information set. [file BJO-133-520-s009.docx]

S13. Vaginal birth core information set

| Vaginal Birth Core Information Set: *What should be discussed with ALL women planning, or considering, a vaginal birth to aid in decision making regarding birth choices...* | |
| --- | --- |
| Labour process |  |
| How the stages of labour are defined, and the expected progress through these. | Stages of labour including latent, first, second & third stage, transition.  What is considered normal timing of progress?  When intervention to help with progress may be offered if not progressing as expected? I.e. Labour augmentation (what this is, how it is done, risks and benefits). |
| Giving birth to the placenta. | How this process happens.  Physiological delivery of the placenta; Medications offered to aid in delivery of placenta to reduce bleeding.  What happens when birth of the placenta is delayed? |
| Expected experiences whilst pushing during labour, when about to give birth. | E.g. Urges to push, involuntary versus spontaneous pushing, stinging sensation, passing wind, bowels opening. |
| Choice of where to give birth (home, midwife led unit, consultant led unit), and when & why may it be recommended to change location during labour. | Factors that may affect where it is recommended that someone give birth (or not give birth).  Which medical professionals will be present depending on location? |
| Pain relief |  |
| Use of medical pain relief in labour including gas & air, oral medications (i.e. paracetamol, dihydrocodeine), injectable medications (i.e. pethidine, diamorphine) & epidural. | When they will be offered, when it is not possible to have, possible labour consequences.  Epidural is a patient-controlled pain relief which numbs from the bottom of the chest to the toes. It is put in by a doctor with an injection of local anaesthetic into the back. |
| Environment during labour |  |
| Labour companions who you can choose to have present during labour and their role in the process. | Who may/may not be allowed to be there and why?  E.g. Birth partners, Doulas. |
| Keeping mobile and adopting different positions in labour. | Factors that may affect ability to adopt different positions. Benefits of adopting different positions. |
| Possible procedures or interventions during labour | |
| Vaginal examinations offered during labour. | The way that a healthcare professional assesses progress during labour.  How and why these are performed? How many may be performed during labour? Who performs these? |
| How a baby’s wellbeing is checked during labour-  Monitoring and procedures. | Monitoring includes listening to heartbeat with a doppler, continuous monitoring (CTG, STAN), small clip attached to baby's head (fetal scalp electrode).  Procedures to check wellbeing include- Fetal scalp stimulation (touching baby's head during a vaginal examination), fetal blood sampling (taking a small sample of blood from baby's head). |
| Methods to reduce risk of serious tears to the vagina. | Guided pushing; Advising panting/puffing when baby's head is visible; Perineal protection- midwife or doctor may ask if they can place hand on the area below the vagina with or without a warm compress; Episiotomy. |
| When an episiotomy may be offered. | An episiotomy is a small cut made at the opening of the vagina at the time of birth. |
| When an instrumental or caesarean birth may be offered or recommended, and why. | Assisted vaginal birth includes forceps (metal spoons on baby's head) or ventouse (suction cup on baby's head) when the neck of the womb is fully open. A caesarean birth (also known as a C-section) is an operation whereby the baby is born through a cut into the abdomen and womb. |
| Possible labour complications |  |
| Complications relating to the mother during labour. | Moderate or severe, but common, complications e.g. Mother having a temperature; Changes in vital parameters (blood pressure, heart rate, urine output); Uterine hyperstimulation (when the womb gets too many contractions in a short space of time).  Complications related to bleeding e.g. Bleeding during labour (antepartum haemorrhage); Placental abruption- when the placenta separates from the womb before birth of the baby. |
| Complication related to the baby during labour. | Moderate but common including- Baby doing a poo inside the womb; Concerns about the baby's heart rate; Uterine hyperstimulation distressing the baby.  Severe but uncommon including- Cord prolapse (the baby's cord coming out of the vagina before the baby is born), shoulder dystocia (the baby's shoulders getting stuck after the head is born); Chorioamnionitis- infection affecting the womb and the baby. |
| Outcomes for the baby |  |
| Condition of baby when they are born. | E.g. Need for resuscitation of the baby; Observations (heart rate, oxygen level etc.) when the baby is born; Blood oxygen levels when born. |
| Experiences after birth |  |
| Possible experiences or symptoms immediately after birth. | E.g. Pain (from tears or after pains from womb contracting); Bleeding; Exhaustion; Anxiety; Urinary incontinence; Reduced amounts or urinating. |
| Feeding of the baby following birth. | Breastfeeding or bottle feeding.  E.g. How long until first feed, factors that can impact this. |
| Pelvic floor injury that can happen during labour, examination of the area to assess these, and potential issues with this area following birth. | Injuries to the pelvic floor includes grazes, bruising or tears to the vagina (different degrees- 1st, 2nd, 3rd, 4th), and tears to the neck of the womb. These may require stitches (may be recommended by midwife or doctor).  Following birth may experience (in the perineal area)- Pain; Change in sensation; Wound breakdown (e.g. stitches unravelling); Infection (may or may not require antibiotics).  Some people may experience short- or long-term bladder or bowel issues (e.g. pain, incontinence). |
| Possible mental health experiences following birth (may be in short and long term). | E.g. Baby blues; Post-traumatic stress disorder; Effect on self-esteem; Postnatal depression; Mental health problems following perineal tear; Fears for future pregnancy/birth. |
