# Supplementary material for: What Should Be Discussed When Considering a Vaginal Birth? A Delphi Consensus Study
Source: BJOG. 2025 Nov 18;133(3):520–31. doi: 10.1111/1471-0528.70071 (PMC12770075; doi:10.1111/1471-0528.70071)
Supplement: Supplementary file 16 — Table S10: Supplementary list of information items with descriptions. [file BJO-133-520-s002.docx]

S14. Supplementary list of information items with descriptions

| Supplementary information list ​: *What should be discussed with ALL women who have chosen to have a vaginal birth to ensure they are informed about potential experiences...* | |
| --- | --- |
| Labour process | |
| The signs and symptoms of labour. | E.g. contractions/tightening, lower back pain, mucus plug, waters breaking, feeling pressure down below. |
| Waters break before labour. | What it means when waters go before being in labour  What to expect, what to do. |
| Environment during labour | |
| Medical professionals who may be present in the room during labour. | Which professionals, how many and why?  E.g. midwives, doctors, student midwives/doctors, midwifery care assistants. |
| What food or drink can be consumed. | When can it be consumed or not? |
| Experiences after birth | |
| Skin-to-skin and attachment of the baby following birth. | Skin-to-skin is the act of putting baby directly onto skin of mother or birth partner following birth.  How soon after birth can this happen, factors that can affect this etc. · How having a vaginal birth may impact bonding of the parents with the baby. |
